# A microphysiological model of human trophoblast invasion during implantation

**DOI:** 10.1038/s41467-022-28663-4

**Published:** 2022-03-15

**Authors:** Ju Young Park, Sneha Mani, Geremy Clair, Heather M. Olson, Vanessa L. Paurus, Charles K. Ansong, Cassidy Blundell, Rachel Young, Jessica Kanter, Scott Gordon, Alex Y. Yi, Monica Mainigi, Dan Dongeun Huh

**Affiliations:** 1 Department of Bioengineering, University of Pennsylvania, Philadelphia, PA USA; 2 Division of Reproductive Endocrinology and Infertility, University of Pennsylvania Perelman School of Medicine, Philadelphia, PA USA; 3 Biological Sciences Division, Pacific Northwest National Laboratory, Richland, WA USA; 4 Division of Neonatology, Children’s Hospital of Philadelphia, Philadelphia, PA USA; 5 NSF Science and Technology Center for Engineering Mechanobiology, University of Pennsylvania, Philadelphia, PA USA; 6 Institute for Regenerative Medicine, Perelman School of Medicine, University of Pennsylvania, Philadelphia, PA USA

**Keywords:** Biomedical engineering, Biological models, Reproductive disorders, Reproductive biology, Proteomic analysis

## Abstract

Successful establishment of pregnancy requires adhesion of an embryo to the endometrium and subsequent invasion into the maternal tissue. Abnormalities in this critical process of implantation and placentation lead to many pregnancy complications. Here we present a microenigneered system to model a complex sequence of orchestrated multicellular events that plays an essential role in early pregnancy. Our implantation-on-a-chip is capable of reconstructing the three-dimensional structural organization of the maternal-fetal interface to model the invasion of specialized fetal extravillous trophoblasts into the maternal uterus. Using primary human cells isolated from clinical specimens, we demonstrate in vivo-like directional migration of extravillous trophoblasts towards a microengineered maternal vessel and their interactions with the endothelium necessary for vascular remodeling. Through parametric variation of the cellular microenvironment and proteomic analysis of microengineered tissues, we show the important role of decidualized stromal cells as a regulator of extravillous trophoblast migration. Furthermore, our study reveals previously unknown effects of pre-implantation maternal immune cells on extravillous trophoblast invasion. This work represents a significant advance in our ability to model early human pregnancy, and may enable the development of advanced in vitro platforms for basic and clinical research of human reproduction.

## Abstract (web summary)

Normal and abnormal pregnancy is challenging to study and involves complex interactions between maternal and fetal cells. Here the authors present an implantation-on-a-chip device capable of modeling trophoblast invasion, a process critical to the establishment of pregnancy.

## Introduction

**Fig. 1:**
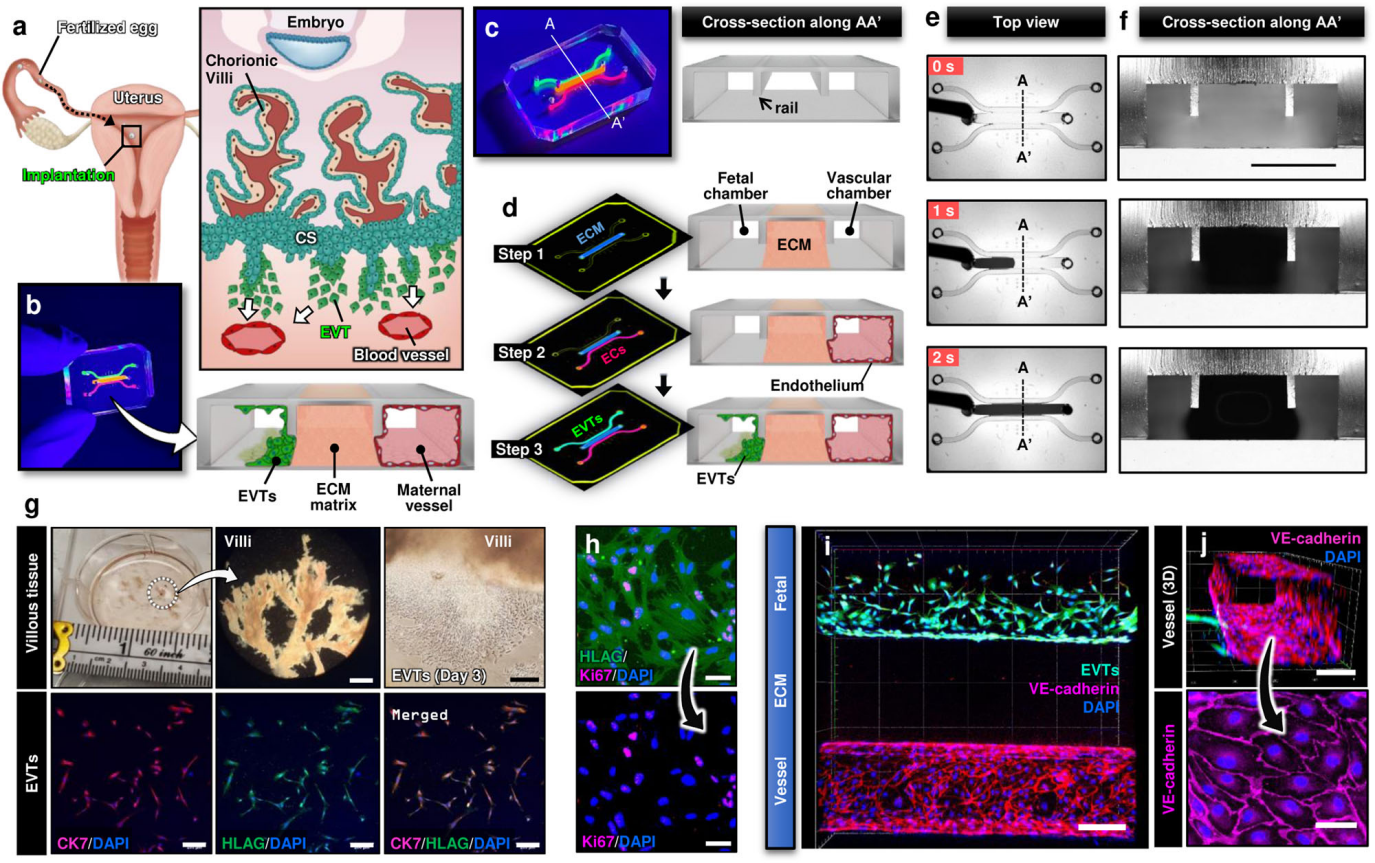
Human implantation-on-a-chip. **a** Soon after implantation, EVTs begin to differentiate from precursor cells in the cytotrophoblast shell (CS) and invade into the uterus, a process that continues through the first half of pregnancy. **b** Compartmentalized design of the implantation-on-a-chip device for in vitro modeling of EVTs and a maternal SA separated by maternal endometrium. **c** Architecture of the implantation-on-a-chip microdevice. The center and two side lanes have dimensions of 0.5 mm (width) × 0.3 mm (height) and 0.25 mm (width) × 0.3 mm (height), respectively. **d** Sequential steps of model construction. **e** Time-lapse imaging of ECM hydrogel precursor (colored black) injection into the center lane of the device. **f** Images of device cross-section to show capillary pinning-based physical confinement of injected hydrogel solution (dark solution) in the center lane. Scale bar, 500 μm **g** (Top row) Photos of first trimester termination tissue and EVT outgrowth from the tissue explants. Scale bars, 1 mm (middle) and 200 μm (right). (Bottom row) The purity of the population was confirmed by immunostaining for cytokeratin 7, a trophoblast marker (magenta) and HLA-G, an EVT-specific marker (green). The representative images of the villous tissue are from five independent experiments. Scale bars, 200 μm. **h** Immunostaining of HLA-G (green) and Ki67 (magenta) expression by EVTs cultured on coverslips in a 6 well plate, after 3 passages. The representative images of EVTs are from four independent experiments. Scale bars, 50 μm. **i** Top-down confocal projection of the microengineered maternal-fetal interface at Day 1. EVTs in the fetal chamber were labeled with CellTracker Green (green). ECs were stained for VE-cadherin (magenta). The representative image is from three independent devices. Scale bar, 200 μm. **j** Endothelial tube in the vascular compartment at Day 1. Magenta and blue show VE-cadherin and nuclear staining, respectively. The representative images are from three independent devices. Scale bars, 100 μm (top) and 50 μm (bottom). *EVTs:* Extravillous trophoblasts, *CS:* Cytotrophoblastic shell, *ECs:* Endothelial cells, *ECM*: Extracellular matrix, *CK7*: Cytokerain 7, *HLA-G*: human leukocyte antigen G.

Pregnancy is an essential process of life that enables the reproduction and propagation of all species. In mammals, pregnancy begins with the fertilization of an egg but its successful establishment is marked by embryo implantation. This involves adhesion and subesquent invasion of the embryo into the maternal endometrium – the specialized mucosal lining of the uterus that supports pregnancy^1^. Shortly after implantation, cells in the trophectoderm comprising the outer layer of the blastocyst fuse to form the primary syncytium that invades the maternal decidua. Subsequently, proliferative cytrophoblasts in the trophectoderm break through the primary syncytium and generate finger-like projections called the chorionic villi that continue to develop and mature to support fetal development throughout pregnancy. At the distal ends of these villi, cytotrophoblasts grow in columns and form elongated masses of cells that merge with those from neighboring villi to generate a structure termed the cytotrophoblast shell (Fig. 1a)^2^. Importantly, during early placental development, cytotrophoblasts in this layer give rise to specialized cells known as the extravillous trophoblasts (EVTs) that are characterized by their invasive phenotype^3^. In the first and early second trimesters, these cells migrate in large numbers into the endometrium and invade maternal spiral arteries (SAs) (Fig. 1a) to remodel them into low resistance vessels and increase vascular supply to the developing fetus^4^. Aberrant changes in the physiological process of EVT invasion and vascular remodeling have been associated with many adverse pregnancy outcomes, such as preeclampsia, intrauterine growth restriction and placenta accreta^5,6^, which together are responsible for over 300,000 maternal deaths and 15 million premature deliveries worldwide every year^7^.

There is accumulating evidence that EVT invasion is influenced by various types of maternal uterine cells including endothelial cells (ECs), immune cells, and decidualized stromal cells^8^. These maternal cells are known to undergo morphological and functional changes in preparation for pregnancy, suggesting that they are poised to regulate implantation even prior to arrival of the embryo^4^. Based on this body of work, increasing efforts are being made to investigate how the maternal cellular environment regulates the invasive behavior of EVTs and how abnormalities in the regulatory process contribute to the pathophysiology of pregnancy complications. Despite considerable progress in pregnancy research, however, conducting these types of studies in the context of human implantation remains a major challenge. Obviously, ethical considerations limit human subject research necessary for in vivo investigation of early developmental processes. While animal models display similarities with human placentation, they are unable to fully recapitulate the depth of EVT invasion, the cellular complexity of the uterus, and the sequence of migratory and remodeling events that occur in humans^9^. As an alternative approach, researchers have developed ex vivo models using human clinical specimens but this strategy has proven problematic for scientific and practical reasons. For instance, tissue sections or placental bed biopsies from term placentas commonly used in ex vivo studies have a significantly different cellular composition than that of the maternal–fetal interface at the initial phase of pregnancy^10^. While it is possible to use the uterus surgically removed from patients for ex vivo studies, this method suffers from the limited availability and viability of surgical specimens and the presence of underlying pathological abnormalities that make it difficult to model normal implantation^11^.

Due to these drawbacks, functional studies of human implantation have relied heavily on using cultured human cells to simulate EVT invasion in vitro. By providing more controllable and accessible model systems amenable to mechanistic investigation, this approach has advanced our cellular and molecular understanding of the invasive behavior of EVTs^12^. The vast majority of existing models, however, utilize transformed or immortalized human trophoblasts that have limited capacity to replicate the phenoype of their in vivo counterparts^5,11^. The fact that these models are constructed using one or two cell types grown in conventional cell culture plates or Transwell inserts also raises the question of whether they are capable of recapitulating the cellular heterogenity of the maternal-fetal interface and the complex, three-dimensional (3D) extracellular environment in which EVT invasion occurs^11^. Taken together, these problems point to a significant unmet need for more predictive and physiologically relevant exprimental models of human implantation.

To address this need, here we present a bioengineering approach that leverages the power of organ-on-a-chip technology for in vitro modeling and quantitative analysis of EVT invasion during human implantation. Specifically, this work demonstrates a microengineered biomimetic model capable of mimicking dynamic biological crosstalk across the maternal-fetal interface that drives EVT invasion and SA remodeling during implantation. Our implantation-on-a-chip is created using primary human EVTs isolated from first trimester placentas and provides a platform to induce, observe, and interrogate in vivo-like 3D migration of fetal trophoblasts towards a microenigneered vessel in the maternal compartment. This system also allows for simulation and direct visualization of EVT-induced endothelial alterations in the maternal artery during SA remodeling. To show the potential of our model for mechanistic investigation of implantation and early placentation, we demonstrate maternal control of trophoblast function by decidual stromal cells and examine its biological underpinnings through proteomic analysis. By incorporating clinical isolates of human endometrial natural killer cells obtained from pre-implantation uterine tissue, our study also provides in vitro evidence suggesting the significant role of maternal immune cells present prior to embryo implantation in the establishment of pregnancy.

## Results

### Design and production of implantation-on-a-chip

Our microphysiological implantation-on-a-chip model is created in a 3D microfluidic device designed to reproduce physiological compartmentalization of different cell types in the maternal-fetal interface during implantation and early placentation (Fig. 1b). The device consists of three parallel lanes defined by two micropatterned rails protruding from the ceiling of the device (Fig. 1c). Model construction begins with the injection of extracellular matrix (ECM) hydrogel precursor into the center lane and its gelation to create a 3D hydrogel scaffold that mimics the specialized maternal endometrium underneath an implanting embryo (Step 1 in Fig. 1d). This step also results in the formation of two individually addressable microchannels on the sides of the ECM hydrogel construct that represents the fetal aspect, and the maternal vasculature (Step 1 in Fig. 1d). Next, primary human uterine ECs are seeded into the vascular chamber and allowed to form a continuous endothelial lining along the exposed hydrogel surface and channel walls in order to generate a fully endothelialized, perfusable channel that mimics a maternal spiral artery (Step 2 in Fig. 1d). This is followed by seeding of human EVTs into the fetal compartment, during which the device is held vertically to allow the cells to attach to the hydrogel surface and mimic cell columns that form at the tip of the placental villi, from which EVTs invade (Step 3 in Fig. 1d). Completion of these sequential steps yields a 3D cellular distribution reminiscent of the spatial arrangement of fetal trophoblasts and a maternal spiral artery prior to the initiation of EVT invasion.

To demonstrate the physical implementation of our device design and production method, we prepared a microfluidic three-lane device made out of poly(dimethylsiloxane) (PDMS) and used it for testing the formation of the ECM hydrogel layer. When we injected a mixture of collagen and Matrigel into the empty device, the solution was observed to advance along the center lane in a stable manner (Fig. 1e; Supplementary Movie 1). Microscopic examination of the device cross-section revealed that the microfabricated rails at the channel ceiling served to pin the injected liquid, presumably due to surface tension, and confine it to the center lane (Fig. 1f; Supplementary Movie 2). This phenomenon known as capillary pinning^13^ provided an effective means to prevent unwanted liquid spillage to the neighboring lanes, making it possible to create spatially defined 3D ECM tissue barriers in our model.

Next, we used the hydrogel-containing devices for demonstrating compartmentalized microfluidic cell culture. Among the major advantages of our model is its high cellular fidelity achieved by the use of primary human cells. In particular, EVTs were obtained from first trimester termination tissue between 6 and 12 weeks gestation (top panels, Fig. 1g) and cultured in flasks up to passage 3 prior to seeding into the device. This initial culture yielded a highly pure (98%) EVT population without mesenchymal contamination as demonstrated by immunostaining HLA-G, which is a marker of EVT identity (bottom panels, Fig. 1g)^14^. Our analysis showed that approximately 60% of the HLA-G-expressing cells stained positive for Ki67 – a well-characterized nuclear marker expressed in all active phases of the cell cycle (Fig. 1h), illustrating the presence of a proliferative EVT population. Primary ECs isolated from the endometrium prior to pregnancy were commercially obtained and used for creating a microengineered maternal blood vessel. Within 2 h of seeding, EVTs and ECs introduced into our device adhered firmly to the hydrogel surface in the fetal chamber and the fibronectin-coated microchannels in the vascular chamber, respectively and maintained their proliferative capacity during culture to populate their respective compartments (Fig. 1i). 1 day after seeding, the ECs grew to confluent monolayers on all channel surfaces, eventually forming a tube-like structure in the vascular compartment that expressed adherens junctions (Fig. 1j). Given that trophoblasts in the first trimester invade into maternal blood vessels and form plugs in the vascular lumen that prevent blood flow into the intervillous space^2^, the microengineered endothelium was maintained under static conditions to simulate the physiological environment of the SA in early pregnancy.

### EVT invasion in the implantation-on-a-chip

**Fig. 2:**
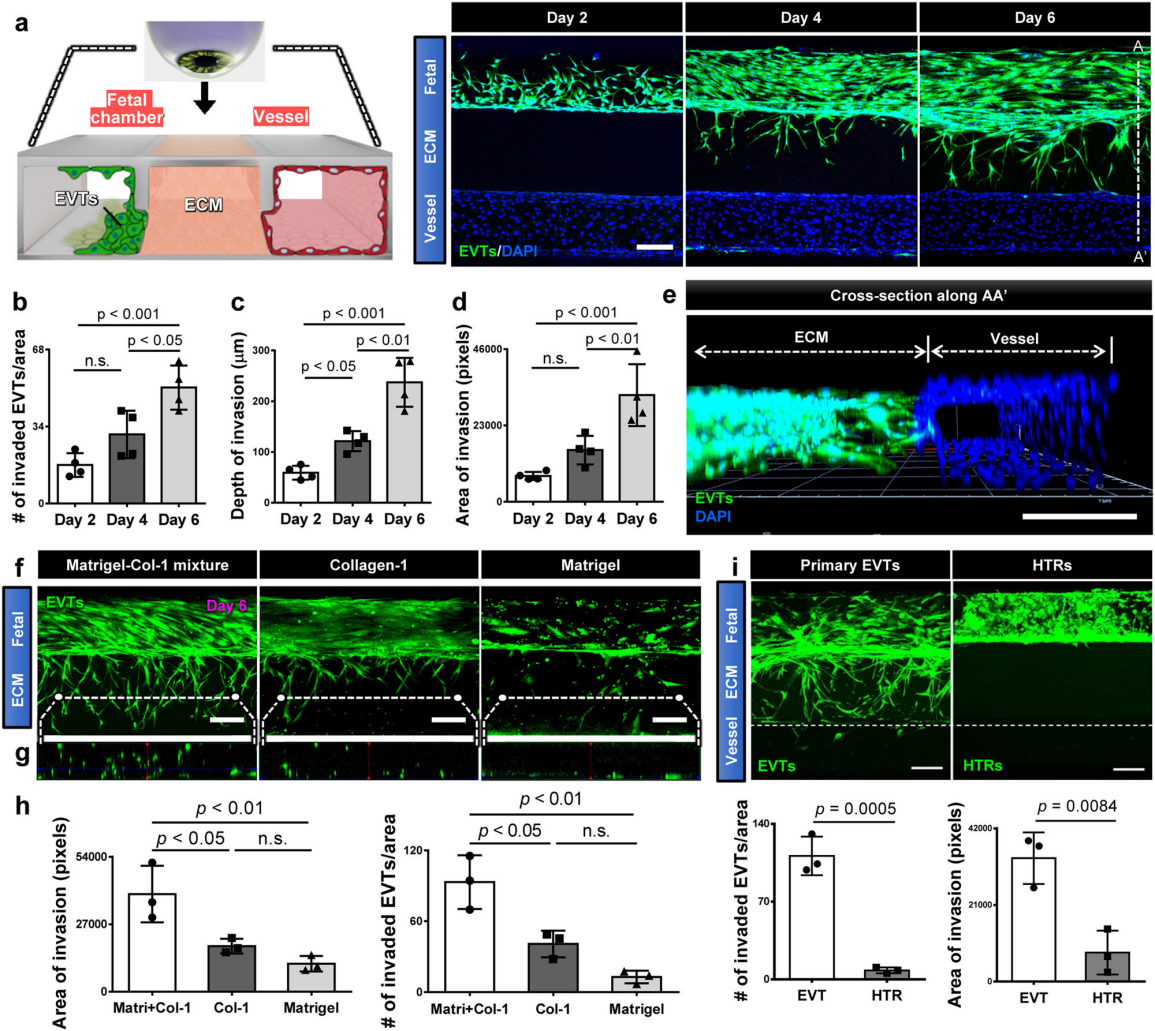
EVT invasion in the implantation-on-a-chip. **a** Top-down view of the device containing fetal EVTs and maternal ECs visualized at Days 2, 4, and 6. The representative images are from four independent devices. EVTs were stained with CellTracker Green and DAPI, whereas ECs are shown with DAPI staining alone. Scale bar, 200 μm. **b**–**d** Quantification of EVT invasion. One-way ANOVA with Tukey’s multiple comparison test (*n* = 4 independent devices). **e** Confocal rendering of 3D EVT migration in the ECM scaffold towards the maternal vessel. The representative image is from three independent devices. Scale bar, 200 μm. **f** EVT invasion in three ECM conditions. Images were taken at Day 6. Scale bars, 200 μm. **g** Cross-sectional view showing 3D distribution of invading EVTs (green) across the thickness of each hydrogel construct. The representative images of **f**, **g** are from three independent experiments. **h** Quantification of invasion area (left) and the number of invading EVTs (right). One-way ANOVA with Tukey’s multiple comparison test (*n* = 3 independent devices per group). **i** Comparison of ECM invasion by primary EVTs and HTR-8/SV neo cells (HTRs) after 6 days of coculture with ECs. ECs in the vascular chamber were not fluorescently labeled and are therefore not visible in the images. The representative images are from three independent experiments. Scale bars, 200 μm. Invasive activity was evaluated by the number of invading cells and area of invasion at Day 6. Two-sided *t*-test (*n* = 3 independent devices per group). Data are presented as mean ± SD. *p* values are shown on graphs. n.s.; not significant. Source data are provided as a Source Data file.

Our established model was then used to examine the behavior of EVTs in the microengineerd physiological environment during prolonged culture. During the first two days of culture, EVTs expanded in number and grew out of the exposed ECM hydrogel surface that they were seeded on, while remaining in the fetal chamber (left panel, Fig. 2a). As culture progressed, however, many cells accumulated at the hydrogel interface and began to sprout into the ECM scaffold (middle panel, Fig. 2a), which became more pronounced over time as evidenced by the increasing number of trophoblasts advancing through the hydrogel compartment (right panel, Fig. 2a, b). Our data also indicated that the depth and area of EVT invasion increased with time (right panel, Fig. 2a, c, d). The cells at the leading edge of the invading population reached the vascular compartment within 5-6 days of culture (right panel, Fig. 2a). The majority of the HLA-G expressing EVTs (82%) migrating through the ECM hydrogel retained their proliferative capacity as demonstrated by Ki67 expression (Supplementary Fig. 1). Confocal microscopy clearly showed three-dimensionality of EVT invasion with very few cells migrating on the top and bottom channel surfaces (Fig. 2e).

EVT invasion and migration also induced measurable changes in the mechanical properties of the microengineered maternal tissue. In particular, the Young’s modulus of the ECM hydrogel scaffold increased from 390.24 ± 146.25 Pa prior to EVT invasion, which is within the physiolgical range of tissue stiffness in nonpregant endometrium^15^, to 826.26 ± 164.84 Pa after 6 days of culture, which is comparable to the measurement of human decidua basalis in the first trimester (Supplementary Fig. 2)^15^. This result demonstrates significant tissue stiffening due to invading EVTs, which is in agreement with the findings of previous work using ex vivo human tissues^15^. Recognizing the important role of the uterine ECM in directional EVT invasion, we then examined how the ECM composition of the hydrogel scaffold affected EVT invasion in our model. The placental bed is known to be composed mostly of laminin and some fibronectin, which are the main components of Matrigel used as the ECM scaffold in our implantation model, while collagen is the primary ECM component in the outermost layer of the spiral artery and perivasuclar regions^16^. Based on these findings, we compared EVT invasion in three ECM conditions—(i) Growth Factor-Reduced (GFR) Matrigel only; (ii) type I collagen only; and (iii) an equal mixture of GFR Matrigel and type I collagen, which is the condition used in our demonstration above. In this study, trophoblast migration into the hydrogel compartment was observed in all three conditions (Fig. 2f). Our analysis, however, showed that EVT invasion into collagen or Matrigel alone occurred to a significantly lesser extent than was measured in the Matrigel–collagen mixture group (Fig. 2f, h). It was also noted that the Matrigel only condition led to 2D cell migration mostly on the bottom surface of the channel, whereas the other two conditions, especially the Matrigel–collagen mixture, supported 3D distribution of the invading EVTs across the thickness of the hydrogel construct (Fig. 2g). These results allowed us to identify the Matrigel–collagen mixture as an optimal, physiologically relevant ECM condition for in vitro modeling of EVT invasion in our device.

A major hurdle in the study of early pregnancy is the difficulty in obtaining human tissue for use in experimental research. In order to determine whether commercially available cell lines could be substituted for primary EVTs, we repeated these experiments with HTR-8/SV neo cells, a transformed cell line derived from first-trimester villous explants that is commonly used for the study of fetal trophoblasts in early pregnancy^17^. Despite their EVT-like characteristics, HTR-8 cells cultured in the presence of maternal ECs and maintained in the same conditions in our device exhibited notably different behavior compared to their primary cell counterparts. Although the cells continued to proliferate actively in the fetal chamber, we did not detect any measurable cell migration into the hydrogel compartment made out of a Matrigel–collagen mixture (Fig. 2i). Some of the cells formed thin cellular processes across the interface between the culture chamber and the ECM scaffold but their extension deep into the hydrogel was not observed over the course of 6-day culture (Fig. 2i), even when the concentration of serum in the media was increased (Supplementary Fig. 3). The lack of cell migration in these experiments is in contrast to active invasion of HTR-8 cells reported using Transwell assays that are usually carried out on a thin Matrigel or gelatin matrix with significantly lower hydrogel concentration and stiffness^11^.

Taken together, these results demonstrate that by using primary human trophoblasts from early pregnancy cultured in a physiologically relevant microenvironment, our implantation model is capable of appropriately simulating directional invasion of EVTs into the uterus towards maternal spiral arteries.

### Regulation of EVT invasion by maternal endothelial cells

**Fig. 3:**
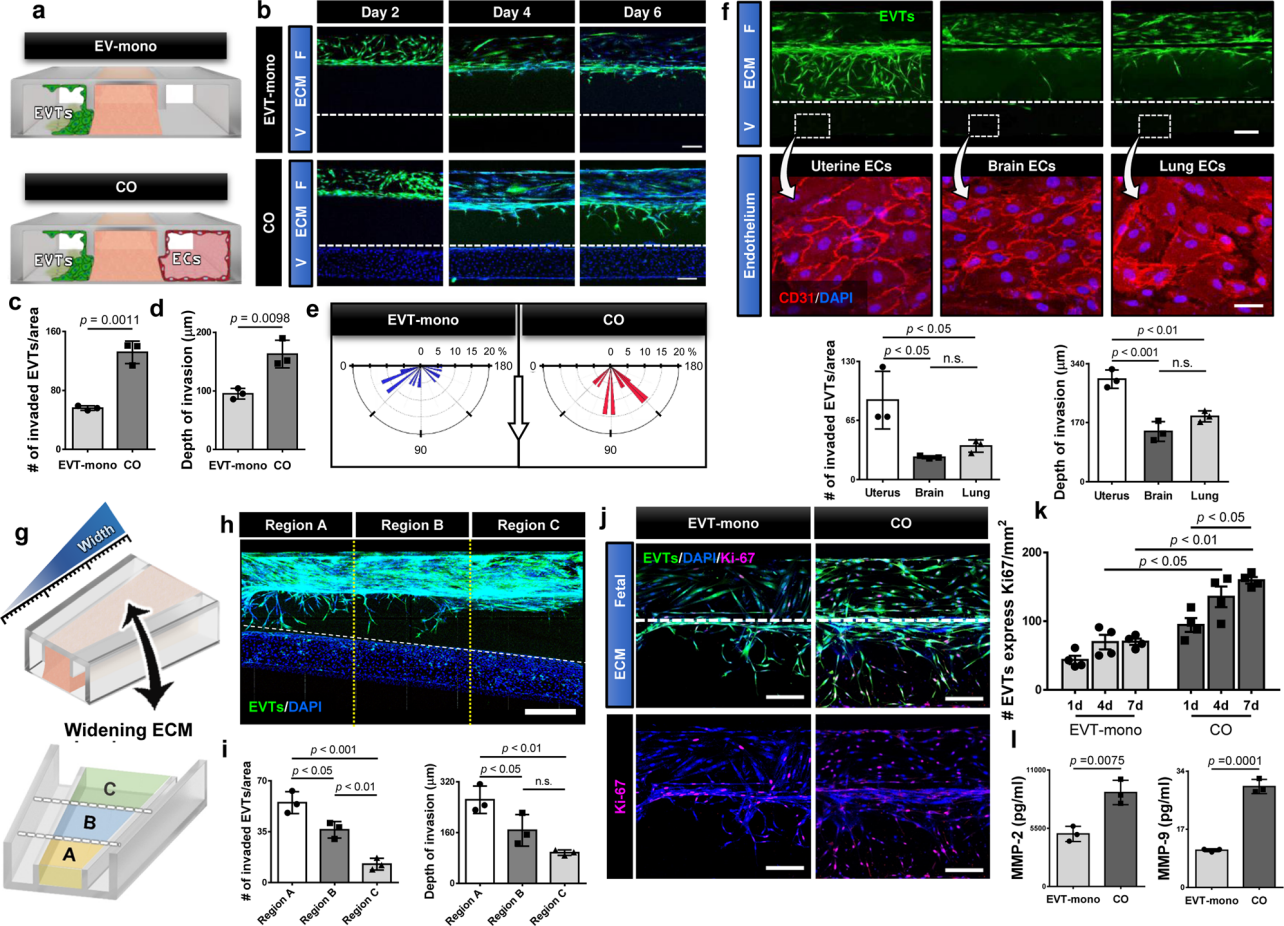
Endothelial regulation of EVT invasion. **a** Endothelial contribution to EVT invasion was examined by comparing monoculture of EVTs to coculture of EVTs and ECs. **b** Top-down images of EVT migration at Days 2, 4, and 6. EVTs in the fetal chamber (F) were stained with CellTracker Green and DAPI. ECs in the vascular chamber (V) were visualized by DAPI nuclear staining. Dashed lines indicate the boundary between the ECM hydrogel and the vascular compartment. The representative images are from three independent experiments. Scale bars, 200 μm. Quantification of **c** the number of invading cells, **d** invasion depth. Two-sided t-test (*n* = 3 independent devices per group). The rose plots in **e** show the distribution of orientation angles between the major axis of invading cells and the horizontal line. The length of each spoke around the half-circle is proportional to the percentage of cells. The white arrow at the center indicates the direction pointing from the fetal to vascular chamber. (The plots indicate the orientation of 14 to 16 EVTs randomly selected from a representative device per group. The same trends were confirmed in 3 independent devices per group.) **f** Invasion of EVTs labeled with CellTracker green in coculture with different types of primary human ECs after 6 days of culture. Bottom panels show the endothelium with CD31 staining (red) in the vascular chamber. The representative images are from three independent experiments. Scale bars, 200 μm (top) and 50 μm (bottom). Invasive capacity was quantified by the number of invading cells and invasion depth at Day 6. One-way ANOVA with Tukey’s multiple comparison test (*n* = 3 independent devices per group). **g** A microdevice containing a widening central ECM chamber was used to evaluate the effect of distance between the fetal and vascular chambers on EVT migration. **h**, **i** Imaging and quantification of EVT invasion in three separate regions at Day 6. The ranges of channel width are 500–650 μm (Region A), 675–825 μm (Region B), and 850–1000 μm (Region C). The representative image is from three independent experiments. Scale bar, 600 μm. One-way ANOVA with Tukey’s multiple comparison test (*n* = 3 independent devices). EVT proliferation was evaluated by **j**, **k** immunostaining of Ki67 (magenta). The representative images are from three independent experiments. Scale bars in **j**, 200 μm. Two-way ANOVA with Sidak’s multiple comparison test (*n* = 3 independent devices). **l** Comparison of MMP-2 and 9 production measured by ELISA. Two-sided *t*-test (*n* = 4 independent devices per group). Data are presented as mean ± SD. *p* values are shown on graphs. n.s.; not significant. Source data are provided as a Source Data file.

In the next phase of our study, we set out to explore the potential of our model as an in vitro platform for studying the contribution of maternal cells to EVT invasion, which represents a topic of active research in implantation and placental biology^18^. To this end, we first investigated the role of vascular ECs in maternal spiral arteries by culturing EVTs in the absence (monoculture) or presence (coculture) of uterine ECs in the maternal vascular compartment (Fig. 3a).

The key finding of this study was that in comparison to the coculture system demonstrated in Fig. 2, trophoblast invasion was drastically reduced when the model did not include the microenigneered materal artery (Fig. 3b). Quantification of EVT invasion at Day 6 indicated 2.85- and 1.69-fold decreases in the number of invading cells (Fig. 3c) and invasion depth (Fig. 3d) in the monoculture system, respectively. Our analysis also showed a significant loss of directionality in EVT migration in the absence of the endothelialized blood vessel (Fig. 3e). These data were indicative of biological crosstalk in which maternal ECs interact with EVTs through the ECM hydrogel to play a causative role in their invasion and directional migration. Importantly, when EVTs were co-cultured with primary human ECs obtained from other organs such as the brain and the lung, there was a significant decrease in their ability to invade the hydrogel compartment (Fig. 3f), illustrating the tissue-specificity of trophoblast-endothelial cell interactions in our model.

To further investigate maternal-fetal crosstalk, we established coculture of EVTs and uterine ECs in a microdevice that contained a widening central ECM chamber (Fig. 3g). This design allowed us to evaluate the effect of physical proximity between the two cell populations on EVT invasion. Analysis of cell migration in three non-overlapping regions of the ECM compartment revealed that the number of invading EVTs decreased proportionally with increasing distance between the fetal chamber and the maternal blood vessel (Fig. 3h, i). The same trend was noted in the depth of EVT invasion (Fig. 3i). Collectively, these observations imply that intercellular communication between maternal ECs and EVTs may rely on diffusion-mediated transport of soluble factors across the ECM hydrogel.

Having demonstrated significant endothelial contributions to EVT invasion, we then conducted additional characterization of EVTs in the monoculture and coculture systems to better understand how the maternal vasculature modulates fetal trophoblasts during implantation. In this analysis, we first examined EVT proliferation using Ki67. In the presence of ECs in the vascular compartment, there was an increase in the number of Ki67-positive cells as compared to monoculture control (Fig. 3j, k), indicating vascular contribution to increasing the proliferative capacity of EVTs. This increase in proliferative activity was apparent 1 day after cell seeding and persisted throughout the culture period (Fig. 3k). Furthermore, ELISA showed that the co-culture configuration resulted in significantly elevated levels of matrix metalloproteinases (MMP)-2 and 9 (Fig. 3m), which have been shown to play a critical role in enhancing the invasive capacity of EVTs at the maternal–fetal interface^19^.

### Mimicking spiral artery remodeling in the implantation-on-a-chip

When invading EVTs reach SAs in the uterus, they induce physiological transformation of the maternal arteries into dilated vessels with low flow resistance, which is essential for establishing placental perfusion required for efficient exchange of nutrients and waste products between maternal and fetal circulation. This remodeling occurs through the first trimester of pregnancy and involves EVT-induced changes to the cellular and extracellular constituents of spiral arteries^4^. Importantly, vascular disruption^20^ and apoptosis^21^ of the vascular endothelium represent two main features of this dynamic process. Therefore, we investigated whether these characteristic physiological events take place as a result of EVT invasion in our device.

**Fig. 4:**
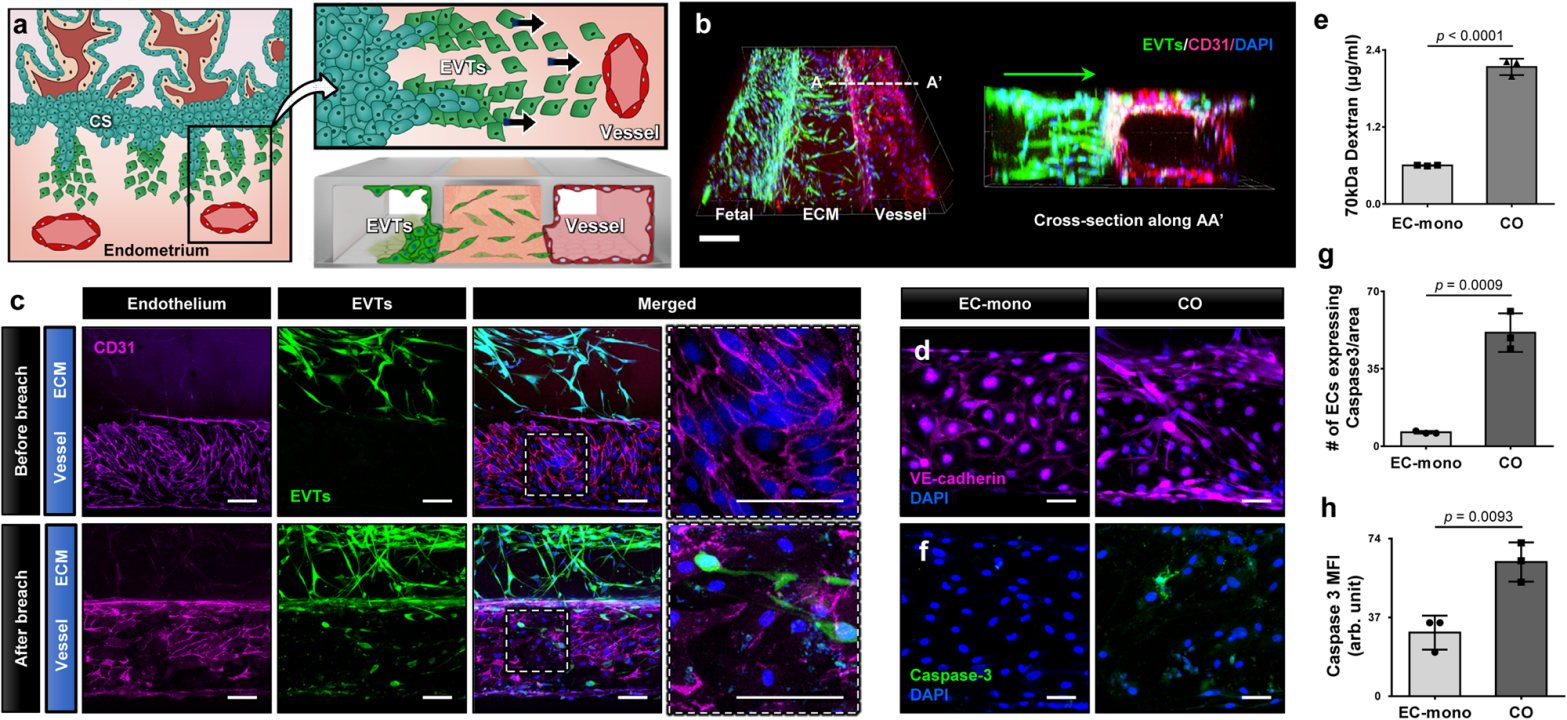
Remodeling of maternal vessel in the implantation-on-a-chip. **a** Interactions between invading EVTs and maternal blood vessels were modeled in the implantation-on-a-chip. **b** 3D projection images of EVTs (green) migrating across the ECM hydrogel towards maternal ECs (magenta). The representative images are from three independent experiments. Scale bar, 200 μm. **c** Invasion of the vascular endothelium by EVTs. Top and bottom panels show before and after endothelial breach, respectively. The representative images are from 3 independent experiments. Scale bars, 100 μm. Comparison of vascular integrity in monoculture control (ECs only) and coculture (EVTs + ECs) models based on **d** immunostaining of adherens junctions (magenta) and **e** endothelial barrier permeability to 70 kDa FITC-dextran. The representative images are from three independent experiments. Two-sided *t*-test (*n* = 3 independent devices per group). Blue in **d** shows nuclear staining. Scale bars, 50 μm. **f**–**h** Visualization and quantification of caspase-3 (green) expression. Scale bars, 50 μm. The representative images are from three independent experiments. Two-sided t-test (*n* = 3 independent devices per group). Data are presented as mean ± SD. *p* values are shown on graphs. EC-mono: Endothelial cell monoculture, CO: EVT/EC coculture. Source data are provided as a Source Data file.

In this study, we utilized fluorescence microscopy to examine whether and how invading EVTs interact with the micro-engineered maternal artery (Fig. 4a). During culture, EVTs entering the ECM scaffold continued to migrate across the hydrogel in a directional manner and eventually arrived at the maternal vessel as early as at Day 5. These cells then began to breach the vascular compartment by penetrating the vessel walls and situating themselves in the endothelial lining of the culture chamber (Fig. 4b, c). Notably, this invasive behavior of EVTs resulted in rapid disruption of the vascular endothelium. In comparison to prior to EVT invasion or the control group established by monoculture of an endothelialized vessel maintained under the same media conditions for the same duration, the endothelium breached by trophoblasts appeared more disorganized and showed markedly reduced expression of VE-cadherin (Fig. 4c, d), indicating a loss of vascular integrity due to invading EVTs. Disruption of the endothelial barrier in this model was further evidenced by increased vascular permeability as measured by the passage of FITC-Dextran from the maternal to fetal compartments (Fig. 4e).

Our data also demonstrated the capacity of EVTs to induce endothelial cell apoptosis. In the endothelial monoculture control without trophoblasts, the maternal endothelium remained intact and exhibited minimal expression of caspase-3 (left column, Fig. 4f). In contrast, inclusion of EVTs and their invasion into the vasculature resulted in the activation and upregulation of apoptotic pathways (right column, Fig. 4f), which was shown quantitatively by significant increases in the number of caspase-3-positive ECs (Fig. 4g) and their caspase expression on a per-cell basis (Fig. 4h). These results are consistent with previous findings that EVTs replace maternal ECs during SA remodeling by inducing endothelial apoptosis in a caspase-dependent manner^21^. This validation of physiological relevance also supports the feasibility of recapitulating the key steps of SA remodeling in the implantation-on-a-chip.

### Effect of decidualized stromal cells on EVT invasion

**Fig. 5:**
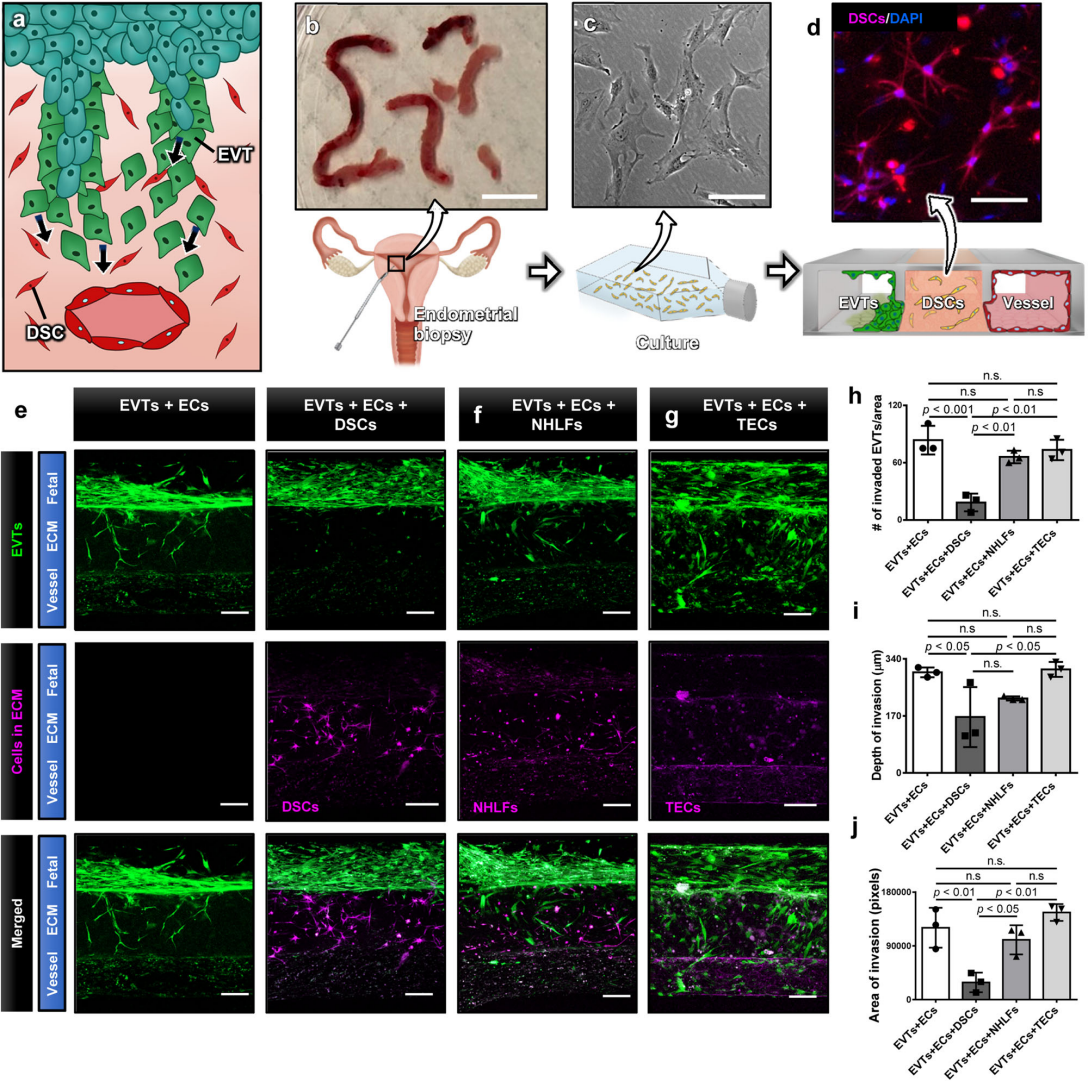
Effect of decidualized stromal cells on EVT invasion. **a** During invasion, fetal EVTs encounter DSCs and interact with them. **b** Primary human DSCs were isolated from pre-pregnancy endometrial biopsies taken during the window of implantation. Scale bar, 1 cm. **c** Isolated DSCs were cultured in a flask prior to seeding into the ECM compartment of the implantation-on-a-chip device. The representative image is from three independent experiments. Scale bar, 100 μm. **d** A micrograph of DSCs (magenta) in the hydrogel. Cell nuclei were stained with DAPI (blue). The representative image is from three independent experiments. Scale bar, 100 μm. **e** Comparison of EVT migration towards ECs in the absence (left colum) or presence (right colum) of DSCs. Scale bars, 200 μm. Tissue-specificity of the observed effects was investigated by replacing DSCs with **f** NHLFs and **g** TECs in the ECM compartment. The representative images of (e-g) are from three independent experiments. **h**–**j** Quantification of EVT invasion under different combinations of cultured cells. Data are presented as mean ± SD. One-way ANOVA with Tukey’s multiple comparison test (*n* = 3 independent devices per group). *p* values are shown on graphs. DSCs, Decidual stromal cells. NHLFs: Normal human lung fibroblasts, TECs: Tracheal epithelial cells Source data are provided as a Source Data file.

During the luteal phase of the menstrual cycle, the endometrium undergoes a sequence of dynamic structural and functional changes that starts around the SAs, termed decidualization, to generate a receptive environment for implantation^22^. As part of this process, stromal cells in the endometrium lose their fibroblast-like phenotype and transform into secretory, epithelioid cells after which they are called decidualized stromal cells (DSCs)^22^. DSCs make up the bulk of the uterus and are known to interact with EVTs (Fig. 5a), playing a major role in their invasion^23^. Research has suggested the role of DSCs as “gatekeepers” that serve to limit and antagonize EVT migration through the production of inhibitory factors such as tissue inhibitors of metalloproteinases (TIMPS)^24^ Studies, however, have also shown that DSCs can promote EVT invasion by stimulating proteolytic degradation and expression of cell surface proteins that facilitate EVT adhesion and migration^25^. Motivated by this controversy, we examined the effect of DSCs on the invasive phenotype of EVTs using our implantation-on-a-chip model.

For this study, we established a protocol for isolating primary human DSCs from biopsy specimens of pre-pregnancy, mid-secretory phase endometrium, which is primed to support implantation and direct early trophoblast invasion (Fig. 5b, c). Decidualization was maintained in these cells through the addition of medroxyprogesterone acetate (MPA), estradiol (E_2_), and cAMP in culture media during the isolation and expansion process, after which decidualized cells were seeded into the culture chamber and embedded in the ECM hydrogel. The fetal and vascular compartments on the sides were seeded with EVTs and uterine ECs, respectively. The 3D ECM microenvironment of our device supported spreading and proliferation of the DSCs and maintained their viability (Fig. 5d). Throughout the device culture period, these cells remained in the ECM compartment without migrating out of the hydrogel scaffold and maintained their decidualized phenotype without the supply of MPA, E_2_, and cAMP in the media, as shown by robust expression of prolactin by DSCs in the device after 6 days of culture (Supplementary Fig. 4).

Importantly, the introduction of DSCs into our model led to significant changes in the behavior of EVTs. When compared to the coculture model containing EVTs and maternal ECs (left column, Fig. 5e), we observed considerably reduced trophoblast sprouting into the DSC-laden hydrogel (right column, Fig. 5e). It was also noted that accumulation of EVTs on the exposed hydrogel surface in the fetal compartment occured to a lesser extent in the presence of DSCs (Fig. 5e). The noticeable differences between these two groups were corroborated by the significantly decreased number of invading EVTs (Fig. 5h), invasion depth (Fig. 5i), and the area of invasion (Fig. 5j) in the DSC-containing devices.

Of note, this inhibitory effect of DSCs on trophoblast invasion occurred in an organ-specific manner. When primary normal human lung fibroblasts (NHLFs) were cultured in the ECM compartment, EVT invasion of the hydrogel was increased, resembling the extent of invasion seen in coculture conditions (Fig. 5f, h–j). The same experiment was also conducted using primary human tracheal epithelial cells (TECs) as a cell type that represents a non-stromal population. When cultured in the ECM scaffold at similar densities, these cells did not impede trophoblast invasion, and we observed active, directional migration of EVTs (Fig. 5g), which was statistically indistinguishable from what was observed in the original EVT-endothelial coculture group (Fig. 5h–j). Analysis of the hydrogel compartment after 6 days of culture indicated no statistically significant difference in the key components and stiffness of the ECM scaffold between the DSC-containing model and the other groups (Supplementary Fig. 5), suggesting that cell-specific biological activity of DSCs, rather than changes in the ECM environment, contribute to the inhibitory effect of DSCs on EVT invasion.

### Secretomics and Proteomic analysis of trophoblast invasion

**Fig. 6:**
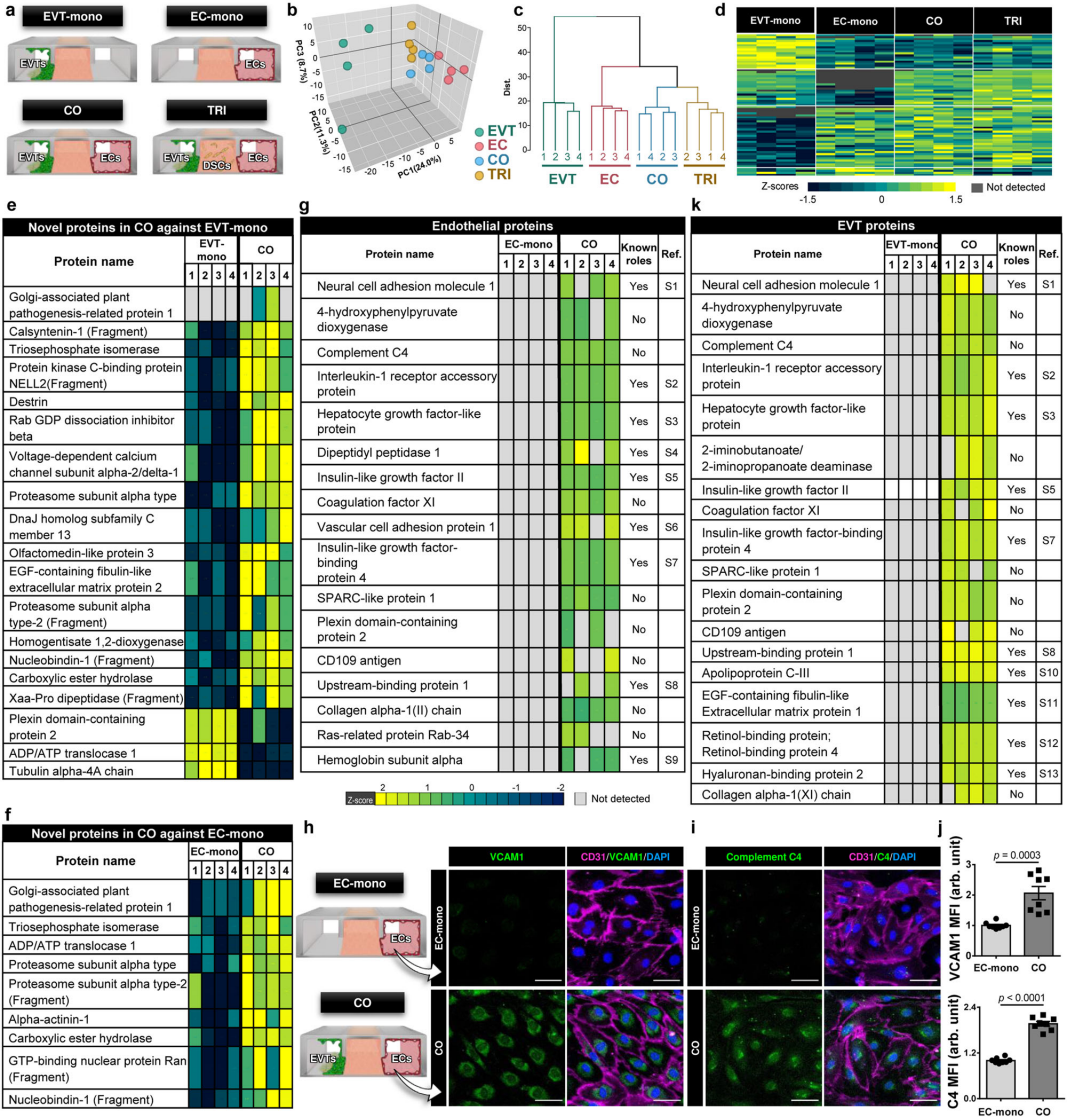
Secretomic and Proteomic analysis of implantation on-a-chip. **a** Four different culture conditions investigated in proteomics analysis. **b** Plots generated by principal component analysis performed across all quantifiable proteins (*n* = 498) from 4 biological replicates for each of the tested conditions. Each data point represents an individual biological replicate. **c** Heirarchical clustering plot. **d** Heatmap depicting the human proteins significantly modulated in abundance in the different tested conditions (one-way ANOVA *p* < 0.05). **e**, **f** 19 and 9 proteins without known function in implantation from the comparison of secretome **e** between CO and EVT-mono and **f** between CO and EC-mono, respectively. **g** 17 uniquely expressed endothelial proteins in the coculture configuration of the implantation-on-a-chip, including 8 proteins with unknown function in implantation and placental development. The left-most column named Ref. shows references in Supplementary Information. Immunofluorescence of **h** VCAM-1 and **i** C4 in the vascular endothelium in the implantation-on-a-chip at Day 6. The representative images are from three independent experiments. Scale bars, 50 μm. **j** Quantification of immunofluorescence of VCAM-1 and C4 staining. Data were presented as mean ± s.d. Two-sided t-test (*n* = 8 images taken from four independent devices per group). *p* values are shown on graphs. **k** 18 uniquely induced EVT proteins in coculture, including 8 proteins with previously unknown roles in implantation/placentation. The left-most column named Ref. shows references in Supplementary Information. Source data are provided as a Source Data file.

Having demonstrated that maternal uterine cells have the capacity to regulate trophoblast invasion, we set out to gain a deeper understanding into the molecular basis of these results. A significant amount of work has been done to examine key mediators of molecular pathways that play an important role in maternal control of implantation^18^. Many of these studies, however, often rely on targeted analysis of pre-selected candidate molecules at the RNA level using traditional cell culture models with limited capacity to account for multicellularity and the 3D microenvironment of the maternal-fetal interface. Our work aimed to address some of these limitations by conducting more comprehensive, unbiased analysis of protein expression in the implantation-on-a-chip. To this end, we first examined the secretome of our model to identify soluble factors that mediate maternal control of EVT invasion. This study was carried out by measuring secreted proteins in the effluent of devices maintained in four different culture conditions for 6 days: (i) EVT monoculture (EVT-mono), (ii) endothelial cell monoculture (EC-mono), (iii) EVT-endothelial coculture (CO), and (iv) triculture of EVTs, ECs, and DSCs (TRI) (Fig. 6a, Supplementary Fig. 6).

The principal component analysis (PCA) of our data showed the clustering of biological replicates for each of the tested conditions (Fig. 6b). Notably, clear separation was observed along the first component of the PCA between the secretome of EVT-mono and that of all the other conditions that contained ECs in common (i.e. EC-mono, CO, and TRI) (Fig. 6b). Consistent with this finding, hierarchical clustering (Fig. 6c) and heat map visualization of protein expression in these groups (Fig. 6d) showed that the greatest differences between the measured secretomes resulted from the presence of the endothelial population, suggesting a critical role of ECs in regulating EVT invasion. Differences were visible in the three EC-containing groups but they seemed minor compared to the noticeable changes observed in the EVT-mono group (Supplementary Figs. 7, 8).

Based on this observation, we next compared the secretome of CO against that of EVT-mono to examine proteomic changes due to the inclusion of ECs in our model. The results of this analysis revealed 7 proteins expressed only during coculture, and 46 differentially regulated proteins in the coculture group compared to EVTs only, 30 of which were upregulated and 15 of which were downregulated (Supplementary Fig. 9). Many of these proteins, such as Cadherin 6^26^, Metalloproteinase inhibitor 1^27^, Annexin A5^28^, and histone H2B^29^, have previously been shown to be present at the maternal-fetal interface and play a role in trophoblast function and placental development, demonstrating the in vivo relevance of the secretome of our model. However, analysis also yielded 19 proteins without known function in implantation and placentation (Fig. 6e). For example, nucleobindin-1, one of the overexpressed markers in the coculture model, is a multidomain Golgi protein that plays an important role in calcium homeostasis^30^ but its function in implantation and early pregnancy has not been shown previously. The other proteins included olfactomedin-like protein 3, EGF-containing fibulin-like extracellular matrix protein 2, proteasome subunit alpha type-2, homogentisate 1,2-dioxygenase, Xaa-Pro dipeptidase, and carboxylic ester hydrolase. Similar analysis comparing CO against EC-mono identified 29 differentially expressed proteins (Supplementary Fig. 10), 9 of which have not been implicated in early pregnancy (Fig. 6f).

To delve further into the proteome of the implantation-on-a-chip, we then expanded the scope of our work beyond the analysis of secreted products to investigate how protein expression changes in ECs and EVTs due to the coculture configuration of our model. In this study, we first undertook proteomics analysis of ECs and EVTs isolated from the EC-mono and EVT-mono groups, respectively, after 6 days of device culture. The results were then compared to the analysis of sorted populations of ECs and EVTs harvested from coculture devices after the same duration of culture. This comparison identified 17 uniquely expressed (Fig. 6g) and 14 significantly upregulated endothelial proteins (Supplementary Fig. 11) in coculture conditions. Among these markers, for example, vascular cell adhesion protein 1 (VCAM-1) has previously been shown to be reduced in pregnancies complicated by preeclampsia or intrauterine growth restriction and regulate the invasion ability of HTR-8 cells in vitro^31,32^. In our analysis, VCAM-1 was detected only when ECs were grown with EVTs to simulate maternal-fetal interactions. This finding was verified by immunofluorescence staining of endothelial VCAM-1 in coculture devices (Fig. 6h, j). Importantly, 8 of the 17 unique protein markers of coculture have not been associated with implantation, suggesting their potential as candidate regulators of maternal control of EVT invasion. Complement component 4 (C4) was one such protein validated by our immunofluorescence analysis (Fig. 6i, j). Assessment of protein expression in EVTs was conducted in the same manner. In this case, the proteomic signature of the coculture model consisted of 12 overexpressed (Supplementary Fig. 12) and 18 uniquely induced EVT proteins (Fig. 6k), 8 of which were identified as markers with unknown function in implantation and placentation.

Taken together, secretomics and protemics analysis of our model shows the presence of many known regulators of trophoblast function at the protein level but the data also suggest several proteins that may play previously unknown significant roles in EVT migration and invasion during implantation.

### Probing the role of maternal immune cells in implantation

**Fig. 7:**
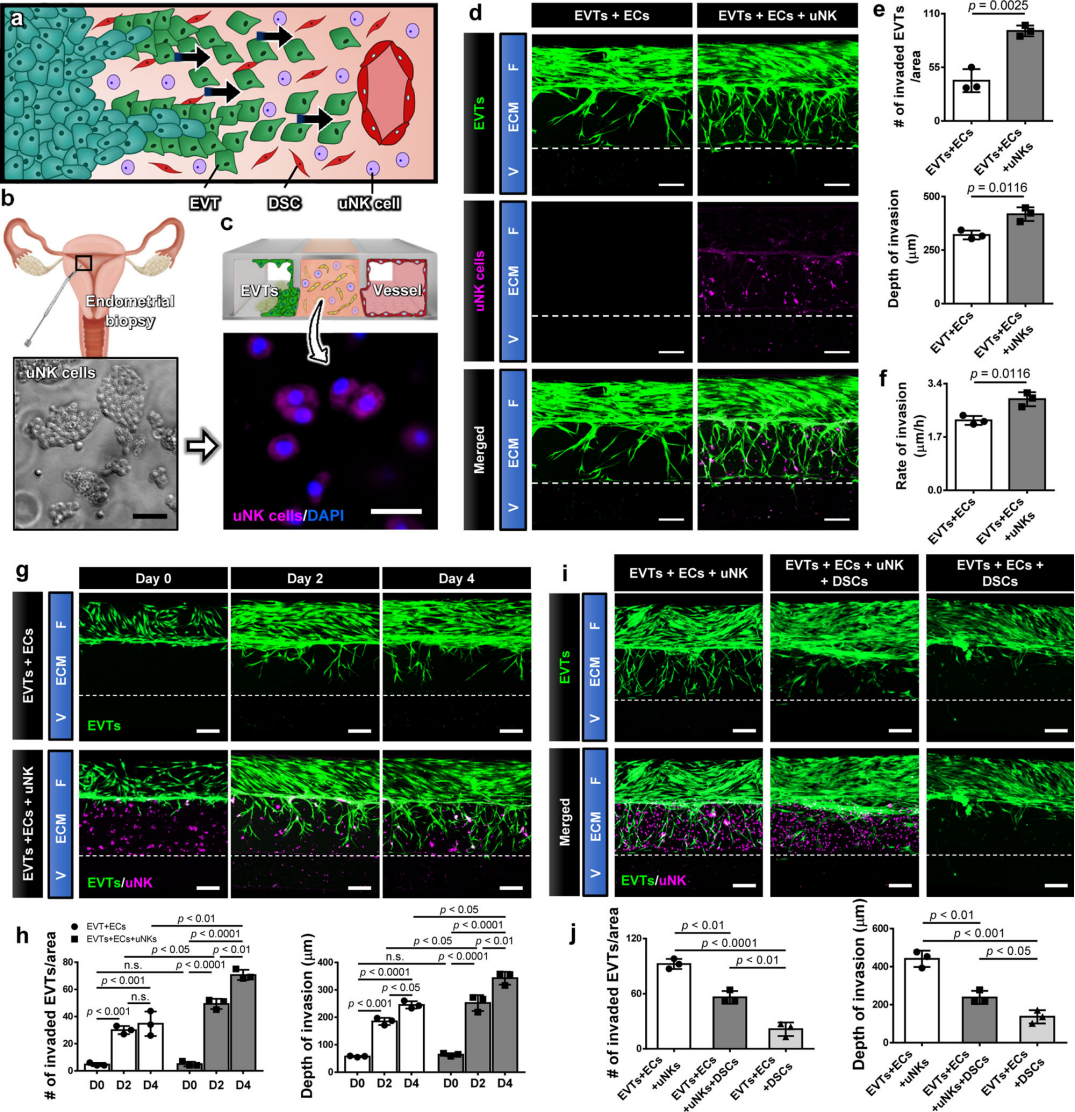
Immune regulation at EVT invasion by uNK cells. **a** Uterine NK cells are the most abundant maternal immune cells encountered by invading trophoblasts, and have been suggested to influence EVT invasion. **b** Isolation of primary human uNK cells from implantation window biopsies taken from non-pregnant endometrium. The representative images are from two independent experiments. Scale bar, 50 μm. **c** Encapsulation of uNK cells (magenta) in the hydrogel scaffold of the implantation-on-a-chip device. Blue shows nucelar staining. The representative images are from three independant experiments. Scale bar, 20 μm. **d** Promotion of EVT invasion due to uNK cells. Images were captured at Day 6. EVTs and uNK cells were pre-labeled with CellTracker Green and CellTracker Red, respectively. The representative images are from three independent experiments. Scale bars, 200 μm. **e**, **f** Quantification of the extent and rate of invasion. Two-sided *t*-test (*n* = 3 independent chips per group). **g** Time-lapse imaging of EVT (green) migration in the presence (bottom images) or absence (top images) of uNK cells (magenta). The representative images are from three independent experiments. Scale bars, 200 μm. **h** Quantification of EVT invasion over 4 days. Two-way ANOVA with Sidak’s multiple comparison test (*n* = 3 independent devices per group). **i**, **j** Analysis of altered EVT invasion due to the addition of DSCs. The representative images are from three independent experiments. Scale bars, 200 μm. One-way ANOVA with Tukey’s multiple comparison test (*n* = 3 independent devices per group). Dashed lines in **d**, **g**, and **i** indicate the boundary between the ECM hydrogel compartment and the vascular chamber. Data are presented as mean ± SD. *p* values are shown on the graphs. uNK cells: Uterine natural killer cells. Source data are provided as a Source Data file.

Maternal immune cells are highly abundant in the uterus, and accumulate prior to embryo implantation in preparation for pregnancy. Uterine natural killer (uNK) cells are the most abundant (50-70%) cell type in this population and are believed to play an important role in early pregnancy^33^. (Fig. 7a). Importantly, uNK cells increase in abundance during the portion of the menstrual cycle when implantation can take place. If conception occurs, this increase in uNK cell number lasts throughout EVT invasion^34^. It is poorly understood, however, how uNK cells modulate EVT function and contribute to physiological establishment of pregnancy, which remains an important outstanding question in reproductive biology and medicine^33^. Most studies examining this question utilize uNK cells isolated from first trimester pregnancy tissue. It should be noted, however, that the implanting embryo first encounters uNK cells that accumulate in the endometrium prior to pregnancy. Since uNK cells isolated from first trimester tissue have already been exposed to trophoblasts and the cellular microenvironment of the post-implantation uterus, it is highly questionable if they have the capacity to properly model the characteristics and function of uNK cells that participate in the initiation of EVT invasion^35^. Therefore, we aimed to explore this question in our implantation-on-a-chip using appropriately sourced human uNK cells. The focus of this preliminary work was to examine the effect of pre-implantation uNK cells on directional EVT migration.

For this study, we isolated primary human uNK cells unexposed to EVTs from endometrial biopsies taken from non-pregnant women during the implantation window (Fig. 7b). uNK cells were then incorporated into the ECM compartment of our device at the time of model construction and cultured with EVTs and uterine ECs (Fig. 7c). The addition of these cells exerted negligible effects on the growth and viability of the other cell populations but had significant functional consequences on EVTs by accentuating their invasive phenotype (Fig. 7d). Our quantitative analysis showed significant increases not only in the invading cell number and invasion depth/area (Fig. 7e) but also in the rate of EVT migration (Fig. 7f) in the presence of uNK cells. In this system, EVTs moved through the hydrogel more rapidly and reached the maternal vessel in 4 days, as opposed to 6 days in the absence of uNK cells (Fig. 7g).

While these results suggested the capacity of uNK cells to promote trophoblast invasion, a question remained whether the response of EVTs in this model accurately reflects the behavior of their in vivo counterparts subjected to a greater degree of cellular complexity in their native environment. To address this question, we repeated the same experiments in another system in which the hydrogel matrix contained both uNK cells and DSCs, the cell type shown in our study to inhibit EVT invasion (Fig. 5). Under the influence of the competing effects of these two maternal cell populations, EVTs showed substantially decreased ECM invasion compared to both the EVTs+ECs and EVTs+ECs+uNK models (Fig. 7i, j). Nevertheless, EVT invasion in this system was still greater than when the ECM hydrogel contained DSCs only without uNK cells (Fig. 7i, j). Based on these results, both the inhibitory effects of DSCs and the promotive effects of uNK cells are at play in the quadruple culture model but the contribution of DSCs appears to be more dominant, resulting in an overall decrease in EVT invasion.

## Discussion

The events surrounding embryo implantation and establishment of the placenta are critical for successful pregnancy and have a major impact on the well-being of mother and fetus, as well as their lifelong health. However, these processes are particularly hard to investigate due to difficulties in finding accurate parallels in animal models and oversimplified traditional cell culture models. Our study is significant in that it offers the promise of tackling this long-standing problem through the development of a primary human cell-based, realistic, and highly controllable microphysiological model of the maternal-fetal interface that can replicate the critical aspects of human implantation and early placentation.

It should be noted that related engineering approaches have been reported previously for in vitro studies of fetal trophoblasts in early pregnancy. For example, a microfluidic device has been described in a recent study that enables in vitro modeling and quantitative measurement of trophoblast migration in response to artificially generated cytokine gradients^36^. Researchers have also utilized bioprinting techniques to construct spatially defined 3D placental tissue analogs to investigate trophoblast invasion^37^. Representing a frontier of reproductive science, this nascent body of work has introduced engineered cell culture models as in vitro tools for the study of early human pregnancy. Moving beyond the proof-of-concept, however, major challenges emerge in realizing the full potential of these advanced model systems for implantation and placental research.

Among the most critical issues associated with existing in vitro technologies is a paucity of primary human cell models due to the well-known difficulty of sourcing and handling human materials of early pregnancy, especially those from the window of implantation. To address this problem, studies have demonstrated primary cultures derived from fetal and maternal tissues for the study of implantation and early placentation. However, the fact that such systems are usually established using post-implantation maternal tissue questions the validity of this approach because recent evidence suggests that the phenotype of various cell types at the maternal-fetal interface and the dynamics of their interactions could change after exposure to the embryo^35^. Another important issue is the failure or limited capacity of current models to recapitulate the biological complexity of the maternal tissue microenvironment that arises from its cellular heterogenity. This is particularly problematic given growing evidence pointing to the importance of cell-cell interactions in understanding the physiology of EVT invasion and SA remodeling^8^.

Our paper highlights advanced in vitro capabilities that make the implantation-on-a-chip model an attractive and enabling platform to tackle many of these challenges. Using a compartmentalized microdevice design, our microphysiological system is capable of mimicking the 3D microarchitecture and relative spatial arrangement of maternal and fetal tissues during implantation. Importantly, this model is constructed using primary cells isolated from the human placenta and uterus within appropriate time frames to accurately represent the maternal-fetal interface during the window of implantation. As demonstrated by our DSC and uNK cell studies, the multiplicity and individual addressability of culture chambers in the device provide a means to adjust the cellular complexity of our model at will and to precisely control and manipulate cells and their local microenvironment in a cell type-dependent manner. Thanks to the optical transparency of our elastomeric device, the implantation-on-a-chip also enables visualization, direct observation, and quantitative analysis of a sequence of complex, dynamic physiological events, such as directional migration of EVTs and their interaction with maternal vessels.

For more specific discussion, the key accomplishment of our study is to demonstrate the feasibility of recapitulating the migration of early trophoblasts towards maternal spiral arteries in a physiologically relevant human model system. We found that uterine ECs alone promote EVT invasion by upregulating the production of matrix metalloproteinases and that this maternal control is mediated by paracrine signaling factors (Figs. 3, 6). Prior studies on the effect of maternal vascular cells on trophoblast invasion have focused on the presence of adhesion molecules on ECs that enhance trophoblast migration^38^, and EC apoptosis^21^. Recent studies, however, have demonstrated that arterial ECs have a unique secretome that can stimulate trophoblast migration and recruit immune cells to initiate the process of vascular remodeling^39,40^. Evidence also shows that hypoxia, a known regulator of EVT migration, functions through altering the EC secretome, which in turn alters molecular markers of invasion in EVTs^41^. These data support findings from our device and strongly suggest that ECs play an active and integral role in regulating early trophoblast migration.

In addition, our study showed that EVTs in our model are proliferative and that ECs can maintain their proliferative capacity (Fig. 3j). This result is in contrast to previous in vitro reports of a lack of cell proliferation in EVTs isolated from primary first-trimester tissue and term placenta grown on Matrigel-coated plates^42,43^. In our study, cell characterization indicated that 60% of the isolated EVTs retained their ability to proliferate (Fig. 1h) and that a significant fraction (82%) of EVTs cultured in the device remained proliferative (Supplementary Fig. 1). To understand this difference, it is worth noting that in our protocol, the isolated EVTs are obtained as outgrowths from villous tissue, cultured in flasks, and passaged 3 times prior to device seeding. Based on recent evidence demonstrating the existence of biologically distinct subsets of EVTs in first trimester and term tissues^44^, it is possible that we may be selecting for a proliferative subpopulation of EVTs during this culture period. After seeding into the device, we speculate that the more physiological culture environment containing maternal ECs and 3D ECM may allow EVTs to retain their proliferative capacity. For more in-depth understanding, however, further studies are necessary to identify and investigate microenvironmental cues and endothelial factors that play an important role in regulating EVT proliferation.

Much of what is currently known about the function of maternal cells in early placentation comes from studies utilizing cells isolated from first-trimester pregnancies. A major shortcoming of this approach, however, is that the earliest stages of EVT invasion occurs in response to the uterine cellular environment created *prior* to the arrival of the embryo^45^. To resolve this issue and establish a more accurate representation of the native maternal environment encountered by invading EVTs, we obtained and incorporated *pre-implantation* stromal cells into our model. In this more physiological setting, the implantation-on-a-chip showed inhibition of EVT invasion due to DSCs (Fig. 5), providing evidence in support of their controversial role as gatekeepers whose function is to limit EVT migration. Another interesting finding is that EVT migration was recovered when DSCs were replaced with cells of different type or origin (Fig. 5f, g). Given that these other kinds of cells were embedded in the same type of ECM hydrogel at similar densities, it is unlikely that the inhibitory effects of DSCs are the result of the physical impediment to EVT migration by stromal cells distributed throughout the gel. Rather, these findings can be interpreted as organ-specificity of trophoblast-stromal interactions.

In vivo, the uterine cellular environment consists of several different cell types that produce a wide variety of soluble factors to interact with EVTs and control their invasion^8,12,18^. Significant progress in the study of these key regulatory factors has led to the identification of various growth factors, adhesion molecules, chemokines, cytokines, and matrix metalloproteinases^18^. Understanding the role of these factors is critical in elucidating the molecular mechanisms responsible for regulating the depth and timing of trophoblast invasion, which are key aspects in ensuring successful pregnancy. Conventional in vitro models of trophoblast invasion, however, support culture of only one or two cell types, often failing to mimic the heterogeneous cell populations of the uterus and their secretome that mediates the complex interplay between EVTs and the maternal environment^11^. Investigating how maternal biochemical factors regulate the behavior of EVTs is often challenged by the limitations of these models, which is best illustrated by conflicting results reported in recent literature. For example, in vitro models have found that factors secreted by DSCs promote trophoblast invasion, while similar studies have shown the opposite effect^8,23^. Recent work also suggests that the EVT secretome is altered by other maternal cell types^39^. Conventional culture systems lacking cellular complexity are thus limited in their ability to model synergistic interactions between maternal cells, making it challenging to identify master regulators of invasion.

The ability of the implantation-on-a-chip to engineer in vivo-like complex cellular environment of early implantation might allow us to overcome these pitfalls of conventional in vitro studies. In fact, proteomic analysis conducted in this model identified key molecular mediators involved in maternal-fetal interactions and their functional roles (Fig. 6). In particular, our study showed the importance of EC-EVT crosstalk in the regulation of EVT invasion by revealing significant changes in protein expression when EVTs and ECs were cultured together. Our data provide a collection of intracellular and secreted proteins that are expressed only in the presence of EVTs and ECs, or differentially regulated in coculture of ECs and EVTs maintained in a physiological 3D environment, some of which we suggest as regulators of EVT invasion whose function has not been investigated in the context of implantation and placental development (Fig. 6).

C4 is one of such proteins identified in this work. It has been established that C4 serves as a major effector of two out of three activation pathways within the complement system to play an essential role in immune response^46^. Functional implications of this protein in implantation, however, remains unknown. Consistent with emerging evidence suggesting the importance of proper complement regulation for the maintenance of normal pregnancy^47^, our finding warrants further investigation on the capacity of C4 and other components of the complement system to regulate trophoblast invasion during implantation. Another marker that is uniquely expressed in coculture of EVTs and ECs is Plexin-domain containing protein 2 (PLXDC2). Interestingly, this protein has been associated with the epithelial to mesenchymal transition (EMT) in human gastric cancer^48^. Although in a different organ, our result raises the possibility that PLXDC2 may be responsible for similar function in the placenta and play a role in regulating EMT, which is an important process required for the initiation of EVT migration^44^. Nucleobindin-1, a soluble factor upregulated in the coculture model, was recently shown to be essential for matrix metalloproteinase (MMP) trafficking, through which it is able to regulate matrix degradation and the invasive ability of breast cancer cells^49^. Considering that MMP activity and ECM degradation play a critical role in EVT invasion^27^, the proteomics data suggest a role for this protein in early pregnancy. We believe that these results and insights add value to an existing body of knowledge and also call for further investigation on the role of the identified proteins in early pregnancy. The demonstrated capabilities of our model may also play an instrumental role in future studies to understand the molecular basis of disordered placentation.

Pregnancy is an immunologically complex state requiring a balance between the ability to fight infection and tolerance towards fetal antigens. The maternal immune system plays an active role in the establishment and maintenance of pregnancy, and disturbances of immune cell populations have been associated with multiple adverse pregnancy outcomes, including recurrent pregnancy loss, fetal growth restriction, and preeclampsia^50^. As the predominant immune cell population in the maternal endometrium, uNK cells are enriched during the secretory phase of the menstrual cycle and remain highly abundant during time windows critical for embryo implantation and placentation^34^. Emerging evidence points to an active involvement of uNK cells in stromal cell decidualization, trophoblast invasion, and SA remodeling but studying their role in pregnancy is challenging for several reasons. First, the unique characteristics of uNK cells make it difficult to extrapolate their behavior from the studies of peripheral immune cells^51^. Second, research has shown significant interspecies differences in the development, phenotype, and function of uNK cells, which greatly reduces the translatability of in vivo findings in animal models to human conditions^52^. Most ongoing studies also rely on the use of uNK cells isolated from first trimester termination tissue after they have been exposed to EVTs, which may not properly reflect the behavior of uNK cells that exist during the earliest stages of trophoblast invasion. While limited, data have shown significant genetic, phenotypic, and functional differences between uNK cells isolated from pre-pregnancy uterine tissue and those obtained after pregnancy is established^35^. These findings suggest the importance of distinguishing uNK cell subtypes at different gestational ages in the study of early pregnancy. Finally, standardized functional readouts for the study of uNK cells do not exist since cytotoxicity assays, which are considered the gold standard for testing NK cell functionality, are irrelevant when applied to uNK cells, which do not display cytotoxic behavior^53^. Although animal and in vitro studies have shown the role of uNK cells and macrophages isolated *post implantation* in EVT invasion and SA remodeling, there is a scarcity of data on the function of these cells *prior* to trophoblast arrival^51^. Using pre-implantation primary human uNK cells, our study demonstrates that maternal uNK cells from secretory phase, non-pregnant endometrium have the functional capacity to alter the behavior of EVTs by increasing their ability to invade the surrounding matrix and migrate towards maternal vessels.

While our implantation-on-a-chip represents a significant advance from existing in vitro model systems, work remains to be done to address its limitations and further develop it into a more realistic and predictive model of the maternal-fetal interface. Specifically, efforts are needed to further increase the biological complexity of the model by incorporating maternal cell types that are absent in the current system. Considering that vascular smooth muscle cells can communicate with invading EVTs and play an important role in SA remodeling^4^, for example, attempts should be made to include these cells in our model and reflect its contributions to EVT invasion and vascular remodeling. As the second largest leukocyte population in the uterus, decidual macrophages are believed to influence biological processes involved in vascular remodeling and immune modulation. Building upon our preliminary study of uNK cells, future studies should also investigate the role of these cells and other types of maternal immune cells that appear at different stages of early pregnancy, which will allow us to enhance the physiological realism of our model.

On a related note, the absence of sex hormones in the soluble environment of our model is another important limitation of this study. Implantation causes a significant increase in the level of hormones including human chorionic gonadotrophin (hCG), progesterone (P_4_), and estradiol (E_2_) in maternal serum and placental tissue, many of which have been shown to affect the behavior of trophoblasts. Using HTR-8 cell culture, for instance, researchers have demonstrated the ability of hCG to regulate trophoblast invasion^54^. In a baboon model, studies have also shown that excessive E_2_ can reduce EVT invasion and SA remodeling^55^. Therefore, our model will benefit from efforts to incorporate these additional signaling cues and recapitulate their spatiotemporal distributions at the maternal-fetal interface. Given the small size and hydrophobicity of sex hormone molecules, this work may also require the modification of the surface chemistry of cell culture chambers made out of PDMS, which is known to absorb small hydrophobic molecules^56^.

In addition, work remains to be done to further investigate the role of the ECM microenvironment in implantation. For instance, based on a recent study describing differences in the stiffness of tissues involved in EVT invasion^15^, modifying the stiffness of the ECM hydrogel scaffold could be explored as a strategy to control the behavior of EVTs and maternal cells for the purposes of improving the physiological relevance of our model. Although beyond the scope of the present study, the question of how the hemodynamic environment of maternal blood vessels affects implantation might be of interest for future investigations. In the current study, our devices were cultured in static conditions to simulate occluded blood flow in the SAs due to trophoblast plugs formed in the first trimester, which has been suggested to promote EVT invasion in early pregnancy^57^. In the last first trimester, the plugs are known to dislodge or disintegrate to permit maternal blood flow to the placenta^2^ but it remains to be understood how the initiation of blood flow influences maternal-fetal interactions and how the premature onset of this process can lead to adverse pregnancy outcomes reported in the literature^58^). Perfusability of the vascular compartment of our device, combined with the ability to precisely control and manipulate the intravascular environment, will permit mechanistic investigation of these questions in future studies.

From a broader perspective, another important area of challenge and opportunity for future work is in vitro modeling of abnormal pregnancy. Many pregnancy complications are associated with abnormal EVT invasion and disordered placentation. We and others have shown that preexisting maternal conditions, such as obesity, and peri-conception exposures, such as those involved in IVF, can impact placentation and increase the risk of these adverse outcomes^59^. Despite significant research efforts, the rate of these adverse outcomes have not changed dramatically. The ability to identify interventions that prevent and reduce the risk of pregnancy complications rests upon a clear understanding of pathophysiology. However, many adverse outcomes that originate in early pregnancy are often not detected until fairly late in pregnancy, and a mechanistic link between implantation abnormalities and disorders of placentation has been challenging to establish. An exploration of how these conditions impact early pregnancy can be carried out using our model by incorporating maternal cells isolated from patients at risk for pregnancy complications. This will greatly aid in the understanding of these disorders and the development and testing of potential therapeutics.

In conclusion, our work provides an in vitro technology with the potential for significant impact on basic and translational research of human implantation and placentation. We believe that our implantation-on-a-chip technology offers the promise of fulfilling the urgent need for predictive preclinical models of human pregnancy and will make great contributions to advancing the frontier of research in human reproduction.

## Methods

### Compliance with ethical regulations

Research performed complies with ethical regulations set forward by the University of Pennsylvania. Human tissue was collected from the Penn Family Planning and Pregnancy Loss Center under an IRB that has been approved by the University of Pennsylvania (#827072), and endometrial biopsies were obtained through the Penn Fertility Clinic under IRB #828613. This went through a vigorous review process to ensure that tissue was being obtained ethically. All patients underwent an established protocol for obtaining informed consent which occurred after completion of a patient’s clinical visit by an independent research coordinator. The informed consent form was reviewed with the patient and signed by both the patient and the study coordinator. In accordance with current regulations regarding the use of human fetal tissue, no compensation was provided for obtaining first-trimester placental tissue, and $100 per biopsy was provided. Non-pregnant patients undergoing endometrial biopsies for cell collection were compensated $100 for their study visit as deemed appropriate by the IRB.

### Sample collection

EVTs were collected from placental villi obtained from first-trimester pregnancy terminations performed at the Penn Family and Pregnancy Loss Center (IRB#827072). Patients with preexisting medical conditions or any pregnancy complications were excluded from the study. Collected tissue was kept on ice and cell isolation was carried out within 1 h of obtaining tissue. Endometrial biopsies to obtain stromal cells and uNK cells were obtained from 15 healthy women between ages of 23 and 42 (mean age = 31, standard deviation = 6.02), with no significant medical history, not utilizing hormonal contraception and with regular menstrual cycles (between 25 and 35 days). (IRB #828613). Endometrial biopsies were obtained from subjects 8 days after a luteinizing hormone (LH) surge, which was detected in the urine by the Clearblue® Ovulation Test. Biopsies were obtained using a Pipelle® Endometrial Suction Curette (CooperSurgical).

### Primary cell isolation

Extravillous trophoblasts: Primary EVTs were isolated from first-trimester termination tissue based on an EVT-outgrowth based protocol established by Graham et al and well-documented by other investigators^60–63^. Briefly, villous tissue was finely minced and cultured at 37 °C in RPMI 1640 medium containing 20% charcoal-stripped fetal bovine serum (FBS, Gibco, Catalog # 10437). After villous fragment attachment, EVT outgrowth occurred, and cells were separated from tissue during washing and passaging of the cells. The isolated EVTs were then cultured in 6 well plates using RPMI 1640 medium containing 20% FBS and 1% penicillin (100 U/ml)/streptomycin (100 U/ml). The cultured EVTs were used for constructing the implantation-on-a-chip model after the first 3 passages. EVT identity of the cells used in our experiments were characterized by immunostaining of cytokeratin-7 and HLA-G.

Maternal cells: Endometrial tissue was washed in PBS and finely minced before being digested in a shaking 37 °C water bath for 15 min in digestion medium (sterile serum-free RPMI, 0.28WU/ml Liberase TM (Roche 05401127001), 30 µg/ml DNase (Roche 10104159001). A large-bore transfer pipet was used to pipet tissue up and down during digestion to breakdown tissue. Digested tissue was filtered through a 70 micron filter and peletted at 300 × g for 5 min. To remove red blood cell contamination, the cell pellet was resuspended in 5 ml ACK lysis buffer and incubated on ice for 5 mins. After lysis, ice cold PBS was added, and cells spun down at 300 × g for 5 min.

To obtain stromal cells (SCs), the cell pellet was resuspended in stromal cell culture medium (DMEM/F12, 10% charcoal-stripped FBS, 1% Penicillin/Streptomycin) before being plated on a 75 cm^2^ culture flask for 6–18 h to remove blood cells, debris and unattached endometrial epithelial cells. Once confluent, confirmation of SC purity was carried out using immunofluorescence, to show Vimentin^+^, Cytokertain 7^-^, CD31^-^, CD 45^-^. Decidualization was then carried out using 1 µM MPA, 10 nm E2, and 500 µm dibromo-cAMP (decidualization cocktail, DC), and confirmed through the production of IGFBP-1 and prolactin via qPCR. After 6 days of culture in DC, stromal cells were added to the device. Culture media used for device culture did not contain DC.

To obtain uterine NK cells, the cell pellet was resuspended in ice-cold MACS buffer (sterile PBS without Ca and Mg, 1% sterile filtered FBS, 2 mM EDTA), and stained for the following surface marker presentation: uNK cells—CD45^+^, CD3^-^, CD16^-^, CD56^bright^ before sorting on FACSAria Fusion cell sorters at the Children’s Hospital of Philadelphia Flow Cytometry Core. After sorting, uNK cells were eluted into uNK cell culture media (RPMI, 10% FBS, 1% Penicillin/Streptomycin, 15 ng/ml IL-15). All human tissue was obtained ethically under the supervision and approval of the University of Pennsylvania’s Institutional Review Board and with informed consent from the participants

### Cell Culture

The HTR-8/SV neo cell line (ATCC, catalog # CRL-3271) was cultured in RPMI-1640 medium containing 20% FBS and 1% penicillin (100 U/ml)/streptomycin (100 U/ml) solution. Human endometrial microvascular endothelial cells (HEMEC, ScienCell, catalog #7010), human lung microvascular endothelial cells (HMVEC-L, Lonza, catalog # CC-2527) and human brain microvascular endothelial cells (HBMVEC, AngioProteomie, catalog # CAP-0002) were cultured in Endothelial Cell Growth Medium MV2 (EGM-MV2, Promocell, catalog # C-22022). Normal human lung fibroblasts (NHLFs, Lonza, catalog # CC-2512) and human tracheal epithelial cells (TECs, PromoCell, catalog # C-12644) were maintained in Fibroblast Growth Medium (FGM)-2 (Lonza, catalog # CC-3132) and Airway Epithelial Cell Growth Medium (PromoCell, catalog # C-21060), respectively. Authentication details of cell lines used are provided as Supplementary Table 1.

### Production of implantation-on-chip model

Implantation-on-a-chip devices were fabricated using standard soft lithography protocols. PDMS (Sylgard, Dow Corning, catalog # SYLG184) base was mixed thoroughly with curing agent at a 10:1 ratio (w/w). This mixture was then poured over a SU-8 (SU-8 100, MicroChem Corp., MA) master containing microchannel features fabricated by standard photolithographic methods. The microfluidic device contained three parallel lanes defined by two microfabricated rails protruding from the device ceiling (Fig. 3a, 3b). After thermal curing at 65 °C overnight, the PDMS slab was peeled off from the mater, and a 1-mm biopsy punch was used to make throughholes at each end of 3 lanes that served as inlet and outlet access ports. The PDMS slab was then boned to another blank PDMS layer by a PDMS stamping method^64^ to create a fully enclosed system. To supply media to the fetal and vascular chambers during device culture, the top surface of the device was bonded to a PDMS slab containing four through holes with a diameter of 7 mm that were used as media reservoirs. To ensure fluidic connection, the holes were overlaid directly on the inlet and outlet access ports of the underlying cell culture chambers. The fully assembled microdevice was first sterilized using UV irradiation for 20 min. To strengthen the anchorage of ECM hydrogel scaffold to the channel surfaces, the microdevice was filled and incubated with a 2.0 mg/ml (w/v in 10 mM Tris-HCl buffer, pH 8.5) of poly(dopamine) (PDA, Sigma Aldrich) hydrochloride solution at 37 °C for 4h^65^. After removing PDA solution, the channel was rinsed with distilled deionized (DDI) water three times and dried at 65 °C overnight.

To make the ECM hydrogel precursor solution, rat tail collagen Type-1 (8 mg/ml, Corning, catalog # 354249) solution was prepared by mixing 10× PBS and 1 N NaOH to achieve physiological pH, and then Matrigel (10 mg/ml, Corning, catalog # 356231) was mixed with Col-1 solution at a ratio of 1:1 (v/v). To establish our model, we first injected the ECM precursor solution into the middle lane of an empty channel (Supplementary Movie 1) and incubated the device at 37 °C for 20 min to form an ECM hydrogel in the middle lane. To produce the devices containing DSCs, NHLFs, hTECs and/or uNK cells within the ECM hydrogel, each cell type was detached from the cell culture dishes, and the cell number was counted. An appropriate amount of cell suspension was transferred to an Eppendorf tube and centrifuged at 300 x g for 5 min at RT. The media was completely removed, leaving only the cell pellet in the tube. The cell pellet was re-suspended in an ECM precursor solution at a density of 2 million cells/ml and then injected into the middle lane of the device. After gelation, fibronectin solution (Corning, catalog # 354008, 0.1 mg/ml in EGM-2MV medium) was introduced into the vascular chamber only and treated for 2 h at 37 °C to facilitate the attachment of endothelial cells. Maternal ECs (10 million/ml) were injected into the vascular chamber and incubated for 1 h at 37 °C. During this step, the device was rotated manually by 90° every 10 minutes to ensure the attachment of the cells on all four surfaces of the vascular chamber. Once the cells established firm attachement, EVTs (8 million cells/ml) were introduced into the fetal chamber. The fetal chamber was not treated with ECM in order to promote the attchment of EVTs to the hydrogel scaffold. After EVT seeding, the device was tilted sideways so that EVTs could adhere to the exposed surface of ECM hydrogel. EVTs and uNK cells used in this study were fluorescently labeled by pre-incubating them with 5 μg/ml of CellTracker Green CMFDA (Thermo Fisher Scientific, Waltham, MA, USA, catalog # C7025) and 1 μg/ml of CellTracker Deep Red (Thermo Fisher Scientific, catalog # C34565) in DMEM supplemented 2% (v/v) FBS for 15 min at 37 °C, respectively.

For device culture, the reservoirs connected to the fetal and vascular chambers were filled with 2% FBS-supplemented RPMI and EGM-2MV media, respectively. During culture, devices were kept in cell culture incubators in normoxic conditions maintained at 5% CO_2_ and 37 °C. To simulate occluded blood flow to the placenta in the first trimester^11^ and focus on the investigation of chemotactic EVT migration, we prevented the flow of media in the cell culture chambers. This was achieved by filling the inlet and outlet reservoirs with equal volumes of media, which were replenished every other day throughout the culture period. During media exchange, extra precautions were taken to minimize the diruption of the culture environment due to unwanted gravity-driven flow in the chambers by emptying and refilling the inlet and outlet reservoirs within seconds in a rapidly alternating manner.

In studies involving culture of additional cell types described in Figs. 5 and 7, the media conditions remained unchanged, and the devices were still cultured with EVT and endothelial media in the fetal and vascular chambers, respectively. This strategy allowed us to keep the biochemical environment of our model the same across the compared experimental groups and to exclude the influence of confounding factors introduced by cell-type-specific growth factors and specialized media supplements (e.g., sex hormones, inflammatory cytokines) that have been shown to directly affect the growth and invasion of EVTs^55,66^. Proper behavior of the additional cell types under these conditions was confirmed by measuring their viability and the expression of cell-type-specific markers after 6 days of device culture (Supplementary Fig. 13).

### Immunofluorescence staining

After completion of cell culture experiments, media were removed from the reservoirs, and the channels were washed 3 times by flowing PBS. Cells cultured in flasks were similarly treated. Cells were fixed by introducing 4% paraformaldehyde (PFA, Thermo Scientific, catalog # AAJ19943K2) into the channels and incubated for 30 min at room temperature (RT), followed by 3 washes with PBS for all immunofluorescence experiments with the exception that HLA-G MEM-G/9 staining was carried out after cold acetone fixation at −20 degrees for 10 min. After cell permeabilization and blocking with 0.1% Triton-X and 1% bovine serum albumin (BSA, Sigma, catalog # 5217), respectively, the cells were incubated with primary antibodies against Cytokeratin-7 (EPR17078, Abcam, catalog # ab181598), CD-31 (P2B1, Abcam, catalog # ab24590), VE-cadherin (D87F2, Cell Signaling, catalog # 2500), Caspase-3 (Asp175, Cell Signaling, catalog # 9661), Prolactin (PRL02, Thermo Fisher, MA5-11998), Fibroblast surface protein (1B10, Abcam, catalog # ab11333), Ki67 (37C7-12, Abcam, catalog # ab245113) VCAM-1(6G9, Novus Biologicals, NBP1-47491), and Complement C4 (JM88-13, Thermo Fisher, catalog # MA5-32856) diluted at 1:200 in 1% BSA-containing PBS solution at 4 °C overnight. HLA-G (MEM-G/9, Biorad, catalog # MCA2044) antibody was used at 1:100 dilution. Subsequently, the cells were washed 3 times by flowing PBS and treated with appropriate secondary antibodies diluted at 1:200 in PBS containing 1% BSA for 2 h at room temperature. The cells were counterstained with DAPI, then washed with PBS three times. Detailed information and experimental conditions for the primary and secondary antibodies used in our study is provided in the Supplementary Table 2. Fluorescence imaging was performed using an inverted microscope with confocal capabilities (LSM 800, Carl Zeiss, Germany; 10 × 0.45 NA objectives).

### Quantification of EVT invasion

Fluorescence images of EVTs were obtained from the ECM matrix region between the maternal vascular and fetal compartments of the implantation-on-a-chip device. High magnification images were collected from three or four separate devices per each experimental group. EVT invasion was quantified by (i) the number of invading EVTs, (ii) the depth of EVT invasion, and (iii) the area of EVT invasion. To evaluate the cell number, EVTs in the ECM compartments were manually counted, and the average of total cell counts was plotted. For quantification of invasion depth, the sum of the vertical travel distances of all EVTs in a given field of view was divided by the total number of EVTs in the the same imaging area to calculate the average depth of invasion per cell number. Each data point in the invasion depth plots represents the mean of the avergae invasion depths measured from three randomly selected areas within a single device. Analysis of invasion area was achieved by using the Analyze pixels function of ImageJ (NIH) with appropriate thresholding to measure the area of ECM hydrogel covered by invading EVTs.

### Enzyme Linked Immunoabsorbent assay (ELISA)

To measure MMP-2 and MMP-9 secretion, enzyme-linked immunosorbent assay (ELISA) was conducted using effluent samples collected from implantation-on-a-chip devices maintained in two different culture conditions: EVT monoculture (EVT-mono) and EVT-endothelial coculture (CO). Three devices were prepared for each condition and cultured for 6 days with media exchange every other day. At Day 6, 400 µl of media was pipetted manually from each chamber through the open medium reserovoirs and combined into an eppendorf tube to generate a 800-µl media sample from each device. Cellular debris was removed by centrifugation at 3000 x g for 10 min at 4 °C, and the supernatant was transferred to a new tube. Three replicates of the spent media for each culture condition were prepared and stored at −80 °C until analysis. For this analysis, we used commerically available ELISA kits (Human MMP-2 DuoSet ELISA: R&D Systems, catalog # DY902, Human MMP-9 DuoSet ELISA: R&D Systems, catalog # DY911) following manufacturer’s protocols. The level of each MMP was calculated using standard curves constructed with recombinant proteins.

### Proliferation assay

To evaluate the effect of maternal endothelial cells on EVT proliferation, the number of Ki67-positive EVTs in EVT-mono and EVT-EC coculture devices was quantified and compared on days 1, 4 and 7. After immunofluorescence staining with Ki67 antibody, images of EVTs were obtained from the fetal and ECM compartments (1 mm^2^) of implantation-on-a-chip devices using a laser scanning confocal microscope (LSM 800, Carl Zeiss, Germany). For each experimental group, high-magnification images were randomly collected from three separate devices, and the number of Ki67-positive EVTs was automatically counted using the Analyze pixels function of ImageJ (NIH) and their mean values were plotted on a graph.

### Flow cytometry

To assess proliferating HLAG-positive cells from devices, media was removed, and the devices were washed twice with PBS. After removing residual PBS, the reservoirs of each channel were filled with 0.25% Trypsin and incubated at 37 °C for 15 minutes while pipetting every 5 min until the ECM hydrogel in the middle channel was completely dissolved. Cell suspensions were collected, centrifuged at 300 × *g* for 5 min, and then the supernatant was removed using a pipette, leaving only the cell pellet. Cells were then washed in MACS buffer (sterile PBS, 1% FBS, 2 mM EDTA), before staining with LIVE/DEAD Fixable Aqua Dead Cell Stain (Invitrogen, L34965) using 1 µl of reagent for 30 min in the dark. Antibody staining was performed at 4 °C for 1 h in the dark. The following conjugated antibodies, colors, clones and concentrations were used: PerCP-Cy5.5 CD45 (2D1, BioLegend, #368503, 1:100); Biotin HLA-G (MEM-G/9, Invitrogen, #MA1-19513, 1:50), Brilliant Violet 421™ Streptavidin (BioLegend, #405226,1:100). Following wash in MACS buffer, cell pellet was resuspended in PBS and fixed and permeabilized with 75% ethanol added dropwise, for 1 h at -20 °C to stain for APC Ki67 (16A8, BioLegend, 1:100).

### Measurement of tissue stiffness

After 6 days of device culture, the hydrogel constructs were harvested from the ECM compartment by carefully peeling apart the device layers while keeping the engineered tissues intact. The stiffness of the harvested hydrogel samples was measured by a compression test using a tensile-compressive tester (Tensilon RTC-1310A, Orientec Co.). For statistical anlaysis, five hydrogel samples were prepared for each experimental group shown in Supplementary Figs. 2 and 5b and kept in the media at 37 °C until tested.

### Viability assay

The viability of the cells encapsulated in the ECM compartment in the implantation-on-a-chip was investigated using LIVE/DEAD Cell Viability assay (Molecular Probes; Thermo Fisher Scientific, Inc. Catalog # L3224). On day 6 of device culture, a mixture of calcein AM and ethidium homodimer-1 (2 µM and 4 µM in culture media, respectively) was introduced into the fetal and vascular chambers, and incubated for 20 min at 37 °C in 5% CO_2_. The cells in the hydrogel were observed using an inverted fluorescence microscope (Axio Observer D1, Zeiss). For quantitative analysis, the ratio of live and dead cells was calculated by manually counting the number of cells stained with calcein AM and ethidium homodimer-1 in the images collected from the three devices in each experimental group.

### Measurement of vascular permeability

Endothelial barrier function was assessed by measuring transport of 70 kDa fluorescein isothiocyanatedextran (FITC-dextran; Sigma-Aldrich, catalog # 46945) across the maternal endothelium in our device. For this measurement, FITC-dextran (50 μg/ml in EGM2 media) solution was introduced into the vascular chamber and perfused for 30 min, after which media perfusate was collected from the fetal chamber. A microplate reader (M200, Tecan, Switzerland) was used to measure fluorescence intensity and determine the concentration of FITC-dextran in the collected samples. This calculation used a standards curve generated by measuring fluorescence intensity of 70 kDa FITC-dextran at known concentrations.

### Analysis of caspase activation

Apoptosis of the vascular endothelium was measured in our model after 6 days of culture by using cleaved Caspase-3 antibody (Asp175, Cell Signaling, catalog # 9661) (see above for immunostatining protocol). High magnification images of the endothelium were acquired on EC-mono and coculture devices, and the number of cells expressing caspase-3 and the mean fluorescence intensity (MFI) were measured in each region of interest for quantification.

### Proteomics analysis

Proteomics analysis was performed on conditioned media collected from the implantation-on-a-chip. We used eight implantation-on-a-chip devices for analysis of each culture condition. In a given device, 800 µl of media (400 µl from the fetal chamber and 400 µl from the vascular chamber) was sampled from the media reservoirs by using a pipette after 6 days of culture. A total of 1.6 ml of the spent media was collected from two devices and then combined into one tube, after which cell debris was removed by centrifugation. For each culture condition, four replicates were prepared from eight devices in this manner and frozen at -80 °C immediately until analysis. For the analysis of cell proteins, twelve implantation-on-a-chip devices for each culture condition were cultured for 6 days. After removing all media, the devices were washed twice with PBS. After removing residual PBS from both cell culture chamber with a pipette, the reservoirs of each channel were filled with 0.25% Trypsin and incubated at 37 °C for 15 min while pipetting every 5 min until the ECM hydrogel in the middle channel was completely dissolved. Cell suspensions were collected from three devices for one sample, centrifuged at 300 × *g* for 5 min, and then the supernatant was removed using a pipette, leaving only the cell pellet. The cell suspension isolated from the coculture device was subjected to a flow cytometry-based method (BD FACSAria) to isolate the EVT populations stained with Cell Tracker Green from the cell mixture. Isolated ECs and EVTs were washed twice with PBS to remove residual FBS and then centrifuged to collect only cell pellets. For each culture condition, four replicates were prepared from twelve devices and immediately frozen at -80 °C until analysis. Proteins were extracted from the spent media and the cell pellets using a chloroform/methanol extraction strategy derived from the Folch extraction. Cell pellets were resuspended in water, and 5 volumes of cold choloroform-methanol (2:1 [vol/vol]) was added to the samples before incubating on ice for 5 min and then vortexed for 1 min. Samples were then centrifuged at 15,000 × *g*. The interphase containing the proteins was collected and washed by adding 1 ml of cold methanol, after which samples were centrifuged at 15,000 × *g* for 10 min at 4 °C^67,68^. Isolated proteins were denatured, alkylated, digested with trypsin and desalted on a C18 SPE cartridge^68^. Peptides were analyzed by reverse phase LC–MS/MS using a Waters nanoEquityTM UPLC system (Millford) coupled with a Q-Exactive mass spectrometer (Thermo Scientific). Mass spectrometric raw data were analyzed using MaxQuant iBAQ label-free quantification (UniProtKB, downloaded in 2020) by searching against the Homo sapiens UniProt database. Proteins with at least one razor peptide were used. The subsequent data processing was performed in R v3.5.1 using the package RomicsProcessor v1.0.0, which is available on GitHub. This package that comprises several modules enabling to perform data assembly, data visualization, data clustering, allows to perform data subsetting, various normalization methods and general statistics. This omics-oriented package enables to process data in a non-destructive fashion by saving the original data frame, its associated metadata, the transformative steps of the data processing and the processed data in the same multi-layered type of R object named “romics_object”^69^. The data were log2 transformed and median normalized within each sample. Only proteins with at least 75% of valid quantitative values within one of the sample groups were conserved for quantification. To calculate certain statistics imputation of missing values was required, missing values were imputed using the method described by Tyanova et al^70^. and ANOVA and Student’s T-tests were used to determine significantly changed proteins (*p*-value < 0.05, estimated FDR = ~10%).

The numbers of quantified proteins from secretomics and proteomics analysis were 498 and 2,555, respectively. The average peptide number was 3.00 for the modulated proteins from secretomics analysis (Fig. 6e, f), 10.75 for the proteins enriched in EVT in coculture, and 12.67 for EC-enriched proteins in coculture. For EVT proteins uniquely expressed in coculture, the average number of peptides was 4.85 (Fig. 6k), and only 1 out of the 18 protein (Apolipoprotein C-III) had only one razor peptide. For unique EC proteins detected in coculture, the average number of peptides was 4.53 (Fig. 6g), and all proteins had more than 2 razor peptides.

PubMed and Google Scholar were used to conduct literature search on (i) proteins from secretomics analysis that were significantly changed in EVT and EC monoculture conditions in comparison to coculture and (ii) those from proteomics analysis that were uniquely expressed in coculture. The results of this search were used to investigate the known roles of the detected proteins in implantation and/or placentation and to identify proteins with previously unknown roles in implantation and/or placentation.

### Statistical analysis

The number of samples for each experiment was determined on the basis of a minimum of three independent devices for each experimental group (*n* = 3). Data were presented as mean ± SD. One-Way Anova and Two-sided t-test were performed on the data by using Prism to calculate *p* values and statistical significance.

### Reporting summary

Further information on research design is available in the Nature Research Reporting Summary linked to this article.

## Supplementary information

Supplementary Information

Description of Additional Supplementary Files

Supplementary Movie 1

Supplementary Movie 2

Reporting Summary

## Data Availability

The secretomics and proteomics data generated in this study have been deposited in the MassIVE database, a full member of the ProteomeXchange consortium under accession code MSV000086888 for the “secretome” and MSV000088677 for the “cellular proteome”. All other relevant data supporting the key findings of this study are available within the article and its Supplementary Information files or from the corresponding author upon reasonable request. Source data are provided with this paper.

## Source data

Source Data

## Code availibility

The MaxQuant results files and the R code were deposited on GitHub and Zenodo for the “secretomics data [10.5281/zenodo.5842089]” and “proteomics data [10.5281/zenodo.5842089 for the cellular proteome]”. Source data are provided with this paper.
