# Supplementary material for: A microphysiological model of human trophoblast invasion during implantation
Source: Nat Commun. 2022 Mar 15;13:1252. doi: 10.1038/s41467-022-28663-4 (PMC8924260; doi:10.1038/s41467-022-28663-4)
Supplement: Supplementary file 1 — Supplementary Information [file 41467_2022_28663_MOESM1_ESM.pdf]

Supplementary Information (SI) Guide for

A microphysiological model of human trophoblast invasion during implantation

[1]Ju Young Park, [2]Sneha Mani, [3]Geremy Clair, [3]Heather M. Olson, [3]Vanessa L. Paurus, [3]Charles K. Ansong, [1]Cassidy Blundell, [1]Rachel Young, [2]Jessica Kanter, [4]Scott Gordon, [1]Alex Y. Yi, [2]Monica Mainigi*, [1,5,6]Dan Dongeun Huh*

Supplementary Information includes:

Supplementary Figures 1-11

Supplementary Figure 1. Measurement of proliferative EVT population in the implantation-on-a-chip.

Supplementary Figure 2. Analysis of ECM stiffness in the implantation-on-a-chip.

Supplementary Figure 3. Comparison of invasive activity of primary EVTs and HTR8/SVneo cells on the 'implantation-on-a-chip' device.

Supplementary Figure 4. Maintenance of the decidualized phenotype of stromal cells in the implantation-on-a-chip.

Supplementary Figure 5. Analysis of ECM composition and stiffness in the presence of different cell types in the hydrogel compartment.

Supplementary Figure 6. Secretomics analysis of effluent samples collected from implantation-on-a-chip in four different culture conditions: i) EVT-mono, ii) EC-mono, iii) CO, and iv) TRI (one-way ANOVA p<0.05).

Supplementary Figure 7. Secretomics analysis of human proteins only. Z scores and the results of t tests of the different proteins identified in the different culture conditions: i) EVT-mono, ii) EC-mono, iii) CO, and iv) TRI (two-sided t-test).

Supplementary Figure 8. Heatmap depicting the human proteins significantly regulated in abundance in the different culture conditions: i) EVT-mono, ii) EC-mono, iii) CO, and iv) TRI

Supplementary Figure 9. List of proteins showing significant increase or decrease between EVT monoculture and coculture of EVT and EC (two-sided t-test).

Supplementary Figure 10. List of proteins showing significant increase or decrease between EC monoculture and coculture of EVT and EC (two-sided t-test).

Supplementary Figure 11. Fourteen significantly upregulated endothelial proteins in the coculture configuration of the implantation-on-a-chip.

Supplementary Figure 12. Twelve significantly upregulated EVT proteins in coculture configuration of the implantation-on-a-chip.

Supplementary Figure 13. Viability and marker expression of DSCs and additional cell types in the implantation-on-a-chip.

Supplementary Table 1. Cells and authentication methods.

Supplementary Table 2. Primary and secondary antibodies for immunofluorescence staining.

Supplementary Figure 1. Measurement of proliferative EVT population in the implantation-on-a-chip. (a) Immunofluorescence staining of HLA-G (purple) and Ki67 (yellow) in EVTs migrating in the ECM compartment at Day 6. The representative images are from three independent experiments. Scale bars, 50 μm. (b) Cells isolated from the device were flow sorted. Analysis of single, live, and CD45-negative cells stained with HLA-G and Ki67 showed that 82% of HLA-G^{+} cells were also Ki67 positive. FMO = fluorescence minus one control.

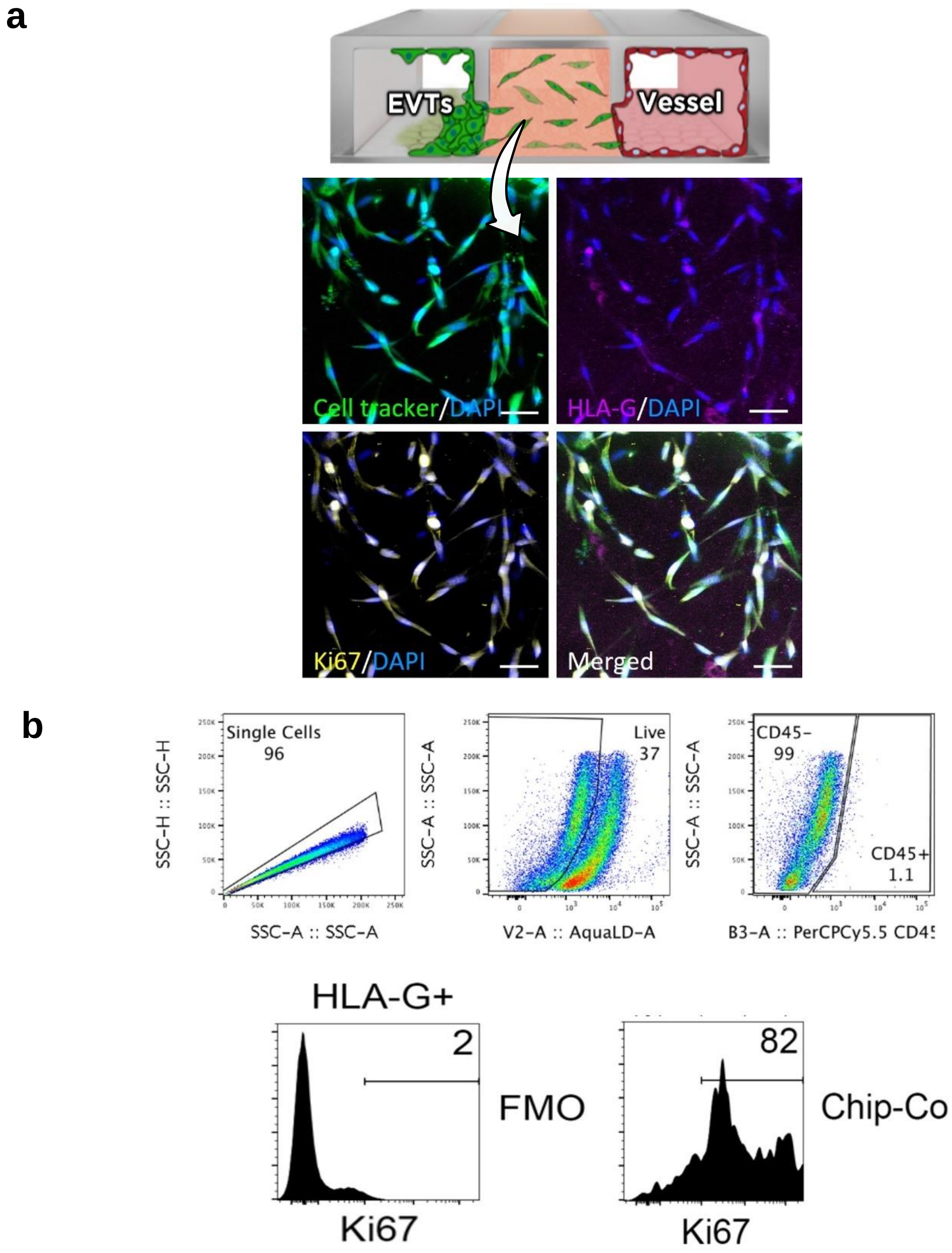

Supplementary Figure 2. Analysis of ECM stiffness in the implantation-on-a-chip. The plot shows the Young's modulus of ECM hydrogel created in the implantation-on-a-chip device prior to EVT invasion (left bar) and after 6 days of device culture (right bar). Data represent mean ± SD from five independent devices (n=5). P values by unpaired, two-sided t-test.

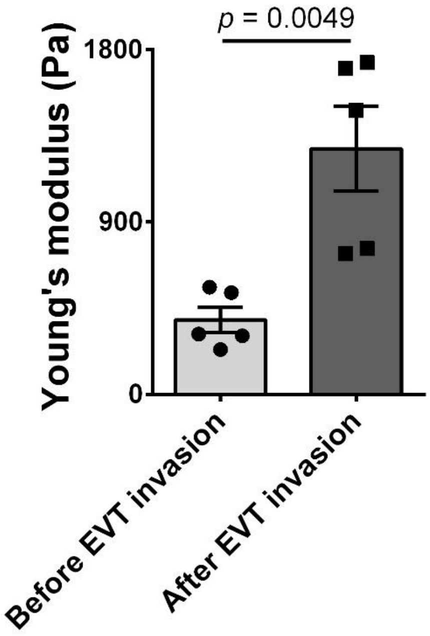

Supplementary Figure 3. Comparison of invasive activity of primary EVTs and HTR8/SVneo cells on the 'implantation-on-a-chip' device. (a) Invasion of primary EVTs in 2%FBS (first column) was compared to invasion of the HTR8-SVneo cell line in 2% FBS (second column) and 10% FBS (third column) at Day 1 (top panel) and Day 6 (bottom panel). The representative images are from three independent experiments (Scale bars, 200 µm). (b) Area invaded by the cells was quantified at day 6. Data represent mean ± SD from three independent devices (n=3). One-way ANOVA with Tukey's multiple comparison test. Dashed lines indicate the boundary between the ECM hydrogel and the vascular compartment.

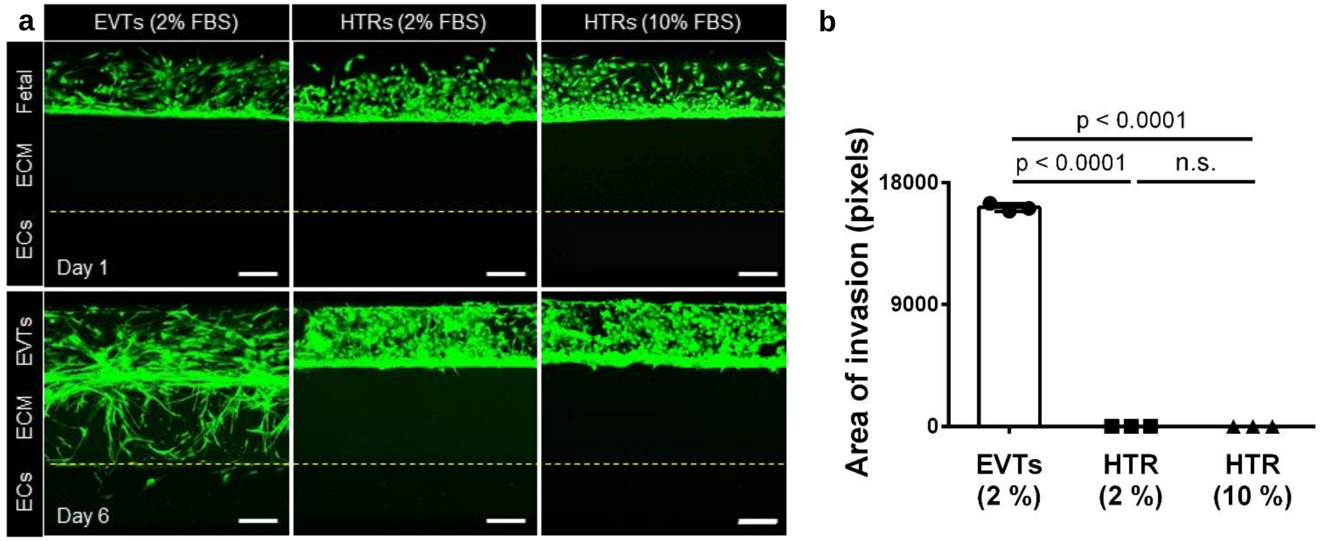

Supplementary Figure 4. Maintenance of the decidualized phenotype of stromal cells in the implantation-on-a-chip. Immunofluorescence staining of prolactin expression in DSCs cultured in the ECM compartment of the implantation-on-a-chip for 6 days without MPA, E_2, and cAMP. The representative images are from three independent experiments. The dashed lines show the boundary between the ECM compartment and side channels. F and V represent the fetal and vascular chambers, respectively. Scale bars, 100 μm.

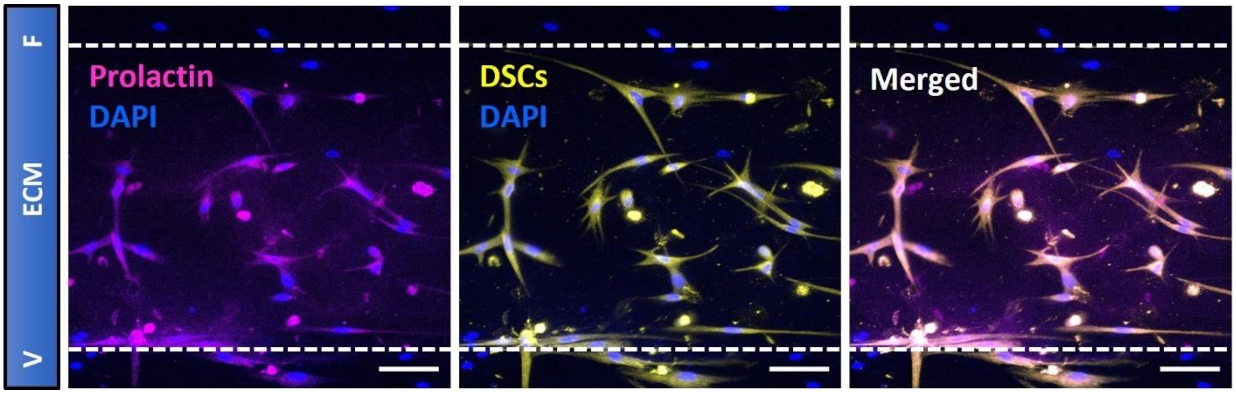

Supplementary Figure 5. Analysis of ECM composition and stiffness in the presence of different cell types in the hydrogel compartment. (a) Quantification of the immunofluorescence of collagen type I and laminin in DSC-, NHLF-, and hTEC-containing devices after 6 days of culture. Data represent mean ± SD from four indepdent devices (n=4). One-way ANOVA with Tukey's multiple comparison test. MFI stands for mean fluorescence intensity. (b) Young's modulus of the Matrigel-collagen I mixture hydrogel scaffold containing different cell types after 6 days of device culture. Data represent mean ± SD from five independent devices (n=5). One-way ANOVA with Tukey's multiple comparison test. n.s.; not significant

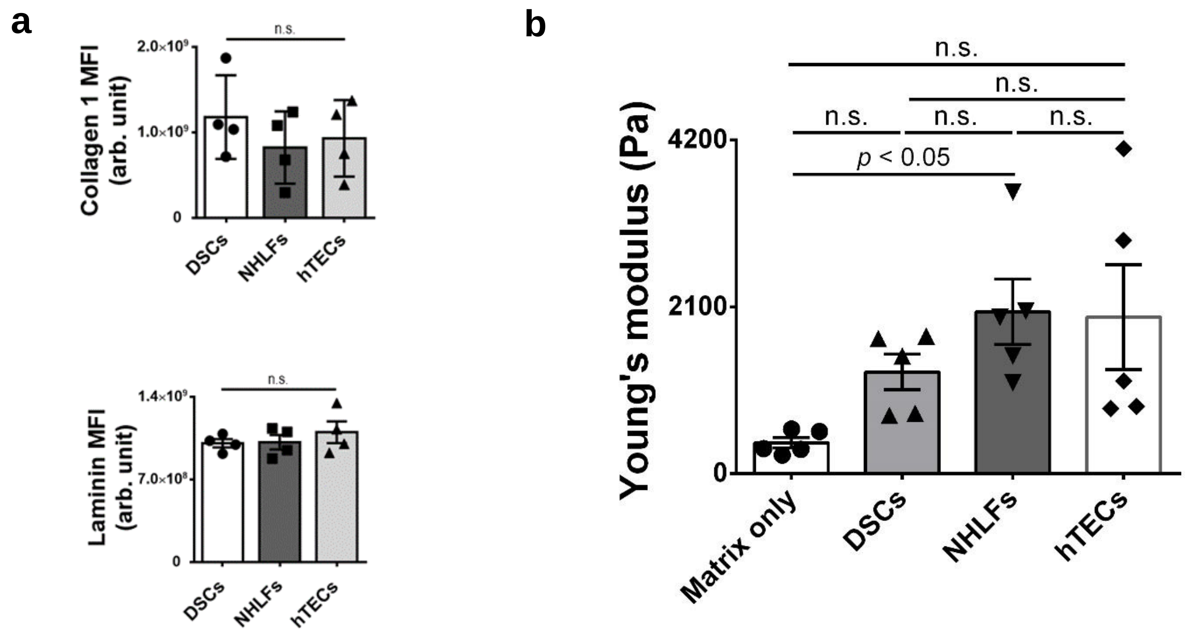

Supplementary Figure 6. Secretomics analysis of effluent samples collected from implantation-on-a-chip in four different culture conditions: i) EVT-mono, ii) EC-mono, iii) CO, and iv) TRI (one-way ANOVA $p<0.05$).

No.	Description from fasta	Human ID	Bovine ID	Uniquely human	EVT-mono				EC-mono				CO				TRI				P value
					1	2	3	4	1	2	3	4	1	2	3	4	1	2	3	4	
1	Proteasome subunit alpha type-2 (Fragment)	+	+	-																	0.003
2	Tubulin beta chain	+	-	+																	0
3	Thioredoxin reductase 1, cytoplasmic	+	+	-																	0.001
4	Collagen alpha-2(I) chain	+	-	+																	0
5	Proteasome subunit beta type-2	+	+	-																	0.028
6	Collagen alpha-1(VI) chain	+	-	+																	0.007
7	Cell adhesion molecule 1	+	+	-																	0.000
8	Histone H2A	+	+	-																	0.007
9	SERPIN domain-containing protein	-	+	-																	0.002
10	Receptor-type tyrosine-protein phosphatase S	+	+	-																	0
11	Peroxiredoxin-1 (Fragment)	+	+	-																	0.000
12	Translationally-controlled tumor protein	+	+	-																	0
13	Neurobeachin	+	+	-																	0.022
14	Microfibrillar-associated protein 5	-	+	-																	0
15	Carboxylic ester hydrolase	+	+	-																	0.002
16	Vitamin K-dependent protein C	-	+	-																	0.001
17	Glutathione S-transferase A1	-	+	-																	0.000
18	Pantetheinase	-	+	-																	0.011
19	Isocitrate dehydrogenase [NADP] cytoplasmic	+	+	-																	0.000
20	Legumain	-	+	-																	0.037
21	Tropomyosin alpha-4 chain	+	+	-																	0.002
22	Adiponectin B	-	+	-																	0.019
23	Fructose-bisphosphate aldolase	+	+	-																	0.003
24	Collagen alpha-1(IV) chain (Fragment)	+	+	-																	0
25	Serine protease HTRA1	+	+	-																	0
26	Complement component C7	-	+	-																	0
27	Ig-like domain-containing protein	-	+	-																	0.019
28	SPARC like 1	-	+	-																	0.001
29	Tenascin C	-	+	-																	0.001

30	Ig-like domain-containing protein	-	+	-	0.000
31	EMAP like 2	-	+	-	0.000
32	Adhesion G protein-coupled receptor E5	-	+	-	0.025
33	Collagen type V alpha 2 chain	-	+	-	0.020
34	Ig-like domain-containing protein	-	+	-	0.000
35	von Willebrand factor	-	+	-	0.012
36	Histone H4	+	+	-	0.021
37	Complement component C6	-	+	-	0
38	Fructose-bisphosphate al-dolase	-	+	-	0.003
39	Glutathione S-transferase P	-	+	-	0
40	Neogenin	+	+	-	0
41	Leukocyte elastase inhibitor	-	+	-	0.028
42	Uncharacterized protein	-	+	-	0.029
43	Protein disulfide-isomerase	+	+	-	0.000
44	Interleukin-1 receptor ac-cessory protein (Fragment)	+	+	-	0.002
45	Dystroglycan	-	+	-	0.031
46	Uncharacterized protein	-	+	-	0.009
47	Phosphoglucomutase-1	+	+	-	0.010
48	Fumarylacetoacetase	-	+	-	0.002
49	Lactoylglutathione lyase	+	+	-	0.003
50	Cellular repressor of E1A stimulated genes 1	-	+	-	0.043
51	Complement factor H	-	+	-	0.000
52	Uncharacterized protein	-	+	-	0.020
53	Plastin-2	+	+	-	0.001
54	Complement C8 alpha chain	-	+	-	0.036
55	Vasodilator-stimulated phosphoprotein	+	+	-	0.024
56	Cadherin-6	+	+	-	0.046
57	Adenosine kinase (Frag-ment)	+	+	-	0.025
58	Insulin-like growth factor-binding protein 6	-	+	-	0.018
59	Heart development protein with EGF like domains 1	-	+	-	0.002
60	ADP/ATP translocase 1	+	+	-	0
61	Ig-like domain-containing protein	-	+	-	0.032
62	Laminin subunit alpha 1	-	+	-	0.000
63	Ig-like domain-containing protein	-	+	-	0.034
64	Plexin domain-containing protein 2	+	+	-	0.010
65	Ferritin (Fragment)	+	+	-	0.011
66	Phosphatidylinositol-glycan-specific phospholipase D	-	+	-	0.002

67	Collagen type XV alpha 1 chain	-	+	-	[illegible]	[illegible]	[illegible]	[illegible]	[illegible]	[illegible]	[illegible]	[illegible]	[illegible]	[illegible]	[illegible]	[illegible]	[illegible]	[illegible]	[illegible]	[illegible]	0.006
68	Ectonucleotide pyrophos-phatase/phosphodiesterase family member 2	-	+	-	[illegible]	[illegible]	[illegible]	[illegible]	[illegible]	[illegible]	[illegible]	[illegible]	[illegible]	[illegible]	[illegible]	[illegible]	[illegible]	[illegible]	[illegible]	[illegible]	0.004
69	Proteoglycan 4	+	+	-	[illegible]	[illegible]	[illegible]	[illegible]	[illegible]	[illegible]	[illegible]	[illegible]	[illegible]	[illegible]	[illegible]	[illegible]	[illegible]	[illegible]	[illegible]	[illegible]	0.019
70	Ig-like domain-containing protein	-	+	-	[illegible]	[illegible]	[illegible]	[illegible]	[illegible]	[illegible]	[illegible]	[illegible]	[illegible]	[illegible]	[illegible]	[illegible]	[illegible]	[illegible]	[illegible]	[illegible]	0.007
71	Collagen alpha-2(I) chain	-	+	-	[illegible]	[illegible]	[illegible]	[illegible]	[illegible]	[illegible]	[illegible]	[illegible]	[illegible]	[illegible]	[illegible]	[illegible]	[illegible]	[illegible]	[illegible]	[illegible]	0.049
72	Four and a half LIM do-mains 1	-	+	-	[illegible]	[illegible]	[illegible]	[illegible]	[illegible]	[illegible]	[illegible]	[illegible]	[illegible]	[illegible]	[illegible]	[illegible]	[illegible]	[illegible]	[illegible]	[illegible]	0.029
73	KIAA1671	-	+	-	[illegible]	[illegible]	[illegible]	[illegible]	[illegible]	[illegible]	[illegible]	[illegible]	[illegible]	[illegible]	[illegible]	[illegible]	[illegible]	[illegible]	[illegible]	[illegible]	0.001
74	Spleen trypsin inhibitor I	-	+	-	[illegible]	[illegible]	[illegible]	[illegible]	[illegible]	[illegible]	[illegible]	[illegible]	[illegible]	[illegible]	[illegible]	[illegible]	[illegible]	[illegible]	[illegible]	[illegible]	0.001
75	Protein tyrosine phospha-tase receptor type G	-	+	-	[illegible]	[illegible]	[illegible]	[illegible]	[illegible]	[illegible]	[illegible]	[illegible]	[illegible]	[illegible]	[illegible]	[illegible]	[illegible]	[illegible]	[illegible]	[illegible]	0.000
76	Ig-like domain-containing protein	-	+	-	[illegible]	[illegible]	[illegible]	[illegible]	[illegible]	[illegible]	[illegible]	[illegible]	[illegible]	[illegible]	[illegible]	[illegible]	[illegible]	[illegible]	[illegible]	[illegible]	0
77	Uncharacterized protein	-	+	-	[illegible]	[illegible]	[illegible]	[illegible]	[illegible]	[illegible]	[illegible]	[illegible]	[illegible]	[illegible]	[illegible]	[illegible]	[illegible]	[illegible]	[illegible]	[illegible]	0.037
78	Tyrosine-protein kinase re-ceptor Tie-1	-	+	-	[illegible]	[illegible]	[illegible]	[illegible]	[illegible]	[illegible]	[illegible]	[illegible]	[illegible]	[illegible]	[illegible]	[illegible]	[illegible]	[illegible]	[illegible]	[illegible]	0.000
79	Transforming growth factor beta receptor 3	-	+	-	[illegible]	[illegible]	[illegible]	[illegible]	[illegible]	[illegible]	[illegible]	[illegible]	[illegible]	[illegible]	[illegible]	[illegible]	[illegible]	[illegible]	[illegible]	[illegible]	0.002
80	14-3-3 protein epsilon	+	+	-	[illegible]	[illegible]	[illegible]	[illegible]	[illegible]	[illegible]	[illegible]	[illegible]	[illegible]	[illegible]	[illegible]	[illegible]	[illegible]	[illegible]	[illegible]	[illegible]	0
81	Alpha-1,4 glucan phosphor-ylase	+	+	-	[illegible]	[illegible]	[illegible]	[illegible]	[illegible]	[illegible]	[illegible]	[illegible]	[illegible]	[illegible]	[illegible]	[illegible]	[illegible]	[illegible]	[illegible]	[illegible]	0.024
82	Glycoprotein 2	-	+	-	[illegible]	[illegible]	[illegible]	[illegible]	[illegible]	[illegible]	[illegible]	[illegible]	[illegible]	[illegible]	[illegible]	[illegible]	[illegible]	[illegible]	[illegible]	[illegible]	0.004
83	Tubulin alpha-4A chain	+	+	-	[illegible]	[illegible]	[illegible]	[illegible]	[illegible]	[illegible]	[illegible]	[illegible]	[illegible]	[illegible]	[illegible]	[illegible]	[illegible]	[illegible]	[illegible]	[illegible]	0
84	Cell adhesion molecule L1 like	-	+	-	[illegible]	[illegible]	[illegible]	[illegible]	[illegible]	[illegible]	[illegible]	[illegible]	[illegible]	[illegible]	[illegible]	[illegible]	[illegible]	[illegible]	[illegible]	[illegible]	0.008
85	Complement factor proper-din	-	+	-	[illegible]	[illegible]	[illegible]	[illegible]	[illegible]	[illegible]	[illegible]	[illegible]	[illegible]	[illegible]	[illegible]	[illegible]	[illegible]	[illegible]	[illegible]	[illegible]	0.006
86	Uncharacterized protein	-	+	-	[illegible]	[illegible]	[illegible]	[illegible]	[illegible]	[illegible]	[illegible]	[illegible]	[illegible]	[illegible]	[illegible]	[illegible]	[illegible]	[illegible]	[illegible]	[illegible]	0.043
87	Ig-like domain-containing protein	-	+	-	[illegible]	[illegible]	[illegible]	[illegible]	[illegible]	[illegible]	[illegible]	[illegible]	[illegible]	[illegible]	[illegible]	[illegible]	[illegible]	[illegible]	[illegible]	[illegible]	0.015
88	Uncharacterized protein	-	+	-	[illegible]	[illegible]	[illegible]	[illegible]	[illegible]	[illegible]	[illegible]	[illegible]	[illegible]	[illegible]	[illegible]	[illegible]	[illegible]	[illegible]	[illegible]	[illegible]	0
89	Cathepsin D	-	+	-	[illegible]	[illegible]	[illegible]	[illegible]	[illegible]	[illegible]	[illegible]	[illegible]	[illegible]	[illegible]	[illegible]	[illegible]	[illegible]	[illegible]	[illegible]	[illegible]	0.012
90	Polyubiquitin-B (Fragment)	+	+	-	[illegible]	[illegible]	[illegible]	[illegible]	[illegible]	[illegible]	[illegible]	[illegible]	[illegible]	[illegible]	[illegible]	[illegible]	[illegible]	[illegible]	[illegible]	[illegible]	0.002
91	Histone H3	+	+	-	[illegible]	[illegible]	[illegible]	[illegible]	[illegible]	[illegible]	[illegible]	[illegible]	[illegible]	[illegible]	[illegible]	[illegible]	[illegible]	[illegible]	[illegible]	[illegible]	0.014
92	C-type mannose receptor 2	+	+	-	[illegible]	[illegible]	[illegible]	[illegible]	[illegible]	[illegible]	[illegible]	[illegible]	[illegible]	[illegible]	[illegible]	[illegible]	[illegible]	[illegible]	[illegible]	[illegible]	0.000
93	Dickkopf-related protein 3	+	+	-	[illegible]	[illegible]	[illegible]	[illegible]	[illegible]	[illegible]	[illegible]	[illegible]	[illegible]	[illegible]	[illegible]	[illegible]	[illegible]	[illegible]	[illegible]	[illegible]	0.000
94	Extracellular matrix protein 1	-	+	-	[illegible]	[illegible]	[illegible]	[illegible]	[illegible]	[illegible]	[illegible]	[illegible]	[illegible]	[illegible]	[illegible]	[illegible]	[illegible]	[illegible]	[illegible]	[illegible]	0.038
95	L-lactate dehydrogenase	+	+	-	[illegible]	[illegible]	[illegible]	[illegible]	[illegible]	[illegible]	[illegible]	[illegible]	[illegible]	[illegible]	[illegible]	[illegible]	[illegible]	[illegible]	[illegible]	[illegible]	0.003
96	Ig-like domain-containing protein	-	+	-	[illegible]	[illegible]	[illegible]	[illegible]	[illegible]	[illegible]	[illegible]	[illegible]	[illegible]	[illegible]	[illegible]	[illegible]	[illegible]	[illegible]	[illegible]	[illegible]	0.005
97	Thyroglobulin	-	+	-	[illegible]	[illegible]	[illegible]	[illegible]	[illegible]	[illegible]	[illegible]	[illegible]	[illegible]	[illegible]	[illegible]	[illegible]	[illegible]	[illegible]	[illegible]	[illegible]	0.013
98	Epidermal growth factor re-ceptor	+	+	-	[illegible]	[illegible]	[illegible]	[illegible]	[illegible]	[illegible]	[illegible]	[illegible]	[illegible]	[illegible]	[illegible]	[illegible]	[illegible]	[illegible]	[illegible]	[illegible]	0.031
99	Contactin-1	+	+	-	[illegible]	[illegible]	[illegible]	[illegible]	[illegible]	[illegible]	[illegible]	[illegible]	[illegible]	[illegible]	[illegible]	[illegible]	[illegible]	[illegible]	[illegible]	[illegible]	0.047
100	Complement C1s subcom-ponent	-	+	-	[illegible]	[illegible]	[illegible]	[illegible]	[illegible]	[illegible]	[illegible]	[illegible]	[illegible]	[illegible]	[illegible]	[illegible]	[illegible]	[illegible]	[illegible]	[illegible]	0.001
101	Vitamin K-dependent pro-tein Z	-	+	-	[illegible]	[illegible]	[illegible]	[illegible]	[illegible]	[illegible]	[illegible]	[illegible]	[illegible]	[illegible]	[illegible]	[illegible]	[illegible]	[illegible]	[illegible]	[illegible]	0.001
102	Uncharacterized protein	-	+	-	[illegible]	[illegible]	[illegible]	[illegible]	[illegible]	[illegible]	[illegible]	[illegible]	[illegible]	[illegible]	[illegible]	[illegible]	[illegible]	[illegible]	[illegible]	[illegible]	0.002

103	Collectin-11	-	+	-	0.012
104	Protein kinase C-binding protein NELL2 (Fragment)	+	+	-	0.001
105	Uncharacterized protein	-	+	-	0.023
106	Aminopeptidase	-	+	-	0.001
107	Haptoglobin	-	+	-	0.003
108	Histone H2B	+	+	-	0.026
109	Alpha-actinin-1	+	+	-	0.018
110	Ig-like domain-containing protein	-	+	-	0.000
111	SH3KBP1-binding protein 1	-	+	-	0
112	Serglycin	-	+	-	0.027
113	Neural cell adhesion mole-cule 1	+	+	-	0.027
114	Ig-like domain-containing protein	-	+	-	0.004
115	Destrin	+	+	-	0
116	Mannan binding lectin ser-ine peptidase 1	-	+	-	0.017
117	Neuropilin	-	+	-	0.048
118	PKHD1 like 1	-	+	-	0.041
119	Prostaglandin-H2 D-isomer-ase	-	+	-	0.011
120	Complement C1r	-	+	-	0.000
121	Cadherin-13	+	+	-	0.005
122	Prosaposin	-	+	-	0.000
123	Ig-like domain-containing protein	-	+	-	0.032
124	EGF containing fibulin ex-tracellular matrix protein 1	-	+	-	0.001
125	Ig-like domain-containing protein	-	+	-	0.000
126	Transmembrane protein 132C	-	+	-	0.001
127	Talin 1	-	+	-	0.050
128	Regucalcin	-	+	-	0.011
129	Argininosuccinate synthase	-	+	-	0
130	EGF-containing fibulin-like extracellular matrix protein2	+	+	-	0.008
131	Uncharacterized protein	-	+	-	0
132	Rab GDP dissociation in-hibitor beta	+	+	-	0.001
133	Solute carrier family 3 member 2	-	+	-	0.047
134	Plexin B2	-	+	-	0.005
135	VWFA domain-containing protein	-	+	-	0.012
136	Lipocln_cytosolic_FA-bd_dom domain-containing protein	-	+	-	0.012
137	Ig-like domain-containing protein	-	+	-	0.019
138	Lipocln_cytosolic_FA-bd_dom domain-containing protein	-	+	-	0.022

139	Ig-like domain-containing protein	-	+	-	[illegible]	[illegible]	[illegible]	[illegible]	[illegible]	[illegible]	[illegible]	[illegible]	[illegible]	[illegible]	[illegible]	[illegible]	[illegible]	[illegible]	[illegible]	[illegible]	0.008
140	Cadherin-1	-	+	-	[illegible]	[illegible]	[illegible]	[illegible]	[illegible]	[illegible]	[illegible]	[illegible]	[illegible]	[illegible]	[illegible]	[illegible]	[illegible]	[illegible]	[illegible]	[illegible]	0.019
141	Retinol-binding protein 4	-	+	-	[illegible]	[illegible]	[illegible]	[illegible]	[illegible]	[illegible]	[illegible]	[illegible]	[illegible]	[illegible]	[illegible]	[illegible]	[illegible]	[illegible]	[illegible]	[illegible]	0.033
142	Lysosomal-associated membrane protein 2	-	+	-	[illegible]	[illegible]	[illegible]	[illegible]	[illegible]	[illegible]	[illegible]	[illegible]	[illegible]	[illegible]	[illegible]	[illegible]	[illegible]	[illegible]	[illegible]	[illegible]	0.022
143	Follistatin-related protein 1	+	+	-	[illegible]	[illegible]	[illegible]	[illegible]	[illegible]	[illegible]	[illegible]	[illegible]	[illegible]	[illegible]	[illegible]	[illegible]	[illegible]	[illegible]	[illegible]	[illegible]	0.003
144	Annexin A5	+	+	-	[illegible]	[illegible]	[illegible]	[illegible]	[illegible]	[illegible]	[illegible]	[illegible]	[illegible]	[illegible]	[illegible]	[illegible]	[illegible]	[illegible]	[illegible]	[illegible]	0.001
145	Beta-2-microglobulin	-	+	-	[illegible]	[illegible]	[illegible]	[illegible]	[illegible]	[illegible]	[illegible]	[illegible]	[illegible]	[illegible]	[illegible]	[illegible]	[illegible]	[illegible]	[illegible]	[illegible]	0.001
146	Acetyl-CoA acetyltransfer-ase 2	-	+	-	[illegible]	[illegible]	[illegible]	[illegible]	[illegible]	[illegible]	[illegible]	[illegible]	[illegible]	[illegible]	[illegible]	[illegible]	[illegible]	[illegible]	[illegible]	[illegible]	0
147	Biotinidase	-	+	-	[illegible]	[illegible]	[illegible]	[illegible]	[illegible]	[illegible]	[illegible]	[illegible]	[illegible]	[illegible]	[illegible]	[illegible]	[illegible]	[illegible]	[illegible]	[illegible]	0.015
148	Malic enzyme	-	+	-	[illegible]	[illegible]	[illegible]	[illegible]	[illegible]	[illegible]	[illegible]	[illegible]	[illegible]	[illegible]	[illegible]	[illegible]	[illegible]	[illegible]	[illegible]	[illegible]	0.000
149	Ig-like domain-containing protein	-	+	-	[illegible]	[illegible]	[illegible]	[illegible]	[illegible]	[illegible]	[illegible]	[illegible]	[illegible]	[illegible]	[illegible]	[illegible]	[illegible]	[illegible]	[illegible]	[illegible]	0
150	Laminin subunit beta 1	-	+	-	[illegible]	[illegible]	[illegible]	[illegible]	[illegible]	[illegible]	[illegible]	[illegible]	[illegible]	[illegible]	[illegible]	[illegible]	[illegible]	[illegible]	[illegible]	[illegible]	0
151	Calsyntenin-1 (Fragment)	+	+	-	[illegible]	[illegible]	[illegible]	[illegible]	[illegible]	[illegible]	[illegible]	[illegible]	[illegible]	[illegible]	[illegible]	[illegible]	[illegible]	[illegible]	[illegible]	[illegible]	0.008
152	Ig-like domain-containing protein	-	+	-	[illegible]	[illegible]	[illegible]	[illegible]	[illegible]	[illegible]	[illegible]	[illegible]	[illegible]	[illegible]	[illegible]	[illegible]	[illegible]	[illegible]	[illegible]	[illegible]	0.001
153	Platelet-derived growth fac-tor receptor beta	-	+	-	[illegible]	[illegible]	[illegible]	[illegible]	[illegible]	[illegible]	[illegible]	[illegible]	[illegible]	[illegible]	[illegible]	[illegible]	[illegible]	[illegible]	[illegible]	[illegible]	0.034
154	SPARC	+	+	-	[illegible]	[illegible]	[illegible]	[illegible]	[illegible]	[illegible]	[illegible]	[illegible]	[illegible]	[illegible]	[illegible]	[illegible]	[illegible]	[illegible]	[illegible]	[illegible]	0.001
155	Homogentisate 1,2-dioxy-genase	+	+	-	[illegible]	[illegible]	[illegible]	[illegible]	[illegible]	[illegible]	[illegible]	[illegible]	[illegible]	[illegible]	[illegible]	[illegible]	[illegible]	[illegible]	[illegible]	[illegible]	0.004
156	Sulfhydryl oxidase	-	+	-	[illegible]	[illegible]	[illegible]	[illegible]	[illegible]	[illegible]	[illegible]	[illegible]	[illegible]	[illegible]	[illegible]	[illegible]	[illegible]	[illegible]	[illegible]	[illegible]	0
157	Uncharacterized protein	-	+	-	[illegible]	[illegible]	[illegible]	[illegible]	[illegible]	[illegible]	[illegible]	[illegible]	[illegible]	[illegible]	[illegible]	[illegible]	[illegible]	[illegible]	[illegible]	[illegible]	0.011
158	Voltage-dependent calcium channel subunit alpha-2/delta-1	+	+	-	[illegible]	[illegible]	[illegible]	[illegible]	[illegible]	[illegible]	[illegible]	[illegible]	[illegible]	[illegible]	[illegible]	[illegible]	[illegible]	[illegible]	[illegible]	[illegible]	0.003
159	Xaa-Pro dipeptidase (Frag-ment)	+	+	-	[illegible]	[illegible]	[illegible]	[illegible]	[illegible]	[illegible]	[illegible]	[illegible]	[illegible]	[illegible]	[illegible]	[illegible]	[illegible]	[illegible]	[illegible]	[illegible]	0.025
160	Ceruloplasmin	-	+	-	[illegible]	[illegible]	[illegible]	[illegible]	[illegible]	[illegible]	[illegible]	[illegible]	[illegible]	[illegible]	[illegible]	[illegible]	[illegible]	[illegible]	[illegible]	[illegible]	0.022
161	Integrin alpha-2	+	+	-	[illegible]	[illegible]	[illegible]	[illegible]	[illegible]	[illegible]	[illegible]	[illegible]	[illegible]	[illegible]	[illegible]	[illegible]	[illegible]	[illegible]	[illegible]	[illegible]	0.036
162	Ig-like domain-containing protein	-	+	-	[illegible]	[illegible]	[illegible]	[illegible]	[illegible]	[illegible]	[illegible]	[illegible]	[illegible]	[illegible]	[illegible]	[illegible]	[illegible]	[illegible]	[illegible]	[illegible]	0.002
163	Complement C2	-	+	-	[illegible]	[illegible]	[illegible]	[illegible]	[illegible]	[illegible]	[illegible]	[illegible]	[illegible]	[illegible]	[illegible]	[illegible]	[illegible]	[illegible]	[illegible]	[illegible]	0
164	6-phosphogluconate dehy-drogenase, decarboxylating (Fragment)	+	+	-	[illegible]	[illegible]	[illegible]	[illegible]	[illegible]	[illegible]	[illegible]	[illegible]	[illegible]	[illegible]	[illegible]	[illegible]	[illegible]	[illegible]	[illegible]	[illegible]	0.037
165	Betaine--homocysteine S-methyltransferase 1	+	+	-	[illegible]	[illegible]	[illegible]	[illegible]	[illegible]	[illegible]	[illegible]	[illegible]	[illegible]	[illegible]	[illegible]	[illegible]	[illegible]	[illegible]	[illegible]	[illegible]	0.004
166	Amine oxidase	-	+	-	[illegible]	[illegible]	[illegible]	[illegible]	[illegible]	[illegible]	[illegible]	[illegible]	[illegible]	[illegible]	[illegible]	[illegible]	[illegible]	[illegible]	[illegible]	[illegible]	0.001
167	Nucleobindin-1 (Fragment)	+	+	-	[illegible]	[illegible]	[illegible]	[illegible]	[illegible]	[illegible]	[illegible]	[illegible]	[illegible]	[illegible]	[illegible]	[illegible]	[illegible]	[illegible]	[illegible]	[illegible]	0.017
168	Metalloproteinase inhibitor 1	-	+	-	[illegible]	[illegible]	[illegible]	[illegible]	[illegible]	[illegible]	[illegible]	[illegible]	[illegible]	[illegible]	[illegible]	[illegible]	[illegible]	[illegible]	[illegible]	[illegible]	0.031
169	Phospholipase A1 member A	-	+	-	[illegible]	[illegible]	[illegible]	[illegible]	[illegible]	[illegible]	[illegible]	[illegible]	[illegible]	[illegible]	[illegible]	[illegible]	[illegible]	[illegible]	[illegible]	[illegible]	0.031
170	Myosin light polypeptide 6	+	+	-	[illegible]	[illegible]	[illegible]	[illegible]	[illegible]	[illegible]	[illegible]	[illegible]	[illegible]	[illegible]	[illegible]	[illegible]	[illegible]	[illegible]	[illegible]	[illegible]	0.042
171	Olfactomedin-like protein 3	+	+	-	[illegible]	[illegible]	[illegible]	[illegible]	[illegible]	[illegible]	[illegible]	[illegible]	[illegible]	[illegible]	[illegible]	[illegible]	[illegible]	[illegible]	[illegible]	[illegible]	0.007
172	3-hydroxyanthranilate 3,4-dioxygenase	-	+	-	[illegible]	[illegible]	[illegible]	[illegible]	[illegible]	[illegible]	[illegible]	[illegible]	[illegible]	[illegible]	[illegible]	[illegible]	[illegible]	[illegible]	[illegible]	[illegible]	0.003
173	Coagulation factor X	-	+	-	[illegible]	[illegible]	[illegible]	[illegible]	[illegible]	[illegible]	[illegible]	[illegible]	[illegible]	[illegible]	[illegible]	[illegible]	[illegible]	[illegible]	[illegible]	[illegible]	0.010
174	Malate dehydrogenase, cy-toplasmic	+	+	-	[illegible]	[illegible]	[illegible]	[illegible]	[illegible]	[illegible]	[illegible]	[illegible]	[illegible]	[illegible]	[illegible]	[illegible]	[illegible]	[illegible]	[illegible]	[illegible]	0.006

175	Triosephosphate isomerase	-	+	-	0.001
176	Thrombospondin-4	-	+	-	0.006
177	Tubulin alpha-1A chain	+	+	-	0.008
178	Rho GDP-dissociation inhibitor 1	-	+	-	0.001
179	Angiotensin-converting enzyme	-	+	-	0
180	Zinc-alpha-2-glycoprotein	-	+	-	0.041
181	Insulin-like growth factor II	+	+	-	0.024
182	IL6ST nirs variant 3	+	+	-	0.019
183	Serpin A3-2	-	+	-	0.000
184	Cadherin-11	+	+	-	0.005
185	VASN protein	-	+	-	0.027
186	Immunoglobulin superfamily containing leucine-rich repeat protein	-	+	-	0.015
187	Prolyl endopeptidase FAP	-	+	-	0.000
188	Alpha-actinin-4	+	+	-	0.047
189	PCDHGC3 protein	-	+	-	0.003

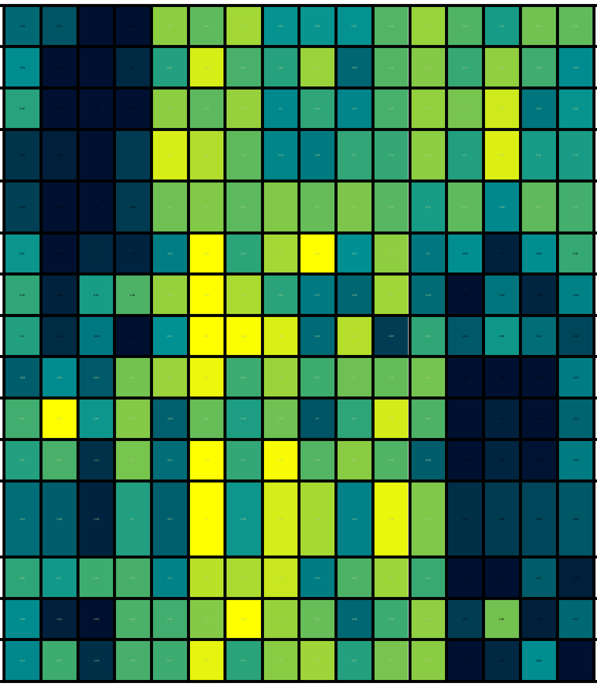

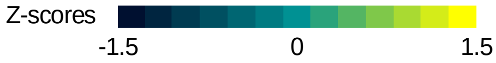

Supplementary Figure 7. Secretomics analysis of human proteins only. Z scores and the results of t tests of the different proteins identified in the different culture conditions: i) EVT-mono, ii) EC-mono, iii) CO, and iv) TRI (two-sided t-test).

No.	Description from fasta	Peptide Hits	EVT-mono				EC-mono				CO				TRI				T tests		
			1	2	3	4	1	2	3	4	1	2	3	4	1	2	3	4	EVT vs CO	EC vs CO	CO vs TRI
1	Proteasome subunit alpha type-2 (Fragment)	1																	0.043	0.019	0.245
2	Tubulin beta chain	4																	0.230	0.000	0.155
3	Thioredoxin reductase 1, cytoplasmic	1																	0.027	0.002	0.627
4	Collagen alpha-2(I) chain	10																	0.573	0.002	0.118
5	Proteasome subunit beta type-2	2																	0.648	0.142	0.161
6	Collagen alpha-1(VI) chain	20																	0.209	0.027	0.169
7	Cell adhesion molecule 1	1																	0.291	0.009	0.029
8	Histone H2A	2																	0.393	0.068	0.013
9	Receptor-type tyrosine-protein phos-phatase S	1																	0.33	0	0.944
10	Peroxiredoxin-1 (Fragment)	3																	0.340	0.012	0.045
11	Translationally-controlled tumor protein	2																	0.769	0.001	0.029
12	Neurobeachin	1																	0.177	0.118	0.175
13	Carboxylic ester hydrolase	1																	0.017	0.006	0.776
14	Isocitrate dehydrogenase [NADP] cyto-plasmic	4																	0.015	0.003	0.913
15	Tropomyosin alpha-4 chain	3																	0.010	0.015	0.067
16	Fructose-bisphosphate aldolase	5																	0.582	0.000	0.488
17	Collagen alpha-1(IV) chain (Fragment)	3																	0.977	0.001	0.117
18	Serine protease HTRA1	2																	0.932	0.000	0.332
19	Histone H4	3																	0.009	0.457	0.771
20	Neogenin	4																	0	0.294	0.240
21	Protein disulfide-isomerase	5																	0.001	0.288	0.105
22	Interleukin-1 receptor accessory pro-tein (Fragment)	3																	0.011	0.321	0.008
23	Phosphoglucomutase-1	2																	0.152	0.030	0.581
24	Lactoylglutathione lyase	1																	0.001	0.877	0.092
25	Plastin-2	4																	0.001	0.913	0.009
26	Vasodilator-stimulated phosphoprotein	2																	0.041	0.785	0.822

27	Cadherin-6	3		0.046	0.597	0.694
28	Adenosine kinase (Fragment)	2		0.094	0.376	0.832
29	ADP/ATP translocase 1	1		0	0.022	0.077
30	Plexin domain-containing protein 2	3		0.008	0.172	0.124
31	Ferritin (Fragment)	1		0.008	0.813	0.138
32	Proteoglycan 4	3		0.064	0.091	0.047
33	14-3-3 protein epsilon	4		0.001	0.223	0.05
34	Alpha-1,4 glucan phosphorylase	3		0.163	0.847	0.262
35	Tubulin alpha-4A chain	12		0	0.129	0.651
36	Polyubiquitin-B (Fragment)	2		0.015	0.275	0.484
37	Histone H3	1		0.019	0.271	0.191
38	C-type mannose receptor 2	3		0.002	0.882	0.269
39	Dickkopf-related protein 3	1		0.005	0.720	0.36
40	L-lactate dehydrogenase	11		0.003	0.208	0.286
41	Epidermal growth factor receptor	1		0.055	0.504	0.189
42	Contactin-1	8		0.019	0.713	0.701
43	Protein kinase C-binding protein NELL2 (Fragment)	2		0.010	0.847	0.418
44	Histone H2B	4		0.023	0.119	0.953
45	Alpha-actinin-1	8		0.285	0.007	0.939
46	Neural cell adhesion molecule 1	4		0.006	0.282	0.292
47	Destrin	2		0.001	0.155	0.546
48	Cadherin-13	3		0.057	0.478	0.029
49	EGF-containing fibulin-like extracellular matrix protein 2	2		0.026	0.703	0.860
50	Rab GDP dissociation inhibitor beta	6		0.004	0.749	0.234
51	Follistatin-related protein 1	4		0.031	0.324	0.836
52	Annexin A5	2		0	0.617	0.878
53	Calsyntenin-1 (Fragment)	1		0.01	0.117	0.962
54	SPARC	13		0.001	0.012	0.019
55	Homogentisate 1,2-dioxygenase	5		0.024	0.433	0.384
56	Voltage-dependent calcium channel subunit alpha-2/delta-1	10		0.008	0.335	0.828
57	Xaa-Pro dipeptidase (Fragment)	1		0.010	0.439	0.180
58	Integrin alpha-2	1		0.023	0.258	0.852
59	6-phosphogluconate dehydrogenase, decarboxylating (Fragment)	1		0.124	0.121	0.242

60	Betaine--homocysteine S-methyltrans-ferase 1	3	0.089	0.095	0.046
61	Nucleobindin-1 (Fragment)	1	0.003	0.010	0.247
62	Myosin light polypeptide 6	3	0.293	0.004	0.207
63	Olfactomedin-like protein 3	1	0.003	0.668	0.907
64	Malate dehydrogenase, cytoplasmic	2	0.088	0.068	0.066
65	Tubulin alpha-1A chain	12	0.024	0.575	0.899
66	Insulin-like growth factor II	2	0.958	0.070	0.158
67	IL6ST nirs variant 3	1	0.294	0.098	0.401
68	Cadherin-11	2	0.456	0.848	0.016
69	Alpha-actinin-4	8	0.143	0.181	0.154

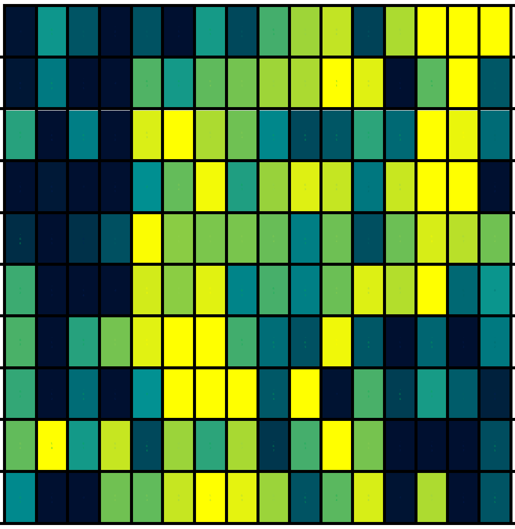

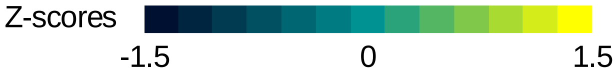

Supplementary Figure 8. Heatmap depicting the human proteins significantly regulated in abundance in the different culture conditions: i) EVT-mono, ii) EC-mono, iii) CO, and iv) TRI

No.	Description from fasta	EVT-mono	EC-mono	CO	TRI
1	Alpha-1,4 glucan phosphorylase				
2	Phosphoglucomutase-1				
3	Proteoglycan 4				
4	Histone H4				
5	Neogenin				
6	Protein disulfide-isomerase				
7	Interleukin-1 receptor accessory protein (Fragment)				
8	Lactoylglutathione lyase				
9	Plastin-2				
10	Vasodilator-stimulated phosphoprotein				
11	Cadherin-6				
12	Adenosine kinase (Fragment)				
13	ADP/ATP translocase 1				
14	Plexin domain-containing protein 2				
15	Ferritin (Fragment)				
16	14-3-3 protein epsilon				
17	Tubulin alpha-4A chain				
18	Fructose-bisphosphate aldolase				
19	Translationally-controlled tumor protein				
20	Tubulin beta chain				
21	Collagen alpha-2(I) chain				
22	Receptor-type tyrosine-protein phosphatase S				
23	Collagen alpha-1(IV) chain (Fragment)				
24	Serine protease HTRA1				
25	Proteasome subunit alpha type-2 (Fragment)				
26	Cell adhesion molecule 1				
27	Peroxiredoxin-1 (Fragment)				
28	Proteasome subunit beta type-2				
29	Histone H2A				
30	Thioredoxin reductase 1, cytoplasmic				
31	Collagen alpha-1(VI) chain				
32	Neurobeachin				
33	Carboxylic ester hydrolase				
34	Isocitrate dehydrogenase [NADP] cytoplasmic				
35	Tropomyosin alpha-4 chain				
36	Alpha-actinin-1				
37	Neural cell adhesion molecule 1				
38	Annexin A5				

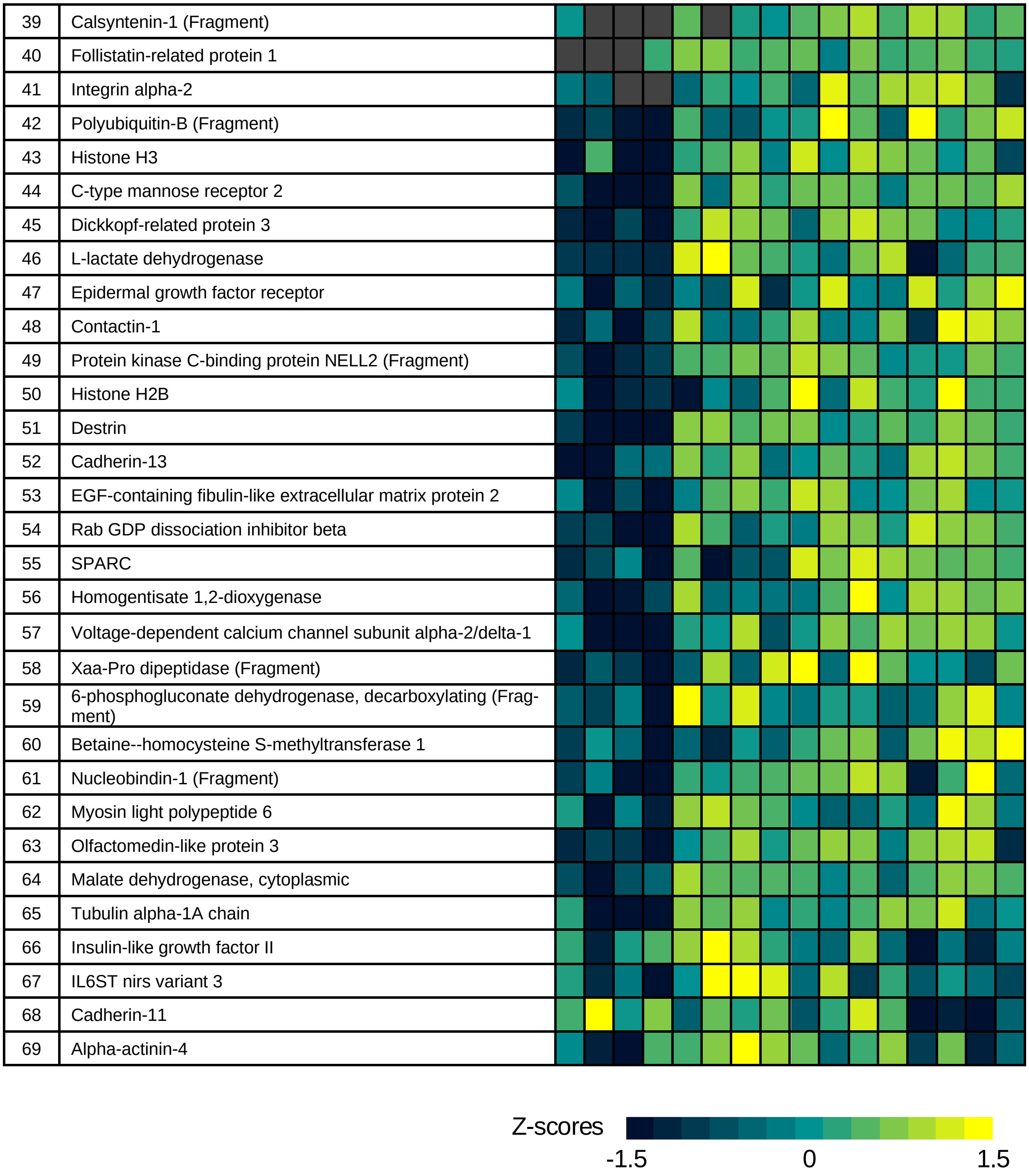
39 Calsyntenin-1 (Fragment)
40 Follistatin-related protein 1
41 Integrin alpha-2
42 Polyubiquitin-B (Fragment)
43 Histone H3
44 C-type mannose receptor 2
45 Dickkopf-related protein 3
46 L-lactate dehydrogenase
47 Epidermal growth factor receptor
48 Contactin-1
49 Protein kinase C-binding protein NELL2 (Fragment)
50 Histone H2B
51 Destrin
52 Cadherin-13
53 EGF-containing fibulin-like extracellular matrix protein 2
54 Rab GDP dissociation inhibitor beta
55 SPARC
56 Homogentisate 1,2-dioxygenase
57 Voltage-dependent calcium channel subunit alpha-2/delta-1
58 Xaa-Pro dipeptidase (Fragment)
59 6-phosphogluconate dehydrogenase, decarboxylating (Fragment)
60 Betaine--homocysteine S-methyltransferase 1
61 Nucleobindin-1 (Fragment)
62 Myosin light polypeptide 6
63 Olfactomedin-like protein 3
64 Malate dehydrogenase, cytoplasmic
65 Tubulin alpha-1A chain
66 Insulin-like growth factor II
67 IL6ST nirs variant 3
68 Cadherin-11
69 Alpha-actinin-4
Z-scores
-1.5
0
1.5

Supplementary Figure 9. List of proteins showing significant increase or decrease between EVT monoculture (EVT-mono) and coculture of EVTs and ECs (CO) (two-sided t-test).

Uniquely expressed proteins in CO against EVT-mono													
No.	Protein name	Peptide Hits	EVT-mono				CO				P value	Known role in implantation/ placental development?	Ref.
			1	2	3	4	1	2	3	4			
1	Neural cell adhesion molecule 1	4									0.000	Yes	S14
2	Annexin A5	2									0.021	Yes	S15
3	Protein S100-A9	3									0.100	Yes	S16
4	Actin, cytoplasmic 1	27									0.286	Yes	S17
5	Alpha-2-macroglobulin	11									0.312	Yes	S18
6	Golgi-associated plant pathogenesis-related protein 1	1									0.352	No	
7	Thioredoxin	3									0.785	Yes	S19

Up-regulated proteins in CO against EVT-mono													
No.	Protein name	Peptide Hits	EVT-mono				CO				P value	Known role in implantation /placental development?	Ref.
			1	2	3	4	1	2	3	4			
1	Annexin A5	2									0	Yes	S15
2	Calsyntenin-1 (Fragment)	1									0.01	No	
3	Follistatin-related protein 1	4									0.031	Yes	S20
4	Integrin alpha-2	1									0.023	Yes	S21
5	Neural cell adhesion molecule 1	4									0.006	Yes	S14
6	Triosephosphate isomerase	7									0.002	No	
7	SPARC	13									0.001	Yes	S22
8	Protein kinase C-binding protein NELL2 (Fragment)	2									0.010	No	
9	Histone H2B	4									0.023	Yes	S23
10	Moesin	4									0.003	Yes	S24
11	C-type mannose receptor 2	3									0.002	Yes	S25
12	Destrin	2									0.001	No	
13	Histone H3	1									0.019	Yes	S26
14	Dickkopf-related protein 3	1									0.005	Yes	S27
15	Thioredoxin reductase 1, cytoplasmic	3									0.027	Yes	S28
17	Rab GDP dissociation inhibitor beta	6									0.004	No	

22	Voltage-dependent calcium channel subunit alpha-2/delta-1	10									0.008	No	
24	Polyubiquitin-B (Fragment)	2									0.015	Yes	S29
25	Isocitrate dehydrogenase [NADP] cytoplasmic	4									0.015	Yes	S30
26	Proteasome subunit alpha type	2									0.005	No	
27	L-lactate dehydrogenase	11									0.003	Yes	S31
28	Collagen alpha-1(IX) chain	2									0.004	Yes	S32
29	Tubulin alpha-1A chain	12									0.024	Yes	S33
30	Contactin-1	8									0.019	Yes	S34
31	DnaJ homolog subfamily C member 13	1									0.046	No	
15	Olfactomedin-like protein 3	1									0.003	No	
16	EGF-containing fibulin-like extracellular matrix protein 2	11									0.026	No	
17	Proteasome subunit alpha type-2 (Fragment)	1									0.043	No	
18	Homogentisate 1,2-dioxygenase	5									0.024	No	
19	Nucleobindin-1 (Fragment)	1									0.003	No	
20	Carboxylic ester hydrolase	1									0.017	No	
21	Xaa-Pro dipeptidase (Fragment)	1									0.010	No	

Down-regulated proteins in CO against EVT-mono

No.	Protein name	Peptide Hits	EVT-mono				CO				P value	Known role in implantation /placental development?	Ref.
			1	2	3	4	1	2	3	4			
1	Cadherin-6	3									0.046	Yes	S35
2	Metalloproteinase inhibitor 1	5									0.019	Yes	S36
3	Plexin domain-containing protein 2	3									0.008	No	
4	Ferritin (Fragment)	1									0.008	Yes	S37
5	Basement membrane-specific heparan sulfate proteoglycan core protein	16									0.047	Yes	S38
6	14-3-3 protein epsilon	4									0.001	Yes	S39
7	Histone H4	3									0.009	Yes	S40
8	Vasodilator-stimulated phosphoprotein	2									0.041	Yes	S41
9	Protein disulfide-isomerase	5									0.001	Yes	S42
10	Neogenin	4									0	Yes	S43
11	Lactoylglutathione lyase	1									0.001	Yes	S44

12	ADP/ATP translocase 1	1									0	No	
13	Interleukin-1 receptor accessory protein (Fragment)	3									0.011	Yes	S45
14	Plastin-2	4									0.001	Yes	S46
15	Tubulin alpha-4A chain	12									0	No	

Z-scores -1.5 0 1.5

Supplementary Figure 10. List of proteins showing significant increase or decrease between EC monoculture (EC-mono) and coculture of EVT and EC (CO) (two-sided t-test).

Up-regulated proteins in CO against EC-mono													
No.	Protein name	Peptide Hits	EC-mono				CO				P value	Known role in implantation/ placental development?	Ref.
			1	2	3	4	1	2	3	4			
1	Serine protease HTRA1	2									0.000	Yes	S47
2	Peroxiredoxin-1 (Fragment)	3									0.012	Yes	S48
3	Receptor-type tyrosine-protein phosphatase S	1									0	Yes	S49
4	Translationally-controlled tumor protein	2									0.001	Yes	S50
5	Collagen alpha-1(IV) chain (Fragment)	3									0.001	Yes	S51
6	Tubulin beta chain	13									0.000	Yes	S52
7	Collagen alpha-2(I) chain	10									0.002	Yes	S53
8	Golgi-associated plant pathogenesis-related protein 1	1									0.025	No	
9	Tropomyosin alpha-4 chain	3									0.015	Yes	S54
10	Cell adhesion molecule 1	1									0.009	Yes	S55
11	Fructose-bisphosphate aldolase	5									0.000	Yes	S56
12	Triosephosphate isomerase	7									0.048	No	
13	Antithrombin-III	11									0.015	Yes	S57
14	SPARC	13									0.012	Yes	S22
15	ADP/ATP translocase 1	1									0.022	No	
16	Thioredoxin reductase 1, cytoplasmic	1									0.002	Yes	S28
17	Reticulocalbin-3	5									0.028		S58
18	Phosphoglucomutase-1	2									0.030	Yes	S59
19	Isocitrate dehydrogenase [NADP] cytoplasmic	4									0.004	Yes	S30
20	Nidogen-1	7									0.003	Yes	S60
21	Biglycan	3									0.013	Yes	S61
22	Proteasome subunit alpha type	2									0.015	No	
23	Collagen alpha-1(VI) chain	20									0.027	Yes	S51
24	Proteasome subunit alpha type-2 (Fragment)	1									0.019	No	
25	Alpha-actinin-1	8									0.007	No	
26	Carboxylic ester hydrolase	1									0.006	No	
27	GTP-binding nuclear protein Ran	2									0.025	No	

28	Nucleobindin-1 (Fragment)	1									0.010	No	

	Down-regulated proteins in CO against EC-mono												
No.	Protein name	Peptide Hits	EC-mono				CO				P value	Known role in implantation/ placental devel- opment?	Ref.
			1	2	3	4	1	2	3	4			
1	Myosin light polypeptide 6	3									0.004	Yes	S39

Z-scores

-1.5 0 1.5

Supplementary Figure 11. Fourteen significantly upregulated endothelial proteins in the coculture configuration of the implantation-on-a-chip.

Endothelial Proteins										
No.	Protein Name	Peptide Hits	EC-Mono				Co			
			1	2	3	4	1	2	3	4
1	Thrombospondin-4	5								
2	Pantetheinase	3								
3	Coagulation factor X	4								
4	Apolipoprotein B-100	6								
5	Insulin-like growth factor-binding protein 2	10								
6	Rab GDP dissociation inhibitor alpha	12								
7	Parkinson disease protein 7	4								
8	Collagen alpha-1(VI) chain	37								
9	Apolipoprotein C-III	1								
10	Collagen alpha-1(III) chain	19								
11	Collagen alpha-3(VI) chain	4								
12	Prolow-density lipoprotein receptor-related protein 1	52								
13	Retinol-binding protein	5								
14	Reticulocalbin-3	8								

Supplementary Figure 12. Twelve significantly upregulated EVT proteins in coculture configuration of the implantation-on-a-chip.

EVT Proteins										
No.	Protein Name	Peptide Hits	EVT-Mono				Co			
			1	2	3	4	1	2	3	4
1	Thrombospondin-4	5								
2	Pantetheinase	3								
3	Coagulation factor X	4								
4	Collagen alpha-1(II) chain	4								
5	Apolipoprotein B-100	6								
6	von Willebrand factor	122								
7	Thrombospondin-1	13								
8	Insulin-like growth factor-binding protein 2	10								
9	Contactin-1	7								
10	Coactosin-like protein	5								
11	Parkinson disease protein 7	4								
12	Dipeptidyl peptidase 2	9								

Supplementary Figure 13. Viability and marker expression of DSCs and additional cell types in the implantation-on-a-chip. (a) Quantification of the viability of DSCs and additional cell types used in the implantation-on-a-chip after 6 days of device culture. Data were obtained from LIVE/DEAD viability assay using Calcein-AM and ethidium homodimer-1 (EthD-1). Data represent mean ± SD from three independent devices (n=3). One-way ANOVA with Tukey's multiple comparison test. (b) Immunofluorescence staining of cell-type-specific marker expression by DSCs and NHLFs used in the implantation-on-a-chip after 6 days of device culture. The representative images are from three independent experiments. Scale bars, 100 μm.

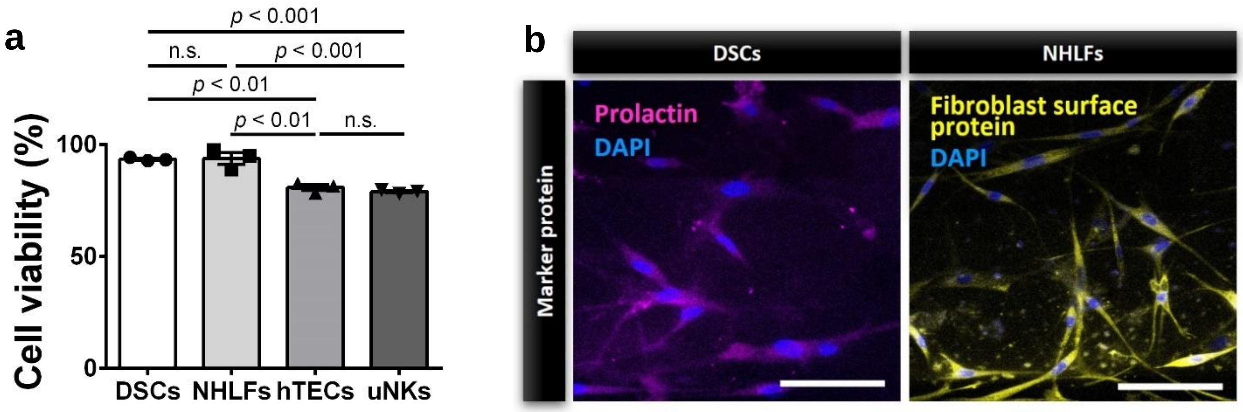

Supplementary Table 1. Cells and authentication methods.

Cells	Vendor	Catalog number	Authentication method
HTR-8/SVneo	ATCC	CRL-3271	STR profiling in July 2016;showed a distinctly human profile (TH01: 6, 9.3;D5S818: 12;D13S317: 9, 12;D7S820: 12;D16S539: 13;CSF1PO: 12;Amelogenin: X;vWA: 13, 18;TPOX:8).
Human endometrial microvascular endothelial cells (HEMEC)	ScienCell	7010	Phenotypic and immunofluorescent characterization by company in March 2015; correct morphology and von Willebrand Factor VIII expression. HEMECs were tested negative for HIV, HBV, HCV, mycoplasma, bacteria, and fungi.
Human lung microvascular endothelial cells (HMVEC-L)	Lonza	CC-2527	Phenotypic and immunofluorescent characterization by company using von Willebrand Factor VIII.
Human brain microvascular endothelial cells (HBMVEC)	AngioPorteomie	CAP-0002	Immunofluorescent characterization by company with antibodies specific to von Willebrand Factor VIII, Di-I-Acetylated-low density lipoprotein, and PECAM1.
Normal human lung fibroblasts	Lonza	CC-2512	Immunofluorescent characterization by company with negative expression for Factor VIII, Cytokeratin 18, and Cytokeratin 19.
Human tracheal epithelial cells	PromoCell	C-12644	Flow cytometric characterization by company using positive expression of Cytokeratin.

Supplementary Table 2. Primary and secondary antibodies for immunofluorescence staining.

Primary antibody [Clone]	Species	Dilution	Product information	Secondary antibody	Dilution
CD31 [P2B1]	Mouse	1:200	Abcam #ab24590	Goat Anti-Mouse IgG (H+L) (Alexa Fluor® Plus 555)	1:200
VE-Cadherin [D87F2]	Rabbit	1:200	Cell Signaling #2500	Goat Anti-Rabbit IgG H&L (Alexa Fluor® 647)	1:200
Cleaved Caspase-3 [Asp175]	Rabbit	1:200	Cell Signaling #9661	Goat Anti-Mouse IgG H&L (Alexa Fluor® 647)	1:200
Ki67 [37C7-12]	Mouse	1:200	Abcam #ab245113	Goat Anti-Mouse IgG H&L (Alexa Fluor® 647)	1:200
Cytokeratin [EPR17078]	Rabbit	1:100	Abcam #ab181598	Goat Anti-Rabbit IgG H&L (Alexa Fluor® 647)	1:200
HLA G [MEM-G/9]	Mouse	1:100	BioRad #MCA2044	Goat Anti-Mouse IgG H&L (Alexa Fluor® 647)	1:100
VCAM-1/CD106 [6G9]	Mouse	1:200	Novus Biolog-icals #NBP1-47491	Goat Anti-Mouse IgG H&L (Alexa Fluor® 647)	1:200
Complement C4 [JM88-13]	Rabbit	1:200	Thermo Fisher #MA5-32856	Goat Anti-Rabbit IgG H&L (Alexa Fluor® 647)	1:200
Prolactin [PRL02]	Mouse	1:200	Thermo Fisher #MA5-11998	Goat Anti-Mouse IgG H&L (Alexa Fluor® 647)	1:200
Fibroblast Surface Protein [1B10]	Mouse	1:200	Abcam #ab11333	Goat Anti-Mouse IgG H&L (Alexa Fluor® 647)	1:200

Supplementary References

1. Blankenship, T. N. & King, B. F. Macaque intra-arterial trophoblast and extravillous trophoblast of the cell columns and cytotrophoblastic shell express neural cell adhesion molecule (NCAM). *Anat Rec* **245**, 525-531, doi:10.1002/(SICI)1097-0185(199607)245:3<525::AID-AR9>3.0.CO;2-Q (1996).
2. Southcombe, J. H., Redman, C. W., Sargent, I. L. & Granne, I. Interleukin-1 family cytokines and their regulatory proteins in normal pregnancy and pre-eclampsia. Clin *Exp Immunol* **181**, 480-490, doi:10.1111/cei.12608 (2015).
3. Qin, F., Tian, J., Zhou, D. & Chen, L. Mst1 and Mst2 kinases: regulations and diseases. *Cell Biosci* **3**, 31, doi:10.1186/2045-3701-3-31 (2013).
4. Menkhorst, E. M. et al. Decidual-secreted factors alter invasive trophoblast membrane and secreted proteins implying a role for decidual cell regulation of placentation. *PLoS One* **7**, e31418, doi:10.1371/journal.pone.0031418 (2012).
5. Sferruzzi-Perri, A. N., Sandovici, I., Constancia, M. & Fowden, A. L. Placental phenotype and the insulin-like growth factors: resource allocation to fetal growth. *J Physiol* **595**, 5057-5093, doi:10.1113/JP273330 (2017).
6. Liu, B., Liu, L., Cui, S., Qi, Y. & Wang, T. Expression and significance of microRNA-126 and VCAM-1 in placental tissues of women with early-onset preeclampsia. *J Obstet Gynaecol Res* **47**, 2042-2050, doi:10.1111/jog.14732 (2021).
7. Giudice, L. C. et al. Identification and regulation of the IGFBP-4 protease and its physiological inhibitor in human trophoblasts and endometrial stroma: evidence for paracrine regulation of IGF-II bioavailability in the placental bed during human implantation. *J Clin Endocrinol Metab* **87**, 2359-2366, doi:10.1210/jcem.87.5.8448 (2002).
8. Taracha, A., Kotarba, G. & Wilanowski, T. Neglected Functions of TFCP2/TFCP2L1/UBP1 Transcription Factors May Offer Valuable Insights into Their Mechanisms of Action. *Int J Mol Sci* ***19***, doi:10.3390/ijms19102852 (2018).
9. Altmae, S. et al. Endometrial gene expression analysis at the time of embryo implantation in women with unexplained infertility. *Molecular human reproduction* **16**, 178-187, doi:10.1093/molehr/gap102 (2010).
10. Flood-Nichols, S. K. et al. Elevated ratio of maternal plasma ApoCIII to ApoCII in preeclampsia. *Reprod Sci* **18**, 493-502, doi:10.1177/1933719110390390 (2011).
11. Wen, H., Hu, Y., Chen, L., Zhao, L. & Yang, X. miR-338-5p Targets Epidermal Growth Factor-Containing Fibulin-Like Extracellular Matrix Protein 1 to Inhibit the Growth and Invasion of Trophoblast Cells in Selective Intrauterine Growth Restriction. *Reprod Sci* **27**, 1357-1364, doi:10.1007/s43032-020-00160-3 (2020).
12. Pierzchalski, K. et al. Retinoic acid biosynthesis is impaired in human and murine endometriosis. *Biology of reproduction* **91**, 84, doi:10.1095/biolreprod.114.119677 (2014).
13. Altmae, S., Kallak, T. K., Friden, B. & Stavreus-Evers, A. Variation in hyaluronan-binding protein 2 (HABP2) promoter region is associated with unexplained female infertility. *Reprod Sci* **18**, 485-492, doi:10.1177/1933719110388849 (2011).
14. Blankenship, T. N. & King, B. F. Macaque intra-arterial trophoblast and extravillous trophoblast of the cell columns and cytotrophoblastic shell express neural cell adhesion molecule (NCAM). *Anat Rec* **245**, 525-531, doi:10.1002/(SICI)1097-0185(199607)245:3<525::AID-AR9>3.0.CO;2-Q (1996).
15. Degrelle, S. A., Gerbaud, P., Leconte, L., Ferreira, F. & Pidoux, G. Annexin-A5 organized in 2D-network at the plasmalemma eases human trophoblast fusion. *Sci Rep* **7**, 42173, doi:10.1038/srep42173 (2017).

16. Nair, R. R., Khanna, A. & Singh, K. Role of inflammatory proteins S100A8 and S100A9 in pathophysiology of recurrent early pregnancy loss. *Placenta* **34**, 824-827, doi:10.1016/j.placenta.2013.06.307 (2013).
17. Thie, M. et al. Adhesiveness of the free surface of a human endometrial monolayer for trophoblast as related to actin cytoskeleton. *Mol Hum Reprod* **3**, 275-283, doi:10.1093/molehr/3.4.275 (1997).
18. Esadeg, S., He, H., Pijnenborg, R., Van Leuven, F. & Croy, B. A. Alpha-2 macroglobulin controls trophoblast positioning in mouse implantation sites. *Placenta* **24**, 912-921, doi:10.1016/s0143-4004(03)00148-6 (2003).
19. Tonissen, K. et al. Site-directed mutagenesis of human thioredoxin. Identification of cysteine 74 as critical to its function in the "early pregnancy factor" system. *J Biol Chem* **268**, 22485-22489 (1993).
20. Mouillet, J. F. et al. The expression and post-transcriptional regulation of FSTL1 transcripts in placental trophoblasts. *Placenta* **36**, 1231-1238, doi:10.1016/j.placenta.2015.09.005 (2015).
21. Lee, C. Q. E. et al. Integrin alpha2 marks a niche of trophoblast progenitor cells in first trimester human placenta. *Development* **145**, doi:10.1242/dev.162305 (2018).
22. Jiang, Y. et al. Downregulation of SPARC expression inhibits the invasion of human trophoblast cells in vitro. *PLoS One* **8**, e69079, doi:10.1371/journal.pone.0069079 (2013).
23. Kohan-Ghadr, H. R., Kadam, L., Jain, C., Armant, D. R. & Drewlo, S. Potential role of epigenetic mechanisms in regulation of trophoblast differentiation, migration, and invasion in the human placenta*. Cell adhesion & migration* **10**, 126-135, doi:10.1080/19336918.2015.1098800 (2016).
24. Matsumoto, H., Daikoku, T., Wang, H., Sato, E. & Dey, S. K. Differential expression of ezrin/radixin/moesin (ERM) and ERM-associated adhesion molecules in the blastocyst and uterus suggests their functions during implantation. *Biology of reproduction* **70**, 729-736, doi:10.1095/biolreprod.103.022764 (2004).
25. Ono, Y. et al. CD206+ M2-Like Macrophages Are Essential for Successful Implantation. *Front Immunol* **11**, 557184, doi:10.3389/fimmu.2020.557184 (2020).
26. Jang, C. W., Shibata, Y., Starmer, J., Yee, D. & Magnuson, T. Histone H3.3 maintains genome integrity during mammalian development. *Genes Dev* **29**, 1377-1392, doi:10.1101/gad.264150.115 (2015).
27. Wu, Y. et al. Serum biomarker analysis in patients with recurrent spontaneous abortion. *Mol Med Rep* **16**, 2367-2378, doi:10.3892/mmr.2017.6890 (2017).
28. Jakupoglu, C. et al. Cytoplasmic thioredoxin reductase is essential for embryogenesis but dispensable for cardiac development. *Mol Cell Biol* **25**, 1980-1988, doi:10.1128/MCB.25.5.1980-1988.2005 (2005).
29. Wang, H. M. et al. Effect of ubiquitin-proteasome pathway on mouse blastocyst implantation and expression of matrix metalloproteinases-2 and -9. *Biol Reprod* **70**, 481-487, doi:10.1095/biolreprod.103.021634 (2004).
30. Rosario, F. J. et al. Characterization of the Primary Human Trophoblast Cell Secretome Using Stable Isotope Labeling With Amino Acids in Cell Culture. *Front Cell Dev Biol* **9**, 704781, doi:10.3389/fcell.2021.704781 (2021).
31. Auerbach, S. & Brinster, R. L. Lactate dehydrogenase isozymes in the early mouse embryo. *Exp Cell Res* **46**, 89-92, doi:10.1016/0014-4827(67)90411-9 (1967).
32. Hannan, N. J. & Salamonsen, L. A. CX3CL1 and CCL14 regulate extracellular matrix and adhesion molecules in the trophoblast: potential roles in human embryo implantation. *Biol Reprod* **79**, 58-65, doi:10.1095/biolreprod.107.066480 (2008).

33. Shukla, V. et al. Microtubule depolymerization attenuates WNT4/CaMKIIalpha signaling in mouse uterus and leads to implantation failure. *Reproduction* **158**, 47-59, doi:10.1530/REP-18-0611 (2019).
34. Tapia, A., Salamonsen, L. A., Manuelpillai, U. & Dimitriadis, E. Leukemia inhibitory factor promotes human first trimester extravillous trophoblast adhesion to extracellular matrix and secretion of tissue inhibitor of metalloproteinases-1 and -2. *Hum Reprod* **23**, 1724-1732, doi:10.1093/humrep/den121 (2008).
35. Zhou, W., Santos, L. & Dimitriadis, E. Characterization of the role for cadherin 6 in the regulation of human endometrial receptivity. *Reprod Biol Endocrinol* **18**, 66, doi:10.1186/s12958-020-00624-w (2020).
36. Zhu, J. Y., Pang, Z. J. & Yu, Y. H. Regulation of trophoblast invasion: the role of matrix metalloproteinases. *Rev Obstet Gynecol* **5**, e137-143 (2012).
37. Ng, S. W., Norwitz, S. G. & Norwitz, E. R. The Impact of Iron Overload and Ferroptosis on Reproductive Disorders in Humans: Implications for Preeclampsia. *Int J Mol Sci* **20**, doi:10.3390/ijms20133283 (2019).
38. Chui, A. et al. The expression of placental proteoglycans in pre-eclampsia. *Gynecol Obstet Invest* **73**, 277-284, doi:10.1159/000333262 (2012).
39. Gonzalez, R., Mohan, H. & Unniappan, S. Nucleobindins: bioactive precursor proteins encoding putative endocrine factors? *Gen Comp Endocrinol* **176**, 341-346, doi:10.1016/j.ygcen.2011.11.021 (2012).
40. Ghule, P. N. et al. Maternal expression and early induction of histone gene transcription factor Hinfp sustains development in pre-implantation embryos. *Dev Biol* **419**, 311-320, doi:10.1016/j.ydbio.2016.09.003 (2016).
41. Kayisli, U. A., Selam, B., Demir, R. & Arici, A. Expression of vasodilator-stimulated phosphoprotein in human placenta: possible implications in trophoblast invasion. *Mol Hum Reprod* **8**, 88-94, doi:10.1093/molehr/8.1.88 (2002).
42. Fernando, S. R. et al. Expression of membrane protein disulphide isomerase A1 (PDIA1) disrupt a reducing microenvironment in endometrial epithelium for embryo implantation. *Exp Cell Res* **405**, 112665, doi:10.1016/j.yexcr.2021.112665 (2021).
43. Zhong, S., Zou, L., Zhao, Y., Hu, B. & Xie, H. Effect of different concentrations of neogenin on proliferation, apoptosis and related proliferative factors in human trophoblasts. *J Huazhong Univ Sci Technolog Med Sci* **30**, 500-504, doi:10.1007/s11596-010-0457-x (2010).
44. Aouache, R., Biquard, L., Vaiman, D. & Miralles, F. Oxidative Stress in Preeclampsia and Placental Diseases. *Int J Mol Sci* **19**, doi:10.3390/ijms19051496 (2018).
45. Southcombe, J. H., Redman, C. W., Sargent, I. L. & Granne, I. Interleukin-1 family cytokines and their regulatory proteins in normal pregnancy and pre-eclampsia. *Clin Exp Immunol* **181**, 480-490, doi:10.1111/cei.12608 (2015).
46. Popovici, R. M. et al. Gene expression profiling of human endometrial-trophoblast interaction in a coculture model. *Endocrinology* **147**, 5662-5675, doi:10.1210/en.2006-0916 (2006).
47. Ajayi, F. et al. Elevated expression of serine protease HtrA1 in preeclampsia and its role in trophoblast cell migration and invasion. *Am J Obstet Gynecol* **199**, 557 e551-510, doi:10.1016/j.ajog.2008.04.046 (2008).
48. Morita, K. et al. Peroxiredoxin as a functional endogenous antioxidant enzyme in pronuclei of mouse zygotes. *J Reprod Dev* **64**, 161-171, doi:10.1262/jrd.2018-005 (2018).
49. Biase, F. H. et al. Fine-tuned adaptation of embryo-endometrium pairs at implantation revealed by transcriptome analyses in Bos taurus. *PLoS Biol* **17**, e3000046, doi:10.1371/journal.pbio.3000046 (2019).

50. Kalra, S. K., Ratcliffe, S. J., Coutifaris, C., Molinaro, T. & Barnhart, K. T. Ovarian stimulation and low birth weight in newborns conceived through in vitro fertilization. *Obstetrics and gynecology* **118**, 863-871, doi:10.1097/AOG.0b013e31822be65f (2011).
51. Shi, J. W. et al. Collagen at the maternal-fetal interface in human pregnancy. Int J Biol Sci 16, 2220-2234, doi:10.7150/ijbs.45586 (2020).
52. Schatten, G., Simerly, C. & Schatten, H. Microtubule configurations during fertilization, mitosis, and early development in the mouse and the requirement for egg microtubule-mediated motility during mammalian fertilization. Proc Natl Acad Sci U S A 82, 4152-4156, doi:10.1073/pnas.82.12.4152 (1985).
53. Kim, T. H. et al. Loss of HDAC3 results in nonreceptive endometrium and female infertility. Sci Transl Med 11, doi:10.1126/scitranslmed.aaf7533 (2019).
54. Paule, S., Li, Y. & Nie, G. Cytoskeletal remodelling proteins identified in fetal-maternal interface in pregnant women and rhesus monkeys. J Mol Histol 42, 161-166, doi:10.1007/s10735-011-9319-5 (2011).
55. Wang, Y., Ma, C. H. & Qiao, J. [Differential expression of microRNA in eutopic endometrium tissue during implantation window for patients with endometriosis related infertility]. Zhonghua Fu Chan Ke Za Zhi 51, 436-441, doi:10.3760/cma.j.issn.0529-567X.2016.06.007 (2016).
56. Myers, J. E. et al. Integrated proteomics pipeline yields novel biomarkers for predicting preeclampsia. Hypertension 61, 1281-1288, doi:10.1161/HYPERTENSIONAHA.113.01168 (2013).
57. Bashiri, A., Halper, K. I. & Orvieto, R. Recurrent Implantation Failure-update overview on etiology, diagnosis, treatment and future directions. Reprod Biol Endocrinol 16, 121, doi:10.1186/s12958-018-0414-2 (2018).
58. Ma, X. et al. Decidual cell polyploidization necessitates mitochondrial activity. PLoS One 6, e26774, doi:10.1371/journal.pone.0026774 (2011).
59. Castel, G. et al. Induction of Human Trophoblast Stem Cells from Somatic Cells and Pluripotent Stem Cells. Cell Rep 33, 108419, doi:10.1016/j.celrep.2020.108419 (2020).
60. Bilban, M. et al. Identification of novel trophoblast invasion-related genes: heme oxygenase-1 controls motility via peroxisome proliferator-activated receptor gamma. Endocrinology 150, 1000-1013, doi:10.1210/en.2008-0456 (2009).
61. San Martin, S. & Zorn, T. M. The small proteoglycan biglycan is associated with thick collagen fibrils in the mouse decidua. Cell Mol Biol (Noisy-le-grand) 49, 673-678 (2003).
