# Supplementary material for: A microphysiological model of human trophoblast invasion during implantation
Source: Nat Commun. 2022 Mar 15;13:1252. doi: 10.1038/s41467-022-28663-4 (PMC8924260; doi:10.1038/s41467-022-28663-4)
Supplement: Supplementary file 2 — Description of Additional Supplementary Files [file 41467_2022_28663_MOESM2_ESM.docx]

Description of Additional Supplementary Files

Title: Supplementary Movie 1.

Description: Top view of the device during injection of ECM hydrogel precursor solution into the center lane. Scale bar, 1 mm

Title: Supplementary Movie 2.

Description: Crosssectional view of the device during injection of ECM hydrogel precursor solution into the center lane. Scale bar, 500 μm.
