# Supplementary material for: A microphysiological model of human trophoblast invasion during implantation
Source: Nat Commun. 2022 Mar 15;13:1252. doi: 10.1038/s41467-022-28663-4 (PMC8924260; doi:10.1038/s41467-022-28663-4)
Supplement: Supplementary file 5 — Reporting Summary [file 41467_2022_28663_MOESM5_ESM.pdf]

nature research

Corresponding author(s): Dan Dongeun Huh, Monica Mainigi

Last updated by author(s): Jan 20, 2022

Reporting Summary

Nature Research wishes to improve the reproducibility of the work that we publish. This form provides structure for consistency and transparency in reporting. For further information on Nature Research policies, see our Editorial Policies and the Editorial Policy Checklist.

Statistics

For all statistical analyses, confirm that the following items are present in the figure legend, table legend, main text, or Methods section.

n/a	Confirmed	
☐	☒	The exact sample size (n) for each experimental group/condition, given as a discrete number and unit of measurement
☐	☒	A statement on whether measurements were taken from distinct samples or whether the same sample was measured repeatedly
☐	☒	The statistical test(s) used AND whether they are one- or two-sided<br>*Only common tests should be described solely by name; describe more complex techniques in the Methods section.*
☐	☒	A description of all covariates tested
☒	☐	A description of any assumptions or corrections, such as tests of normality and adjustment for multiple comparisons
☐	☒	A full description of the statistical parameters including central tendency (e.g. means) or other basic estimates (e.g. regression coefficient) AND variation (e.g. standard deviation) or associated estimates of uncertainty (e.g. confidence intervals)
☐	☒	For null hypothesis testing, the test statistic (e.g. F, t, r) with confidence intervals, effect sizes, degrees of freedom and P value noted<br>*Give P values as exact values whenever suitable.*
☒	☐	For Bayesian analysis, information on the choice of priors and Markov chain Monte Carlo settings
☒	☐	For hierarchical and complex designs, identification of the appropriate level for tests and full reporting of outcomes
☒	☐	Estimates of effect sizes (e.g. Cohen's d, Pearson's r), indicating how they were calculated

Our web collection on statistics for biologists contains articles on many of the points above.

Software and code

Policy information about availability of computer code

Data collection	Microscopy: Microscopy: Zeiss ZEN 2.3, ImageJ v1.53C (in NIH distribution)
Data analysis	Imaging: ImageJ Particle Analyzer was used to measure the area of a defined fluorescence channel within the image. ImageJ Angle Measurement function was used to measure the angle of the long axis of the cell from the horizontal axis of the image. Zeiss ZEN 2.3 was used for analyzing the intensity of a defined fluorescence channel within the image. Zeiss ZEN 2.3 was used to convert image sequences to video. GraphPad Prism Version 6 was used to run statistical tests and generate graphs. Proteomics: MaxQuant (UniProtKB, downloaded in 2020).searching against the Homo sapiens UniProt database. Data processing was performed in R v3.5.1 using the package RomicsProcessor v1.0.0, which is available on Github (https://github.com/PNNL-Comp-Mass-Spec/RomicsProcessor) and Zenodo (https://doi.org/10.5281/zenodo.3956544). Zenodo was used for data assembly, data visualization, data clustering, data subsetting, normalization and general statistics,in a non-destructive fashion by saving the original data frame, its associated metadata, the transformative steps of the data processing and the processed data in the same multi-layered type of R object named "romics_object ; No custom computer algorithms or custom software were utilized. The MaxQuant results files and the R code used to analyze secretomics and proteomics data were deposited on GitHub and Zenodo [https://doi.org/10.5281/zenodo.5842089 for the secretome and https://doi.org/10.5281/zenodo.5842089 for the cellular proteome]

For manuscripts utilizing custom algorithms or software that are central to the research but not yet described in published literature, software must be made available to editors and reviewers. We strongly encourage code deposition in a community repository (e.g. GitHub). See the Nature Research guidelines for submitting code & software for further information.

Data

Policy information about availability of data

All manuscripts must include a data availability statement. This statement should provide the following information, where applicable:

- Accession codes, unique identifiers, or web links for publicly available datasets
- A list of figures that have associated raw data
- A description of any restrictions on data availability

All data generated in this study are included in the published article and its supplementary information. All additional information is available upon reasonable request to the authors. Raw data from Figures 1-7 and Supplementary Figuers 1-13 is included in the Source data file attached. Proteomics raw data files were deposited on MassIVE (accession code MSV000086888 for the secretome [ftp://massive.ucsd.edu/MSV000086888/] and MSV000088677 for the cellular proteome [ftp://massive.ucsd.edu/ MSV000088677]. Code for the complete data analysis was deposited on github (https://github.com/GeremyClair/Implantation_on_a_chip_Proteomics).

Field-specific reporting

Please select the one below that is the best fit for your research. If you are not sure, read the appropriate sections before making your selection.

- [x] Life sciences
- [ ] Behavioural & social sciences
- [ ] Ecological, evolutionary & environmental sciences

For a reference copy of the document with all sections, see nature.com/documents/nr-reporting-summary-flat.pdf

Life sciences study design

All studies must disclose on these points even when the disclosure is negative.

Sample size	Sample size was not predetermined. We performed at least 3 independent experiments for each tested condition,each containing 3 technical replicates or more. Sample sizes were determined using widely accepted guidelines for 3D cell culture in engineered microphysiological devices, as well as our extensive experience in the development and engineering of physiological in vitro tissue and organ models. In addition, all independent experiments and replication attempts in this study were successful and showed very consistent biological phenomena, which were sufficient to ensure statistical significance. Considering the experimental unit being observed as defined by Lazic et al as extent of invasion by extravillous trophoblasts, and the experiments performed on our device being essentially cell culture experiments, we considered biological replicates as (to the extent possible) independent experiments performed on different days, different passage numbers and cells from different patients. Since no prior information on these variables were available, we made sure at least 3 replicates were performed for each experiment attempted.
Data exclusions	No data were excluded from the study.
Replication	To avoid confounding effects, comparisons were made with technical replicates concurrently established in an identical manner using similar numbers of cells, same solutions of culture media and same commercial lots of primary human cells when applicable. Fetal EVTs from different patients, but similar gestational ages were utilized, and studies using maternal endometrial cells isolated cells from independent patients for each experiment to ensure reproducibility of experimental findings. All human subjects showed similar baseline demographic characteristics with no known pathology. All experiments contained biological (at least 3) and technical replicates (at least 3) to confirm reliable reproducibility of experimental findings, and were successful. No data was excluded.
Randomization	All replicates on organ-on-a-chip-based platform were randomly distributed between experimental groups.
Blinding	Studies utilized well-definted quantitative metrics and did not involve subjective measurements or qualitative rating schema. Researchers were not blinded to allocation of experimental groups, data collection or analysis.

Reporting for specific materials, systems and methods

We require information from authors about some types of materials, experimental systems and methods used in many studies. Here, indicate whether each material, system or method listed is relevant to your study. If you are not sure if a list item applies to your research, read the appropriate section before selecting a response.

Materials & experimental systems

n/a	Involved in the study	
[ ]	[x]	Antibodies
[ ]	[x]	Eukaryotic cell lines
[x]	[ ]	Palaeontology and archaeology
[x]	[ ]	Animals and other organisms
[ ]	[x]	Human research participants
[x]	[ ]	Clinical data
[x]	[ ]	Dual use research of concern

Methods

n/a	Involved in the study	
[x]	[ ]	ChIP-seq
[ ]	[x]	Flow cytometry
[x]	[ ]	MRI-based neuroimaging

Antibodies

Antibodies used

For imaging:
Primary antibodies
Cytokeratin-7(Abcam[EPR17078] ab181598), 1:200;
CD31 (Abcam [PRB1] ab215911), 1:200;
VE-cadherin(Cell Signaling [D87F2] #2500), 1:200;
Caspase-3 (Cell Signaling [Asp175] #9661), 1:200;
Prolactin (Thermo Fisher [PRL02] #MA5-11998), 1:200;
Fibroblast surface protein (Abcam[1B10]ab11333), 1:200;
Ki-67 (Abcam[37C7-12] ab245113), 1:200;
HLAG (Biorad [MEM-G/9] #MCA2044), 1:100;
VCAM-1(Novus Biologicals[6G9] NBP1-47491), 1:200 and
Complement C4(Invitrogen[JM88-13] MA5-32856), 1:200
Secondary antibodies
Goat anti-mouse IgG H&L (Alexa Fluor® 555) (A32727, Thermo Fisher);
Goat anti-mouse IgG H&L (Alexa Fluor® 647) (ab150115, Abcam) and
Goat anti-Rabbit IgG (H+L) (Alexa Fluor® 647) (ab150079, Abcam)

For flow cytometry:
LIVE/DEAD Fixable Aqua Dead Cell Stain (Invitrogen, L34965);
CD45 (2D1,BioLegend, #368503, 1:100);
Ki67 (16A8,BioLegend,#652405,1:100;
HLA-G (biotin MEM-G/9, Invitrogen, #MA1-19513, 1:50), Brilliant Violet 421™ Streptavidin (BioLegend, #405226,1:100).

Validation

Validation data for each antibody used can be found on the manufacturers' websites and datasheets as summarized below. In addition, for imaging, specificity of primary antibodies was validated by negative control staining in cultured cells that are not known to express the target antigen, and for flow cytometry, fluorescence minus one controls were carried out.

CD31 (Abcam # ab24590) is a mouse monoclonal antibody for CD31 of endothelial cells. It was tested for applications in immunohistochemistry stainings for human species.
VE-Cadherin (Cell Signaling #2500) is a rabbit monoclonal antibody for VE-cadherin. It has been validated for western blotting, immunoprecipitation, immunocytochemistry, and flow cytometry for human, bovine, and pig.
Cleaved Caspase-3 (Cell Signaling #9661) is a rabbit polyclonal antibody to human Cleaved-Caspase-3. It has been validated for western blotting, immunoprecipitation, and immunocytochemistry stainings for human, mouse, rabbit, and monkey.
ki67 (Abcam #ab245113) is a mouse monoclonal antibody to KI67. It has been validated for application in flow cytometry and immunocytochemistry stainings for only human species.
Cytokeratin-7 (Abcam # ab181598) is rabbit monoclonal antibody for Cytokeratin 7. It has been validated for western blotting, immunocytochemistry stainings, and flow cytometry for human, mouse, and rat.
VCAM-1(Novus Biologicals[6G9] NBP1-47491) is a mouse monoclonal antibody to VCAM-1. It has been validated for western blotting, immunohistochemistry stainings for human, canine, and primate species.
Complement C4(Thermo Fisher # MA5-32856) is a rabbit monoclonal antibody for Complement C4. It has been validated for use in western blotting, immunohistochemistry stainings for only human species.
Prolactin (Thermo Fisher # MA5-11998) is a mouse monoclonal antibody for prolactin. It has been validated for use in immunohistochemistry stainings and ELISA for only human species.
Fibroblast surface protein (Abcam # ab11333) is a mouse monoclonal antibody for fibroblast surface protein. It has been validated for use in immunohistochemistry stainings and western blotting for only human species..
HLAG (Biorad #MCA2044): As stated on the BioRad website, this product was tested for application in immunofluoresence experiments. No other validation data was listed on website, but we tested validity of antibody by co-staining with cytokeratin 7 and human placental lactogen in first trimester formalin fixed paraffin embedded tissue and found specific expression in extravillous trophoblasts only. In addition, this antibody was tested along with other HLA-G antibody clones to ensure specificity of staining.

Flow cytometry
CD45 (2D1,BioLegend, #368503): Each lot was tested for positive flow cytometry staining as listed on the BioLegend website
Ki67 (16A8,BioLegend,#652405): Each lot was tested for positive intracellular flow cytometry staining using a specific Ki67 staining protocol as listed on the BioLegend website
HLA-G (biotin MEM-G/9, Invitrogen, #MA1-19513, 1:50): Antibody was tested for use in flow cytometry as per manufacturer website.

Eukaryotic cell lines

Policy information about cell lines

Cell line source(s)

The HTR-8/SV neo cell line was obtained from ATCC, catalog # CRL-3271,lot number 64275781.
Human primary cells used included Human endometrial microvascular endothelial cells (HEMEC, #7010, ScienCell); human lung microvascular endothelial cells (HMVEC-L, CC-2527, Lonza), human brain microvascular endothelial cells (HBMVEC,

	cAP-0002, AngioProteomie), human tracheal epithelial cells (HTEpC, C-12644, PromoCell), human lung fibroblast (NHLF, CC-2512, Lonza).
Authentication	Authentication of the HTR-8/SVneo cell line was performed by ATCC using STR profiling in July 2016, and showed a distinctly human profile (TH01: 6, 9.3;D5S818: 12;D13S317: 9, 12;D7S820: 12;D16S539: 13;CSF1PO: 12;Amelogenin: X;vWA: 13, 18;TPOX:8).<br><br>Primary cells were isolated from specified human tissue for research by commercial vendors and authenticated by them as stated on the ScienCell, Lonza, AngioProtemie, and PromoCell websites.<br>Human endometrial microvascular endothelial cells (HEMECs) from ScienCell Research Laboratories were isolated from human uterus and the phenotype charaterization of the cells was confirmed with morphology and von Willebrand Factor VIII expression. HEMECs were tested negative for HIV, HBV, HCV, mycoplasma, bacteria, and fungi.<br>Human brain microvascular endothelial cells (HBMVECs) from AngioProteomie were isolated from human normal human cerebral cortex. HBMVECs were characterized by immunofluorescence with antibodies specific to von Willebrand Factor VIII, Di-I-Acetylated-low density lipoprotein, and PECAM1. And they were tested negative for HIV-1, HBV, HCV, and mycoplasma.<br>Human lung microvascular endothelial cells from Lonza were isolated from lung tissue and the phenotypic characterization of the cells was confirmed by the staining using von Willebrand Factor VIII. The cells are guaranteed to further expand through 15 population doublings under the condition provided by the vendor. The cells were tested negative for HIV, HBV, HCV, and mycoplasma.<br>Human lung fibroblasts (NHLFs) from Lonza were isolated from human lung tissue. NHLFs were characterized with negative expression for Factor VIII, Cytokeratin 18, and Cytokeratin 19. The cells are guaranteed to further expand through 15 population doublings under the condition provided by the vendor. They were tested negative for HIV, HBV, HCV, and mycoplasma.<br>Human tracheal epithelial cells (TECs) from PromoCell were isolated from human tracheal mucosa. TECs were characterized by flow cytometry against Cytokeratin. TECs were tested negative for bacteria, fungi, mycoplasma, HIV-1, HIV-2, HBV, HCV, HTLV-1, and HTLV-2.
Mycoplasma contamination	All commercially purchased cells were reported negative for mycoplasma as tested by the vendor, and were used within one passage of receipt.
Commonly misidentified lines<br>(See ICLAC register)	No commonly misidentified cell lines were used in any experiment.

Human research participants

Policy information about studies involving human research participants

Population characteristics	EVTs were obtained from pregnant adult women (<12 weeks gestation) between 18-45 years of age, undergoing elective pregnancy termination at the Hospital of the University of Pennsylvania. Women with multiple pregnancies or known aneuoploidy were excluded from the study.<br>Endometrial biopsies were obtained from healthy women between 18-43 years of age with no significant medical history, not using hormonal contraception and with regular menstrual cycles. Biopsies were obtained during the mid-luteal phase 7-10 days post-ovulation.
Recruitment	Patients undergoing pregnancy termination were approached during treatment consultation and recruited to the study via informed consent. Subjects for endometrial biopsies were recruited by self-referral through posting of flyers in the local community as well as by contact through the Women's Health Clinical Research Center (WHCRC) database. There might be potential for self-selection bias based on demographic characteristics such as age and race, and income disparity. Demographic characteristics are known to influence pregnancy outcome, and future work will aim to stratify data based on some of these characteristics.
Ethics oversight	All human samples were obtained under active IRBs with oversight by the University of Pennsylvania Institutional Review Board

Note that full information on the approval of the study protocol must also be provided in the manuscript.

Flow Cytometry

Plots

Confirm that:

- [x] The axis labels state the marker and fluorochrome used (e.g. CD4-FITC).
- [x] The axis scales are clearly visible. Include numbers along axes only for bottom left plot of group (a 'group' is an analysis of identical markers).
- [x] All plots are contour plots with outliers or pseudocolor plots.
- [x] A numerical value for number of cells or percentage (with statistics) is provided.

Methodology

Sample preparation	Media was removed from IOC devices before the devices were washed twice with PBS. After removing residual PBS the reservoirs of each channel were filled with 0.25% Trypsin and incubated at 37°C for 15 minutes while pipetting every 5 minutes until the ECM hydrogel in the middle channel was completely dissolved. Cell suspensions were collected, centrifuged at 300 g for 5 min, and then the supernatant was removed using a pipette, leaving only the cell pellet. The cell pellet was resuspended in PBS and fixed with 75% ethanol added dropwise, for 1 hour at -20°C before proceeding to staining.
Instrument	MACSQuant Analyzer 10 (Miltenyi Biotec)
Software	FlowJo 10
Cell population abundance	N/A
Gating strategy	Gating strategy is shown in Supplementary Figure 1b. Live,singlet, CD45-, HLAG+, Ki67+ cells were analyzed

☒ Tick this box to confirm that a figure exemplifying the gating strategy is provided in the Supplementary Information.
